# Correction: Photosymbiont associations persisted in planktic foraminifera during early Eocene hyperthermals at Shatsky Rise (Pacific Ocean)

**DOI:** 10.1371/journal.pone.0293770

**Published:** 2023-10-26

**Authors:** The PLOS ONE Staff

S1 Data is a duplicate of S2 Data. The publisher apologizes for the error. Please view the correct S1 Data below.

## Supporting information

S1 Data Supplementary dataset. Table listing for each sample of the ODP sample name, age, species name, test size class, δ18O and δ13C value, and classification assigned here as “pre”, “peak”, or “post” event as well as the hyperthermal event the sample was associated with.
(XLSX)
