# Supplementary figures and images for: Photosymbiont associations persisted in planktic foraminifera during early Eocene hyperthermals at Shatsky Rise (Pacific Ocean) (part 2 of 3)
Source: PLoS One. 2022 Sep 26;17(9):e0267636. doi: 10.1371/journal.pone.0267636 (PMC9512218; doi:10.1371/journal.pone.0267636)

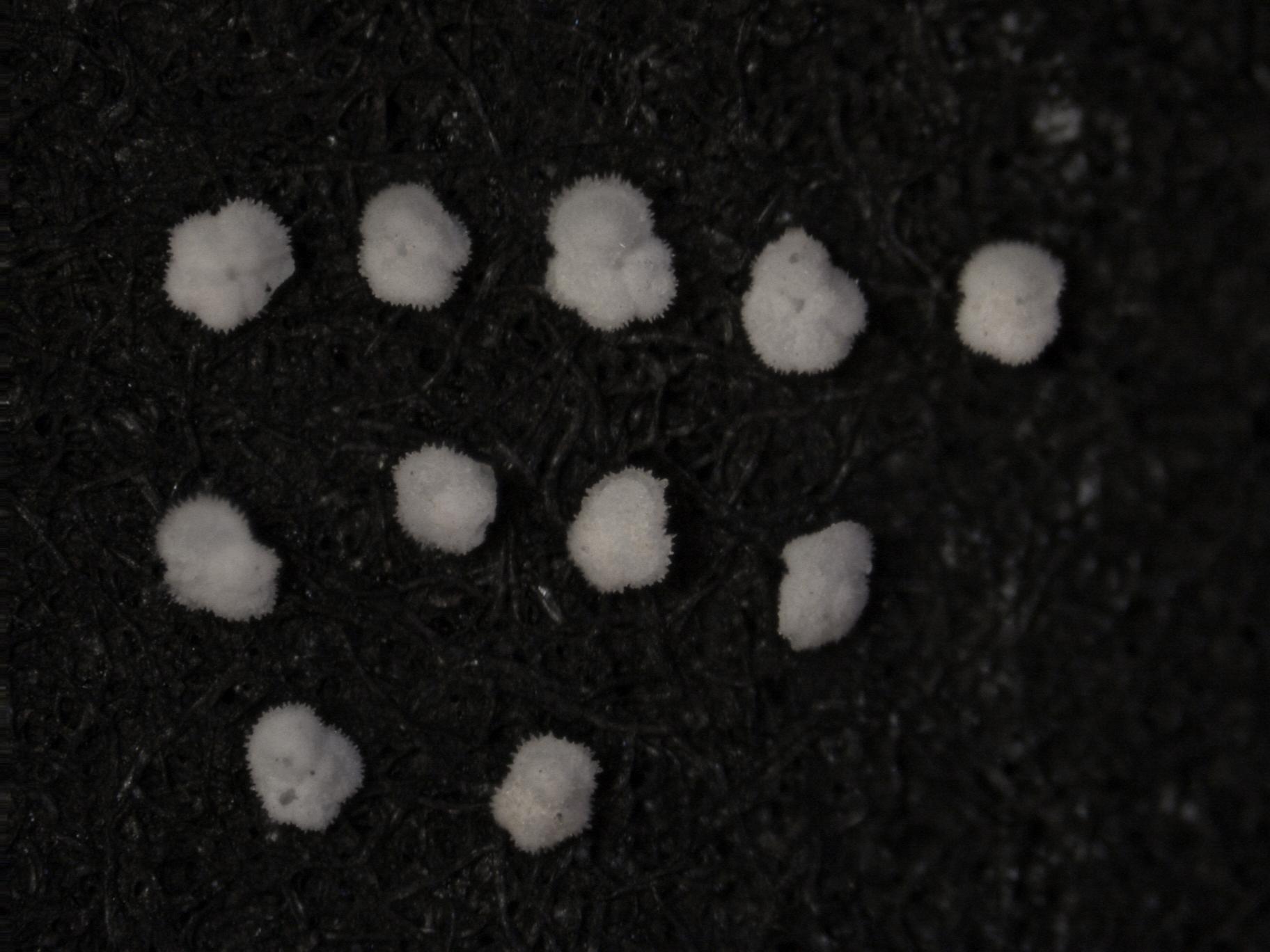

Supplement: S1 Data — (ZIP) [file pone.0267636.s001.zip › SDataImages/1209A-21H-3W_58-60_150_Mor2_5.0x_STACKED.jpg]

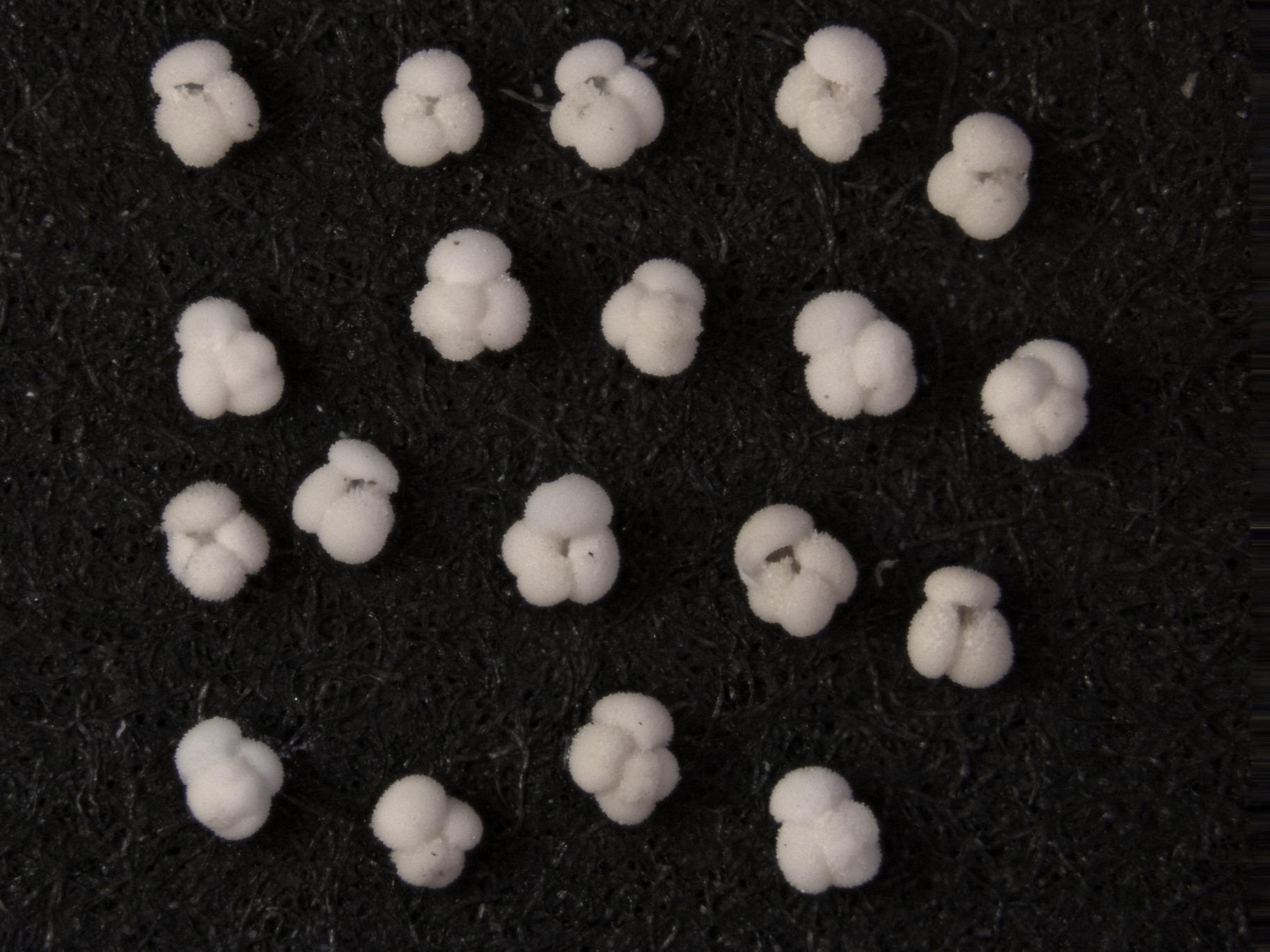

Supplement: S1 Data — (ZIP) [file pone.0267636.s001.zip › SDataImages/1209A-21H-3W_87-89_355_Aca1_2.5x_STACKED.jpg]

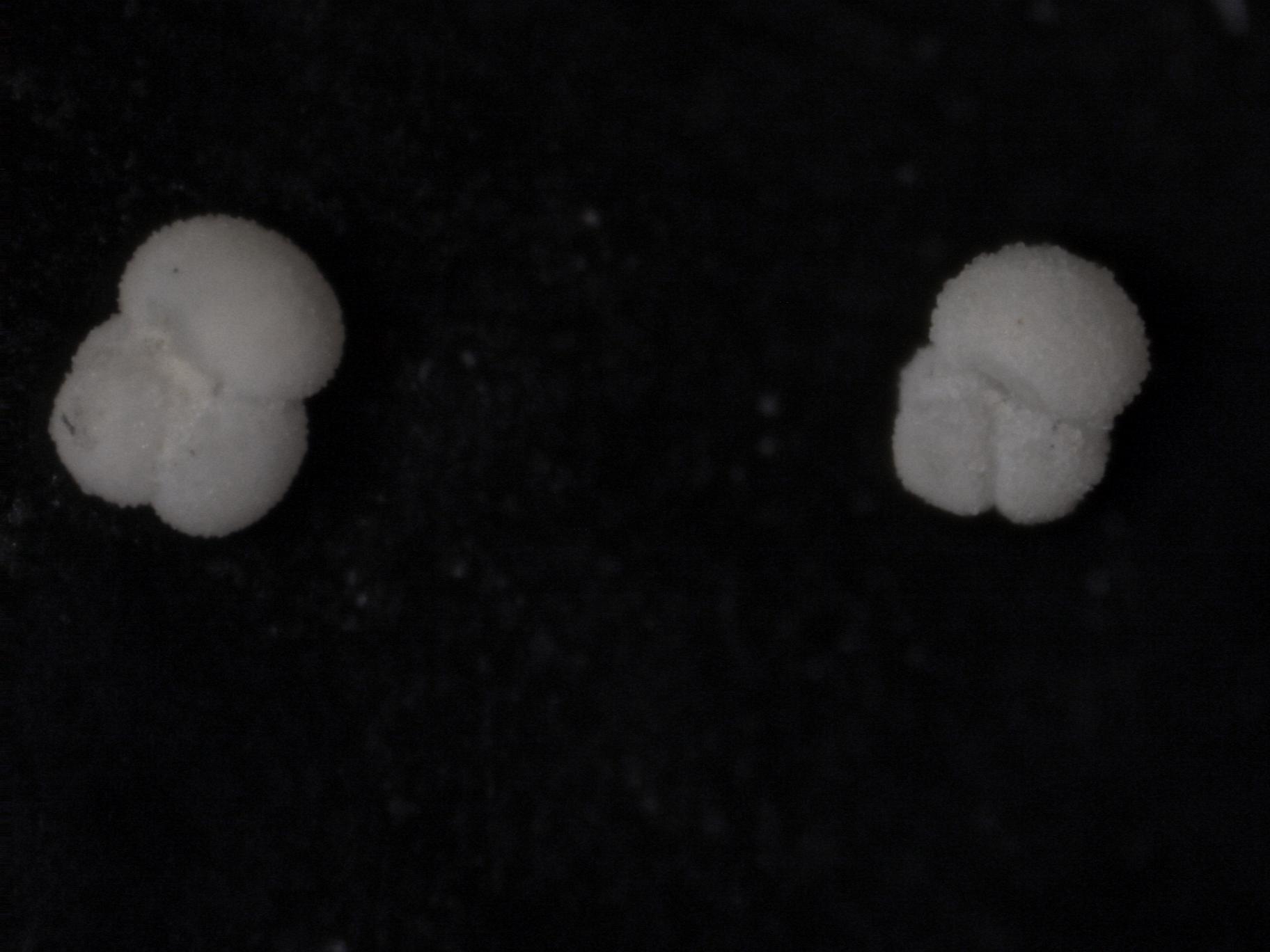

Supplement: S1 Data — (ZIP) [file pone.0267636.s001.zip › SDataImages/1209A-21H-3W_58-60_250_Sub1_8.0x_STACKED.jpg]

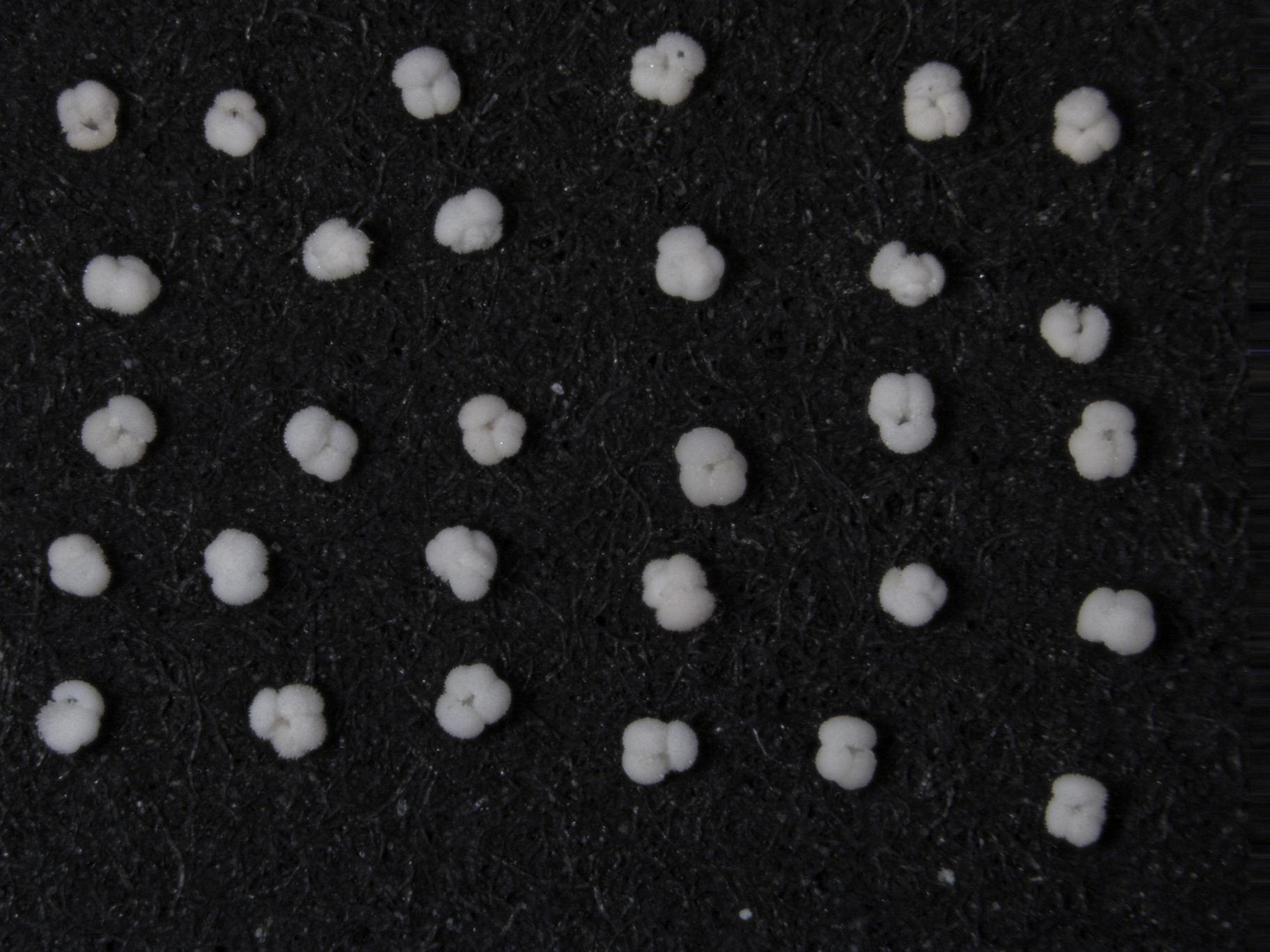

Supplement: S1 Data — (ZIP) [file pone.0267636.s001.zip › SDataImages/1209A-21H-3W_7-9_250_Aca1_2.0x_STACKED.jpg]

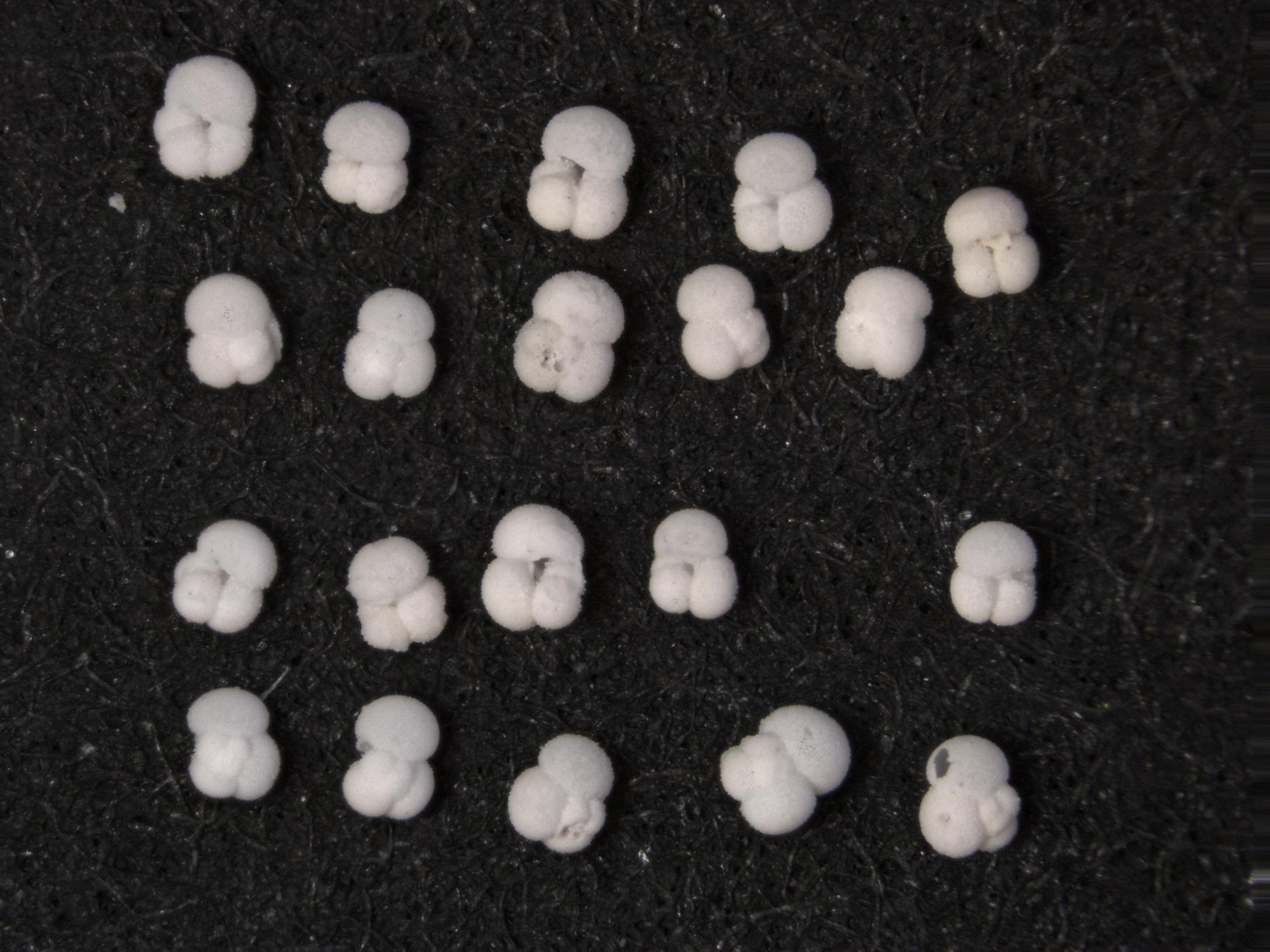

Supplement: S1 Data — (ZIP) [file pone.0267636.s001.zip › SDataImages/1209A-21H-3W_87-89_300_Sub1_2.5x_STACKED.jpg]

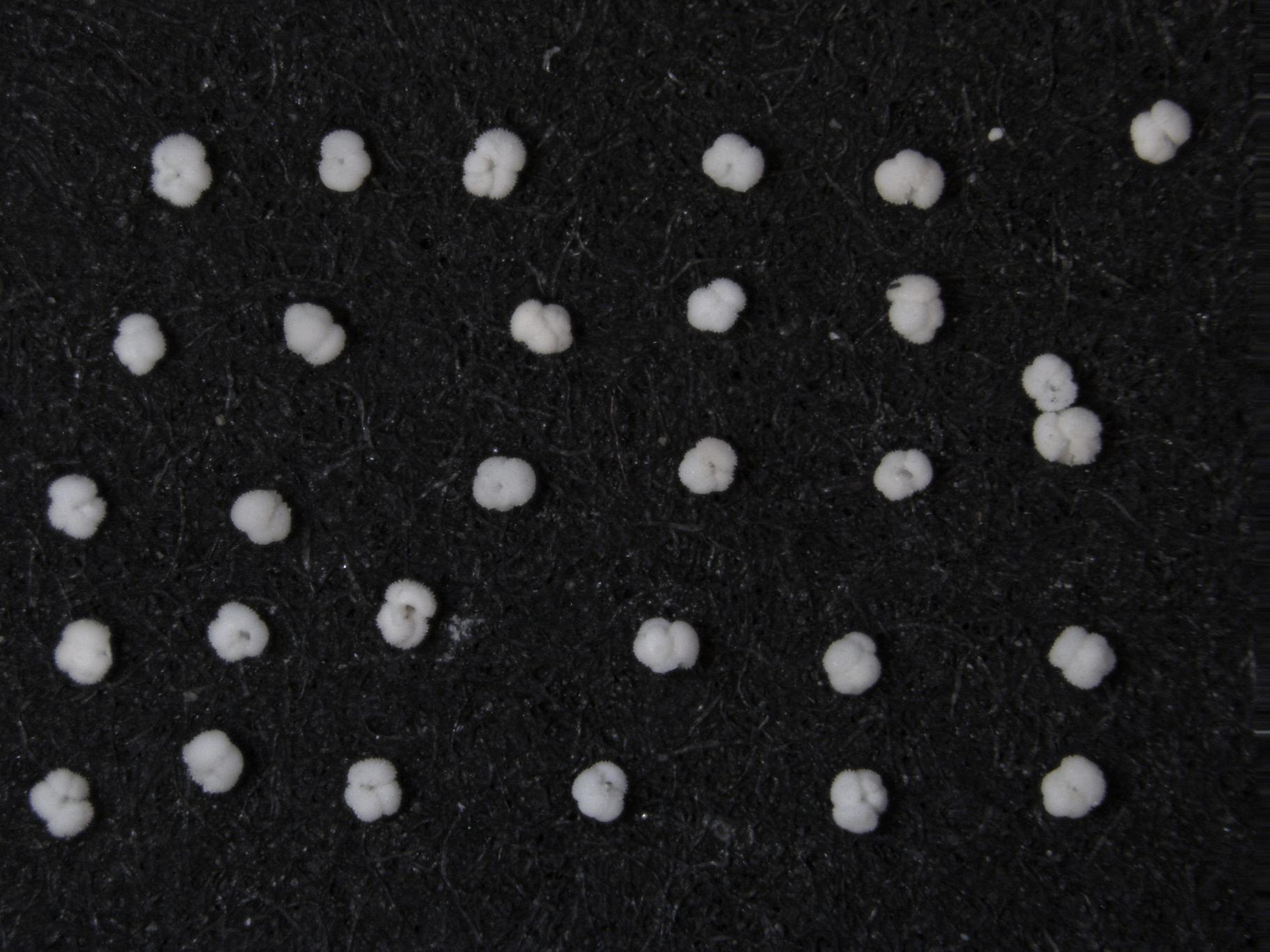

Supplement: S1 Data — (ZIP) [file pone.0267636.s001.zip › SDataImages/1209A-21H-3W_7-9_212_Aca1_2.0x_STACKED.jpg]

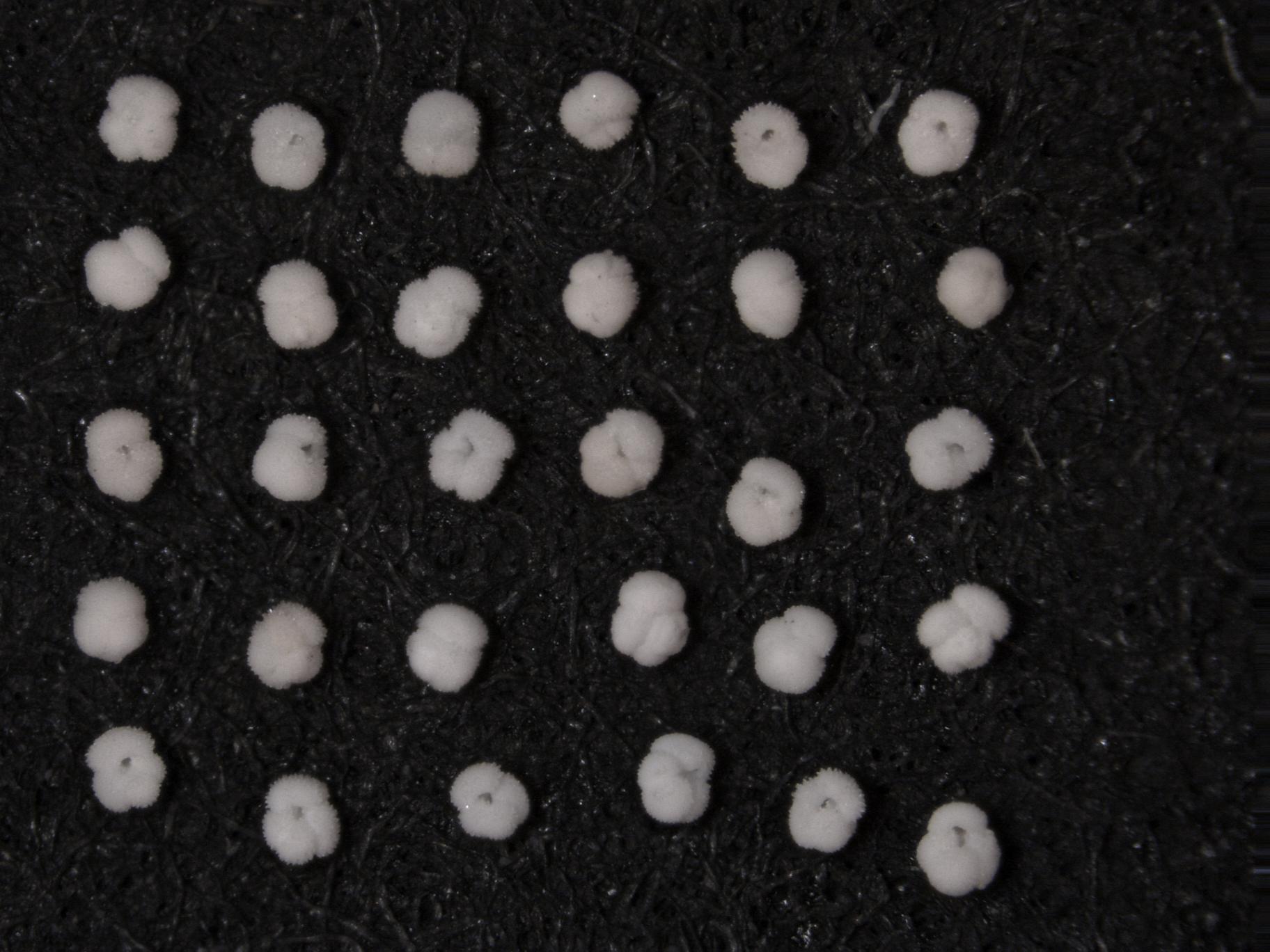

Supplement: S1 Data — (ZIP) [file pone.0267636.s001.zip › SDataImages/1209A-21H-3W_18-20_180_Aca1_3.2x_STACKED.jpg]

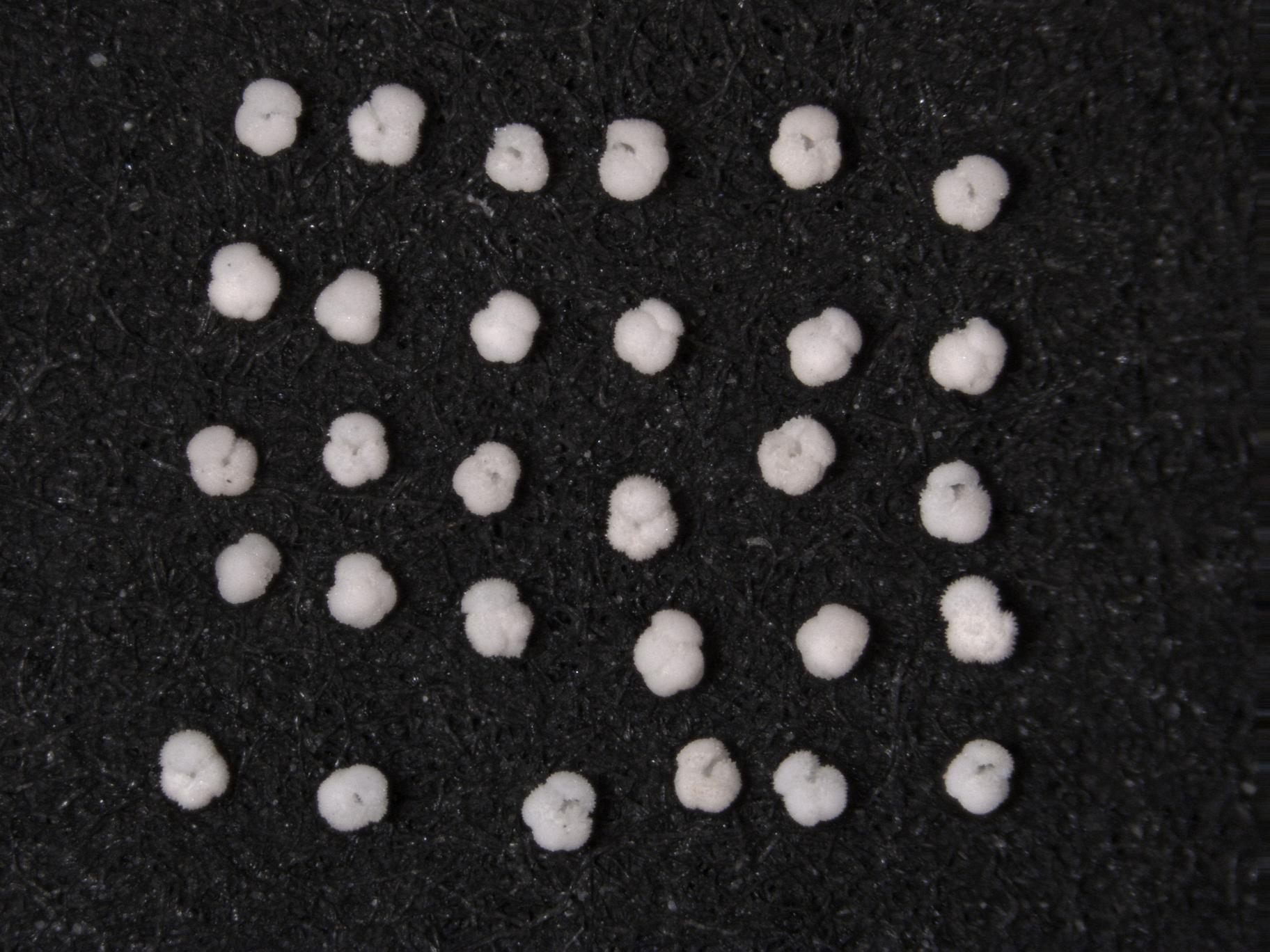

Supplement: S1 Data — (ZIP) [file pone.0267636.s001.zip › SDataImages/1209A-21H-3W_58-60_212_Aca1_2.5x_STACKED.jpg]

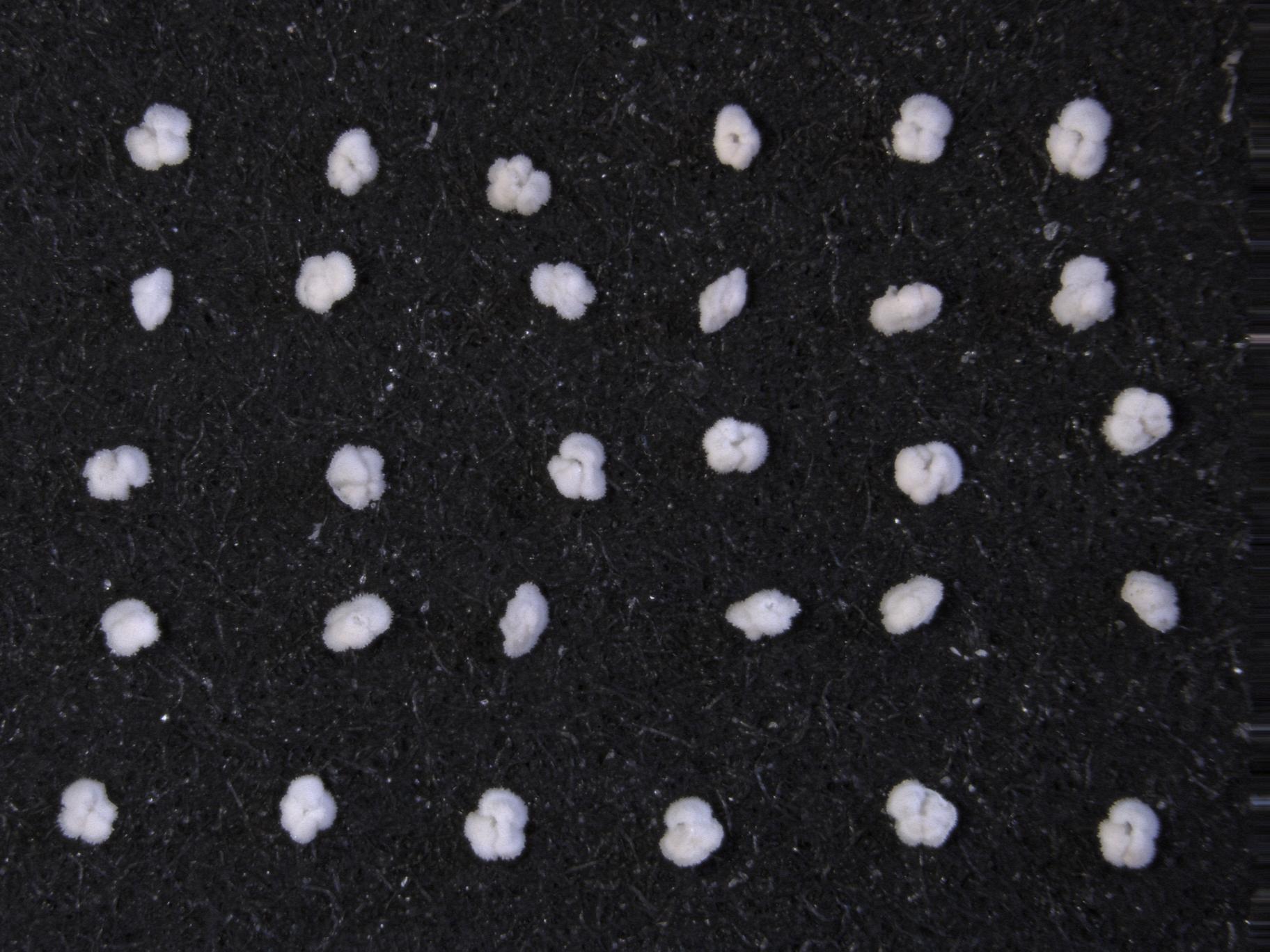

Supplement: S1 Data — (ZIP) [file pone.0267636.s001.zip › SDataImages/1209A-21H-2W_86-88_250_Mor1_1.6x_STACKED.jpg]

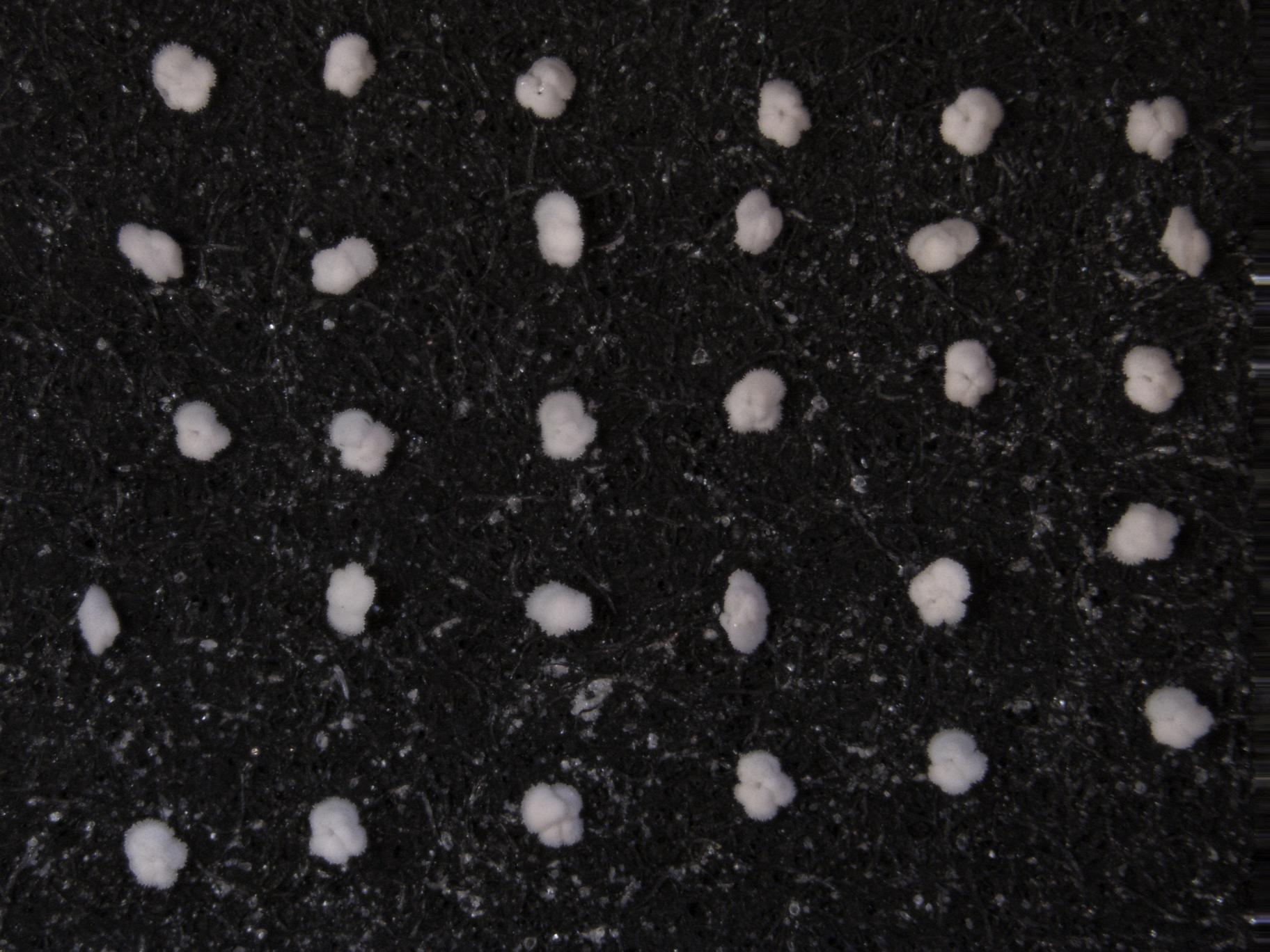

Supplement: S1 Data — (ZIP) [file pone.0267636.s001.zip › SDataImages/1209A-21H-2W_86-88_180_Mor1_2.0x_STACKED.jpg]

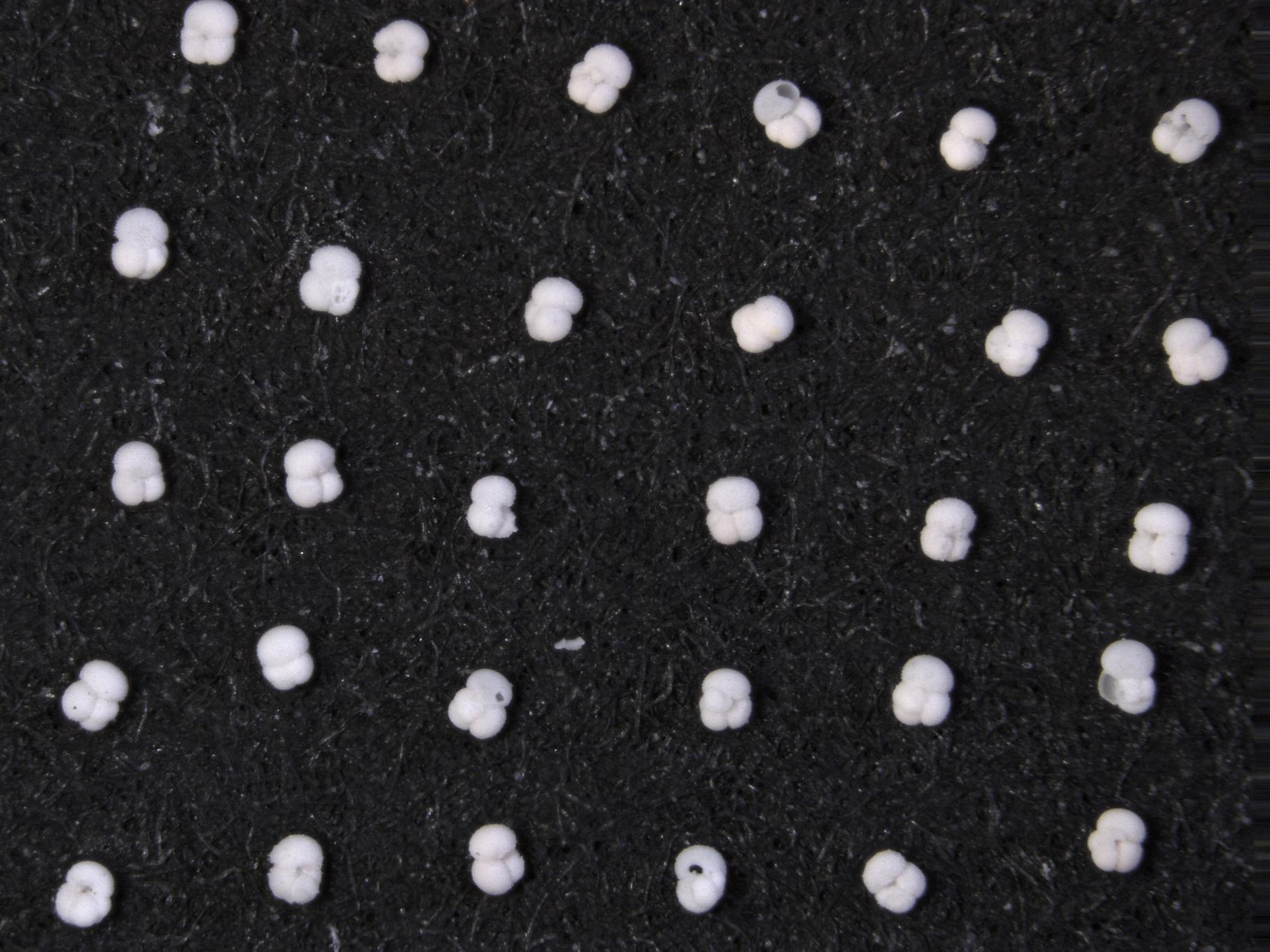

Supplement: S1 Data — (ZIP) [file pone.0267636.s001.zip › SDataImages/1209A-21H-3W_68-70_212_Sub1_2.0x_STACKED.jpg]

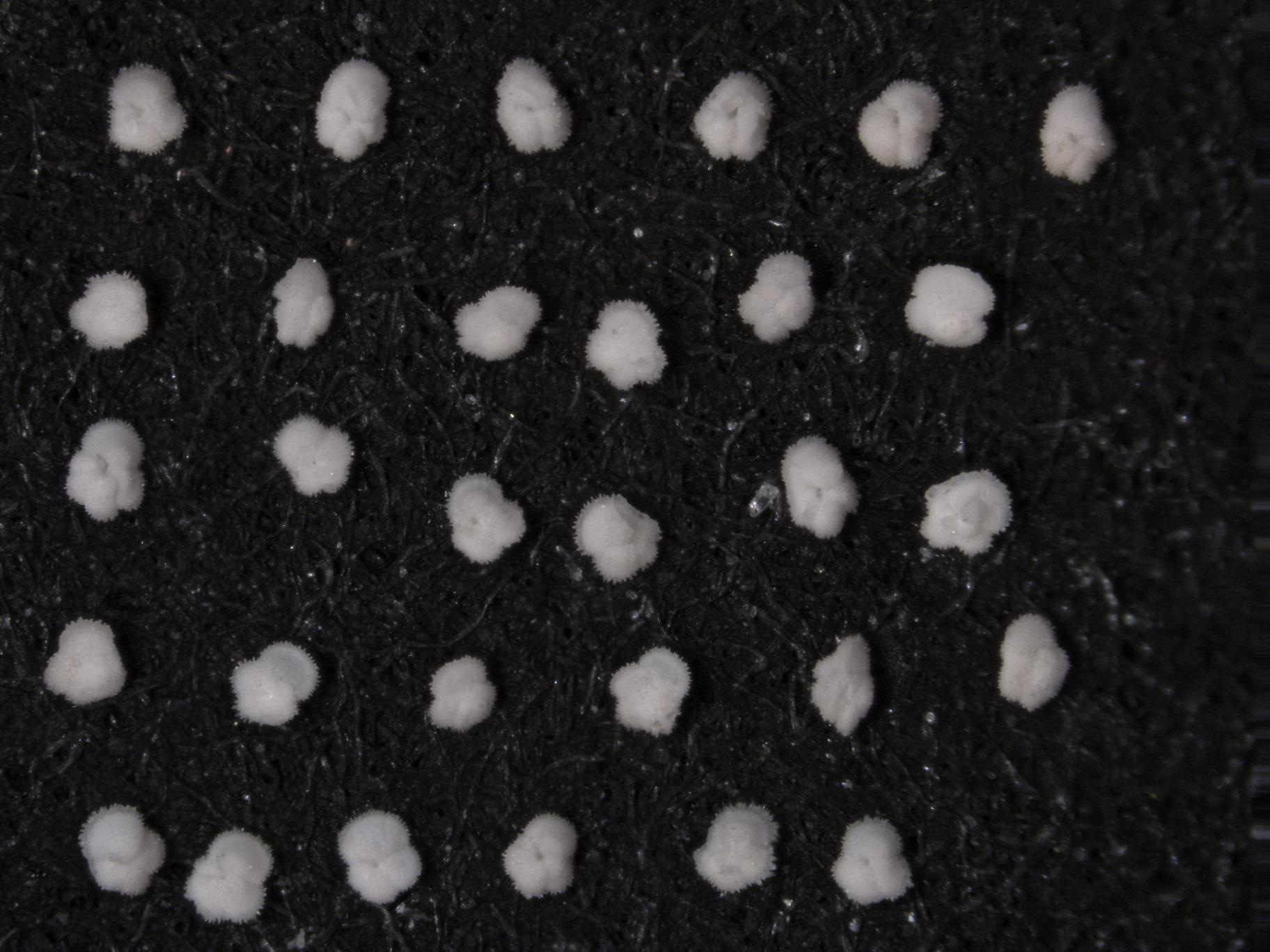

Supplement: S1 Data — (ZIP) [file pone.0267636.s001.zip › SDataImages/1209A-21H-2W_146-148_150_Mor1_3.2x_STACKED.jpg]

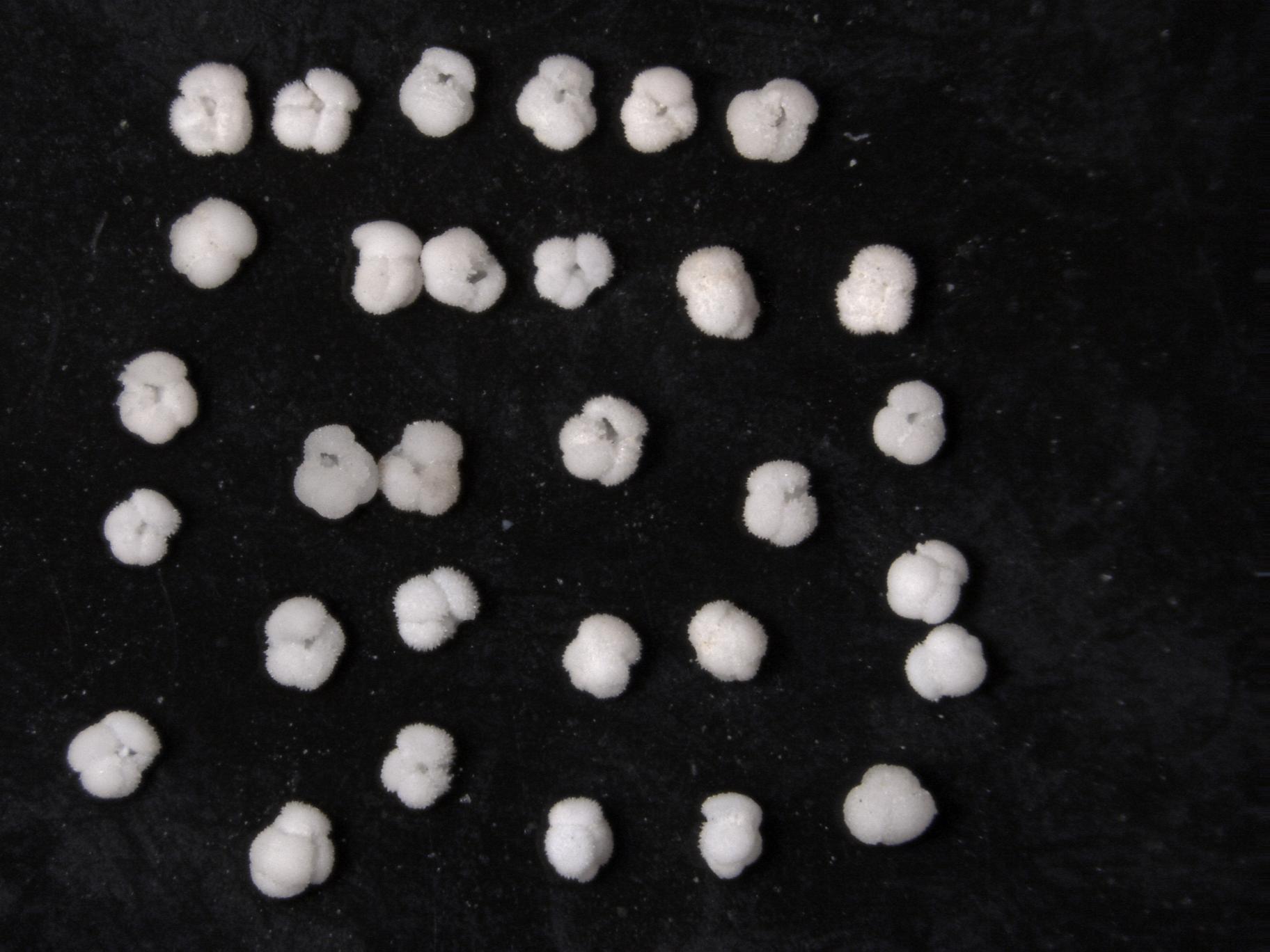

Supplement: S1 Data — (ZIP) [file pone.0267636.s001.zip › SDataImages/1209A-21H-3W_58-60_250_Aca1_5.0x_STACKED.jpg]

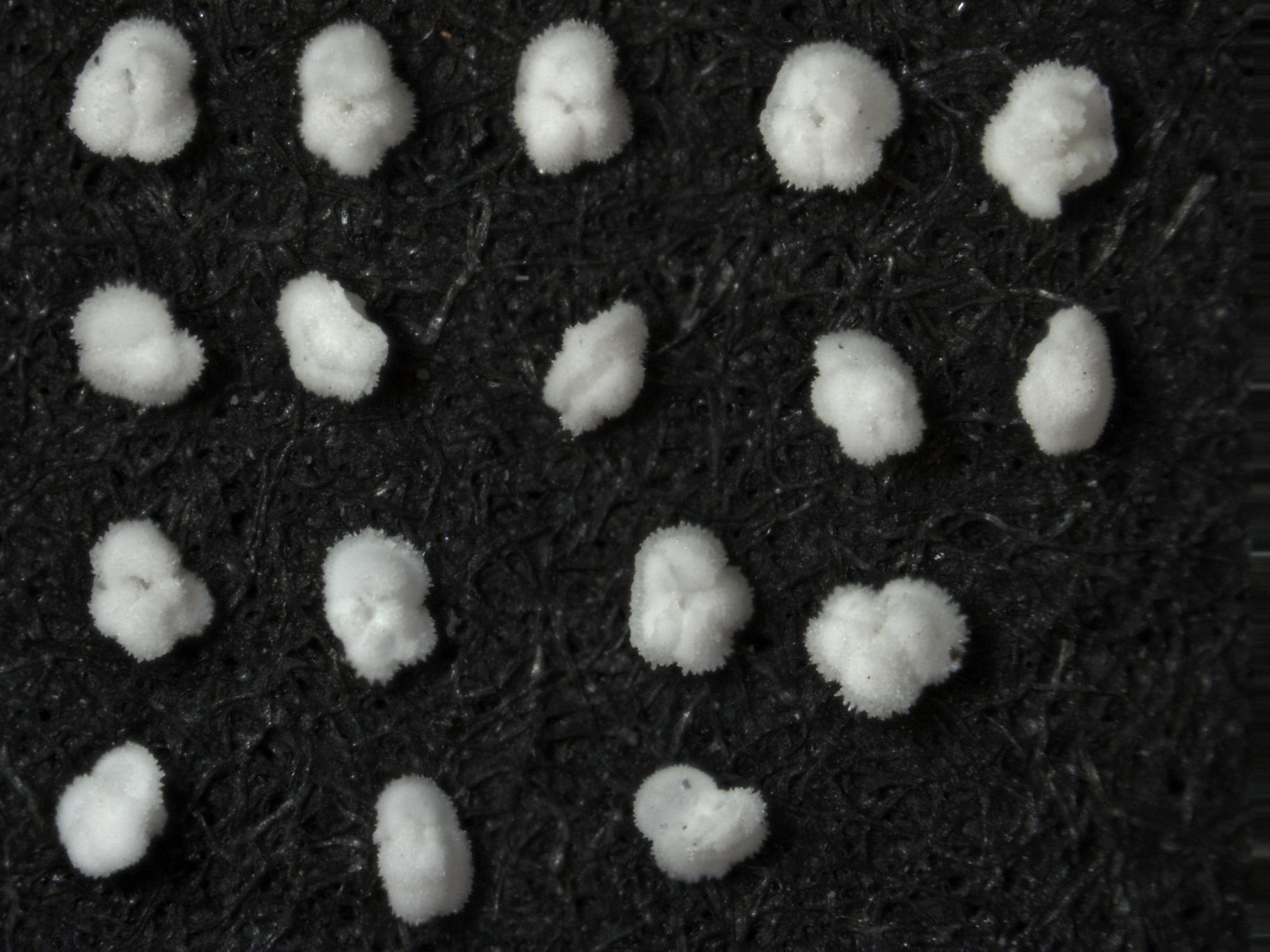

Supplement: S1 Data — (ZIP) [file pone.0267636.s001.zip › SDataImages/1209A-21H-3W_38-40_212_Mor2_4.0x_STACKED.jpg]

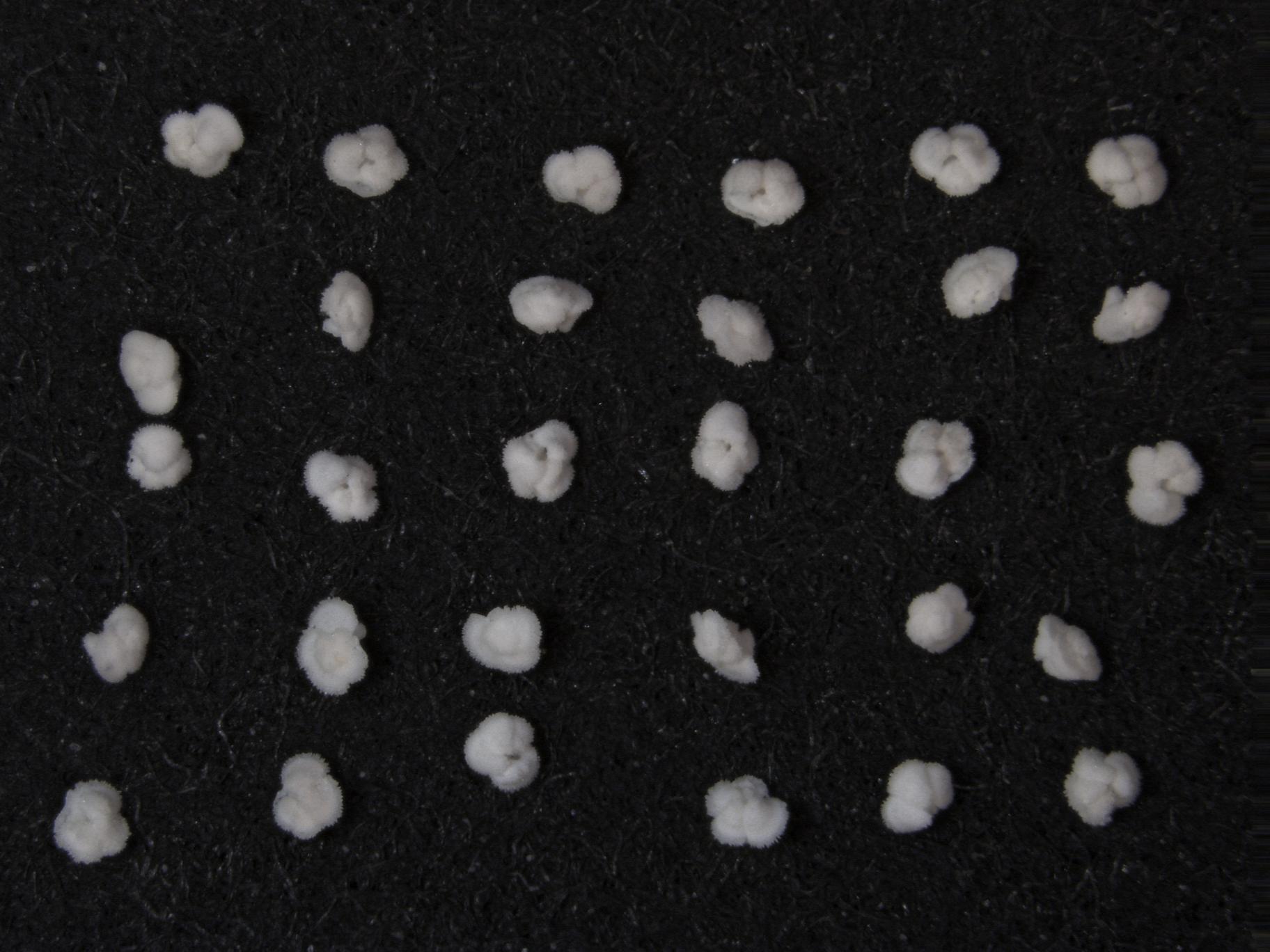

Supplement: S1 Data — (ZIP) [file pone.0267636.s001.zip › SDataImages/1209A-21H-3W_77-79_300_Mor1_1.6x_STACKED.jpg]

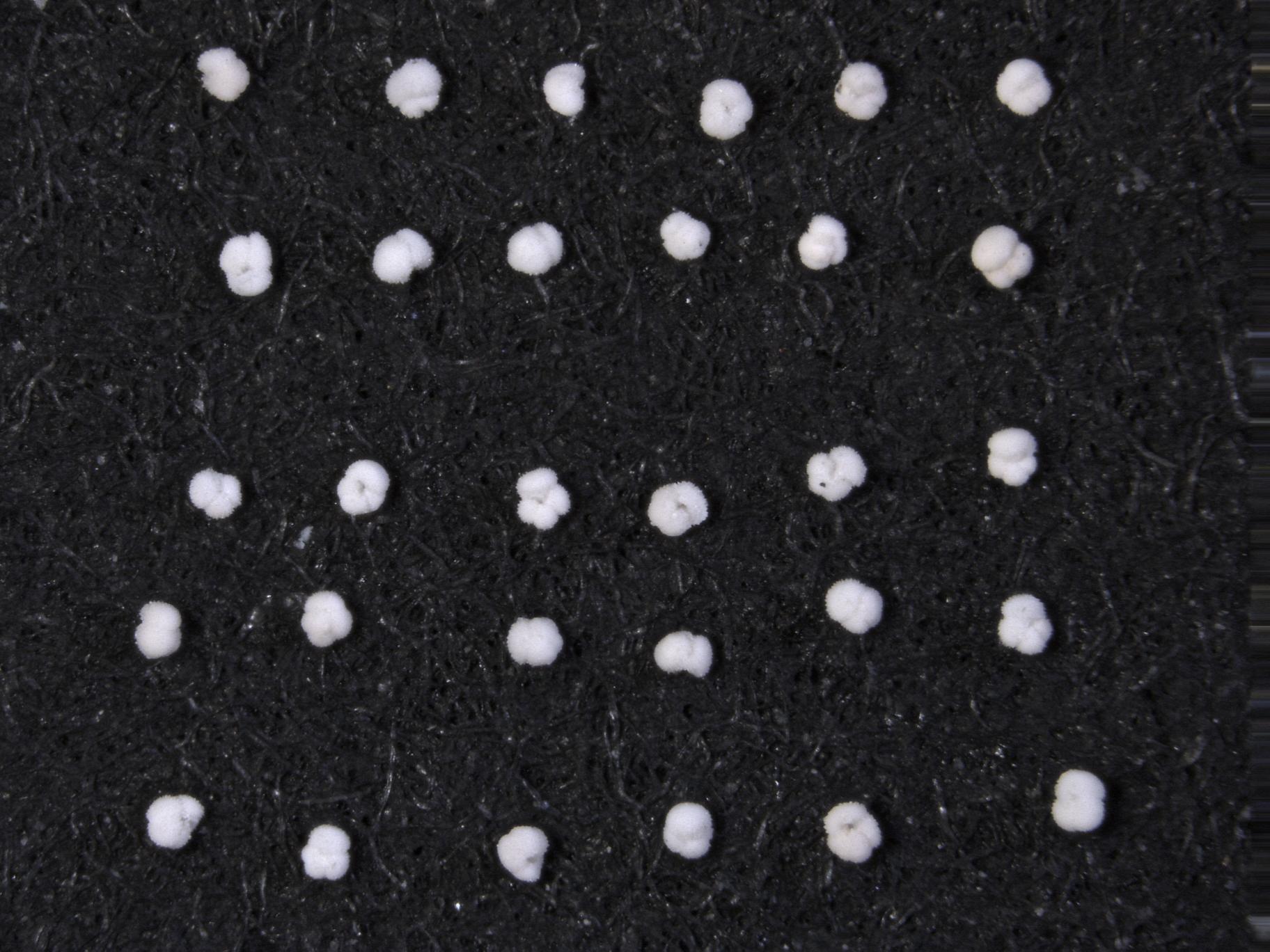

Supplement: S1 Data — (ZIP) [file pone.0267636.s001.zip › SDataImages/1209A-21H-3W_68-70_150_Aca1_2.5x_STACKED.jpg]

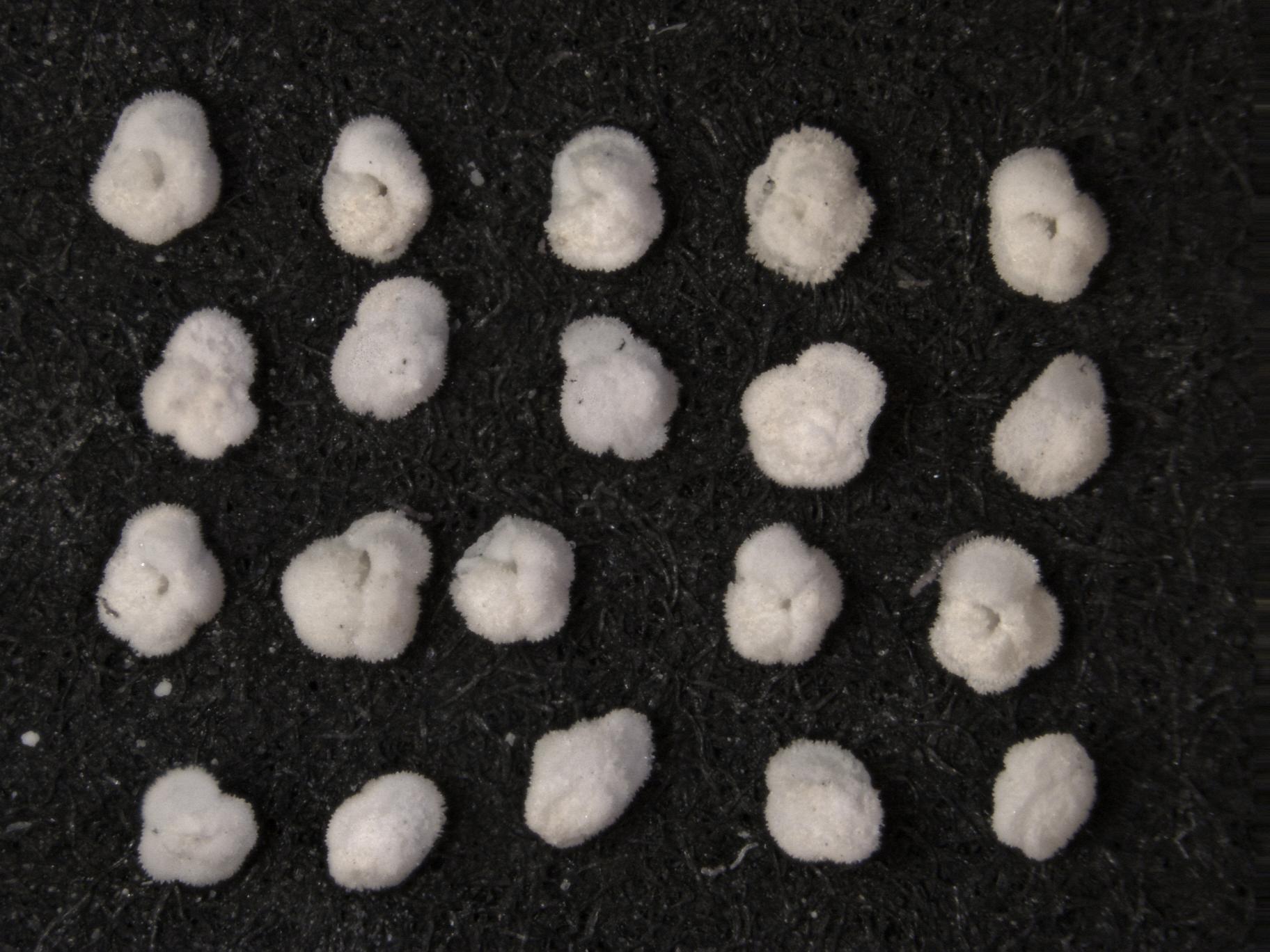

Supplement: S1 Data — (ZIP) [file pone.0267636.s001.zip › SDataImages/1209A-21H-3W_58-60_355_Mor2_2.5x_STACKED.jpg]

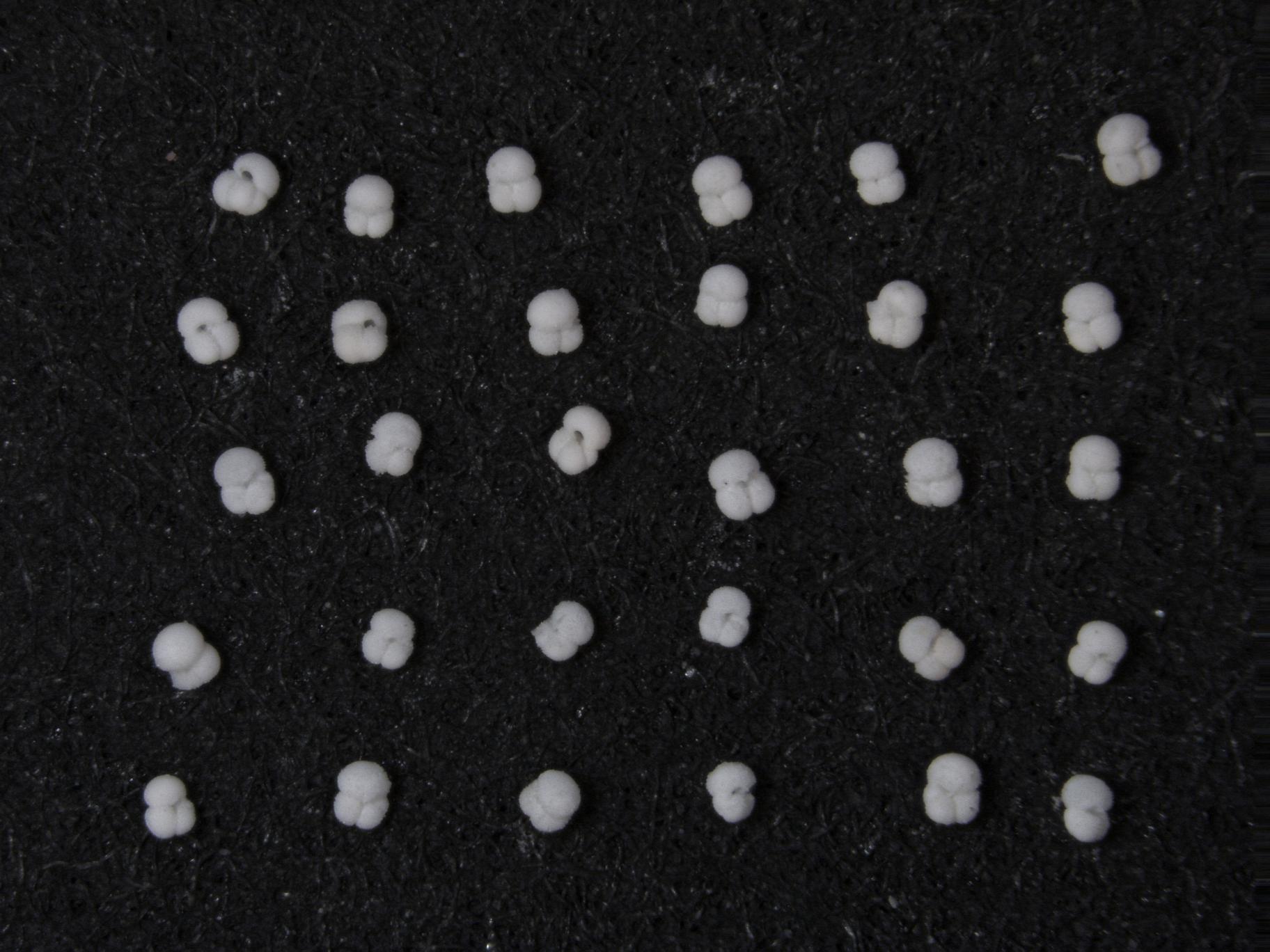

Supplement: S1 Data — (ZIP) [file pone.0267636.s001.zip › SDataImages/1209A-21H-3W_47-49_212_Sub1_2.0x_STACKED.jpg]

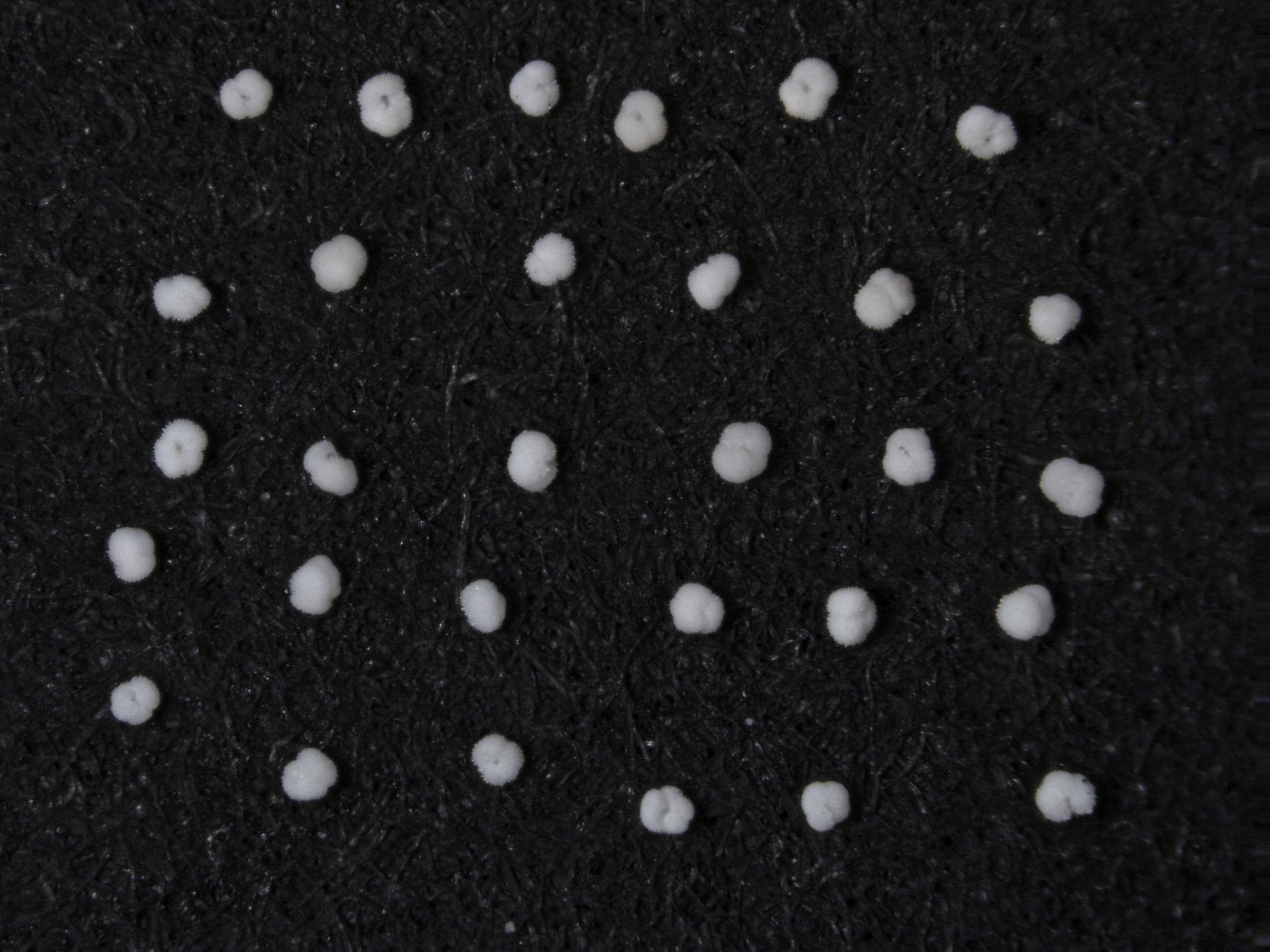

Supplement: S1 Data — (ZIP) [file pone.0267636.s001.zip › SDataImages/1209A-21H-3W_47-49_150_Aca1_2.5x_STACKED.jpg]

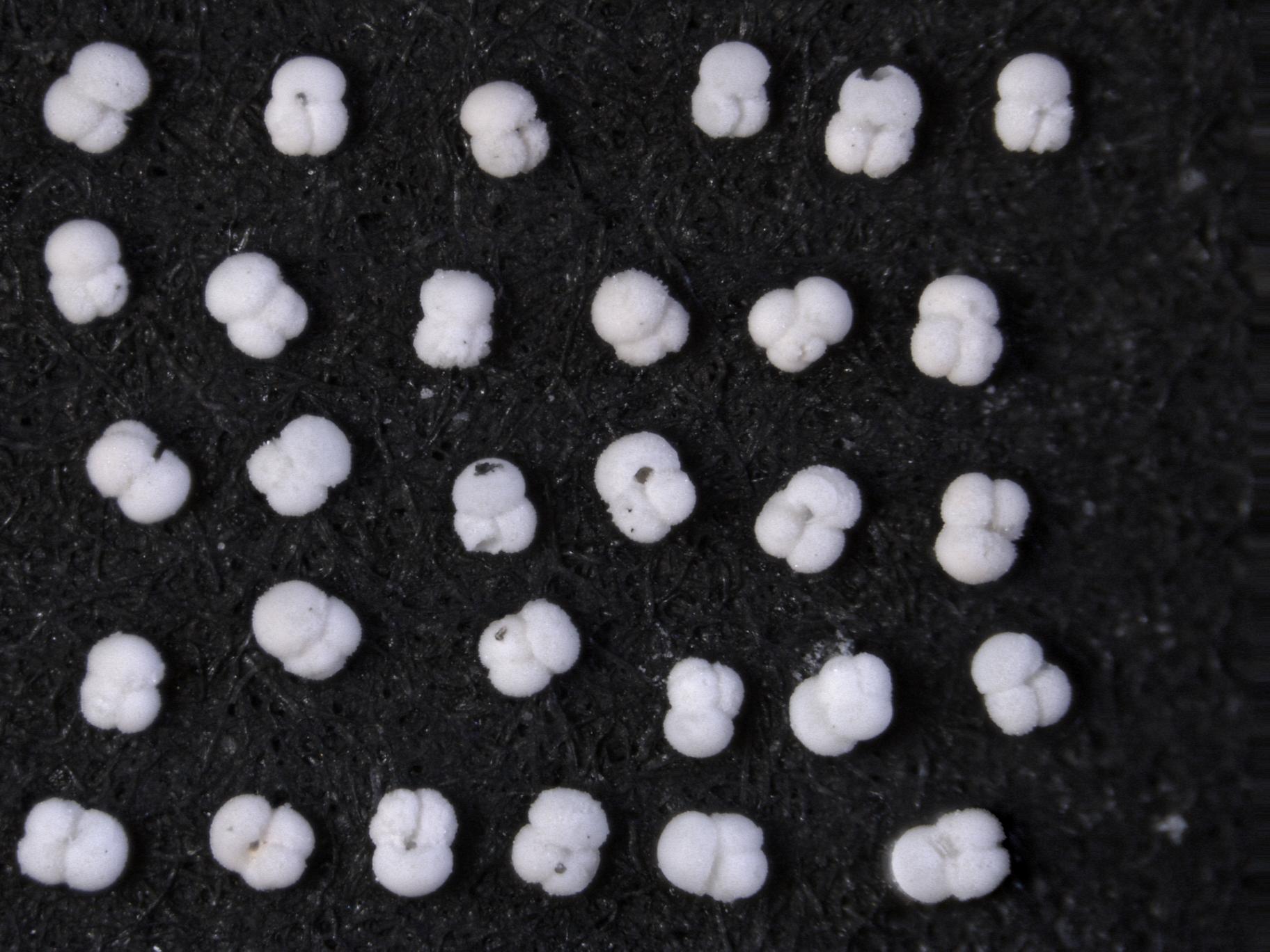

Supplement: S1 Data — (ZIP) [file pone.0267636.s001.zip › SDataImages/1209A-21H-3W_18-20_212_Sub1_3.2x_STACKED.jpg]

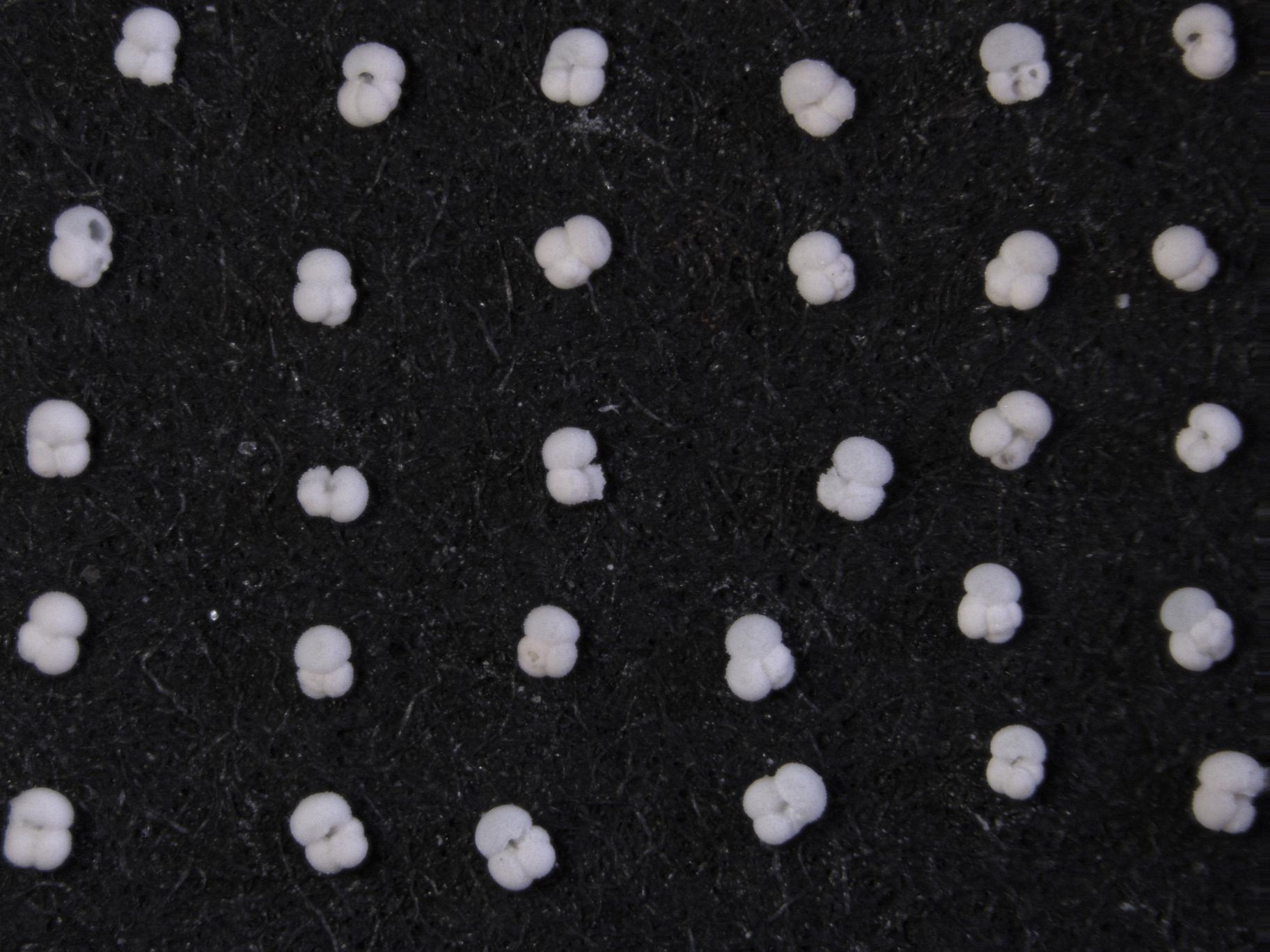

Supplement: S1 Data — (ZIP) [file pone.0267636.s001.zip › SDataImages/1209A-21H-3W_68-70_250_Sub1_2.0x_STACKED.jpg]

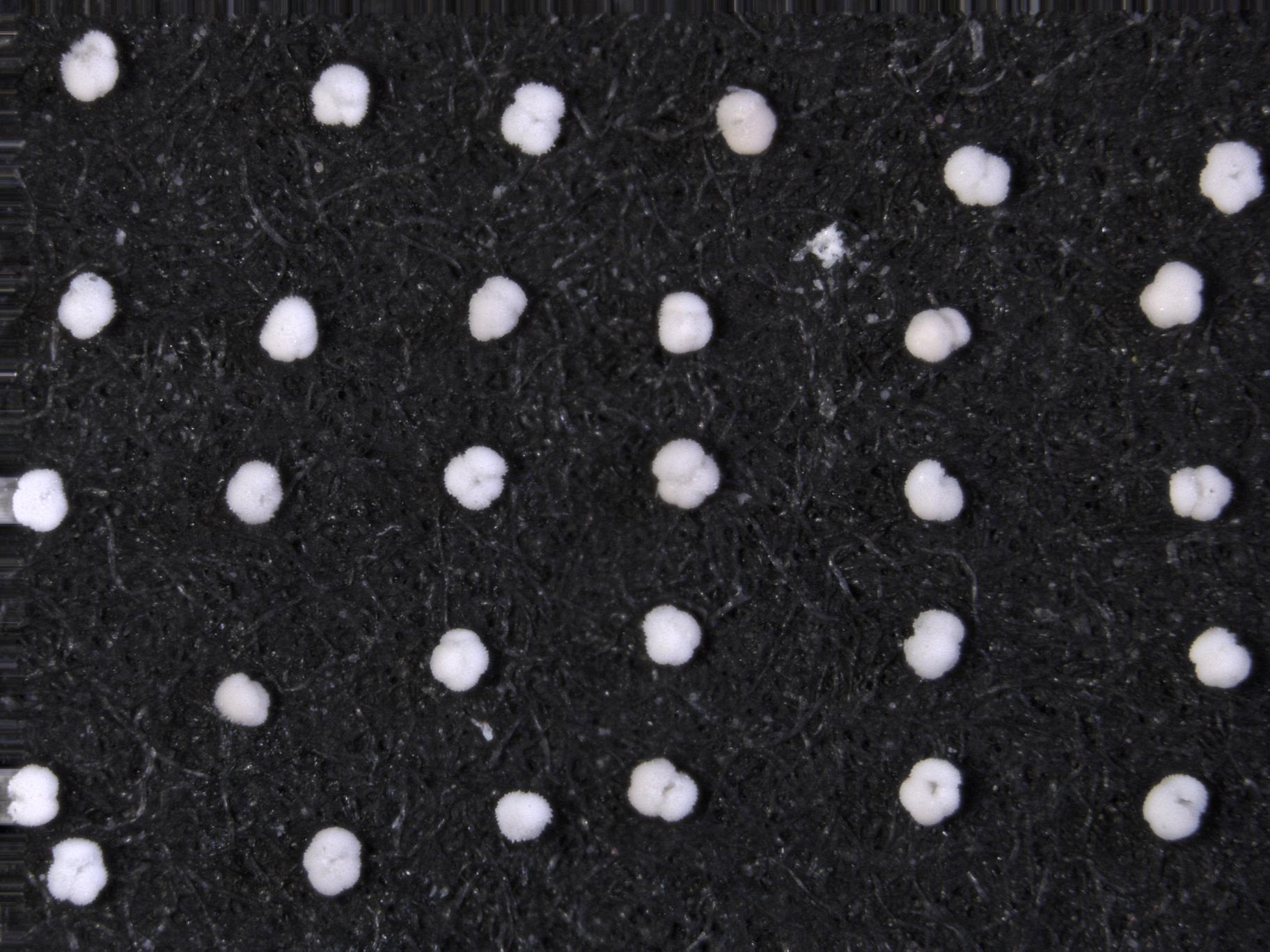

Supplement: S1 Data — (ZIP) [file pone.0267636.s001.zip › SDataImages/1209A-21H-3W_68-70_180_Aca1_2.5x_STACKED.jpg]

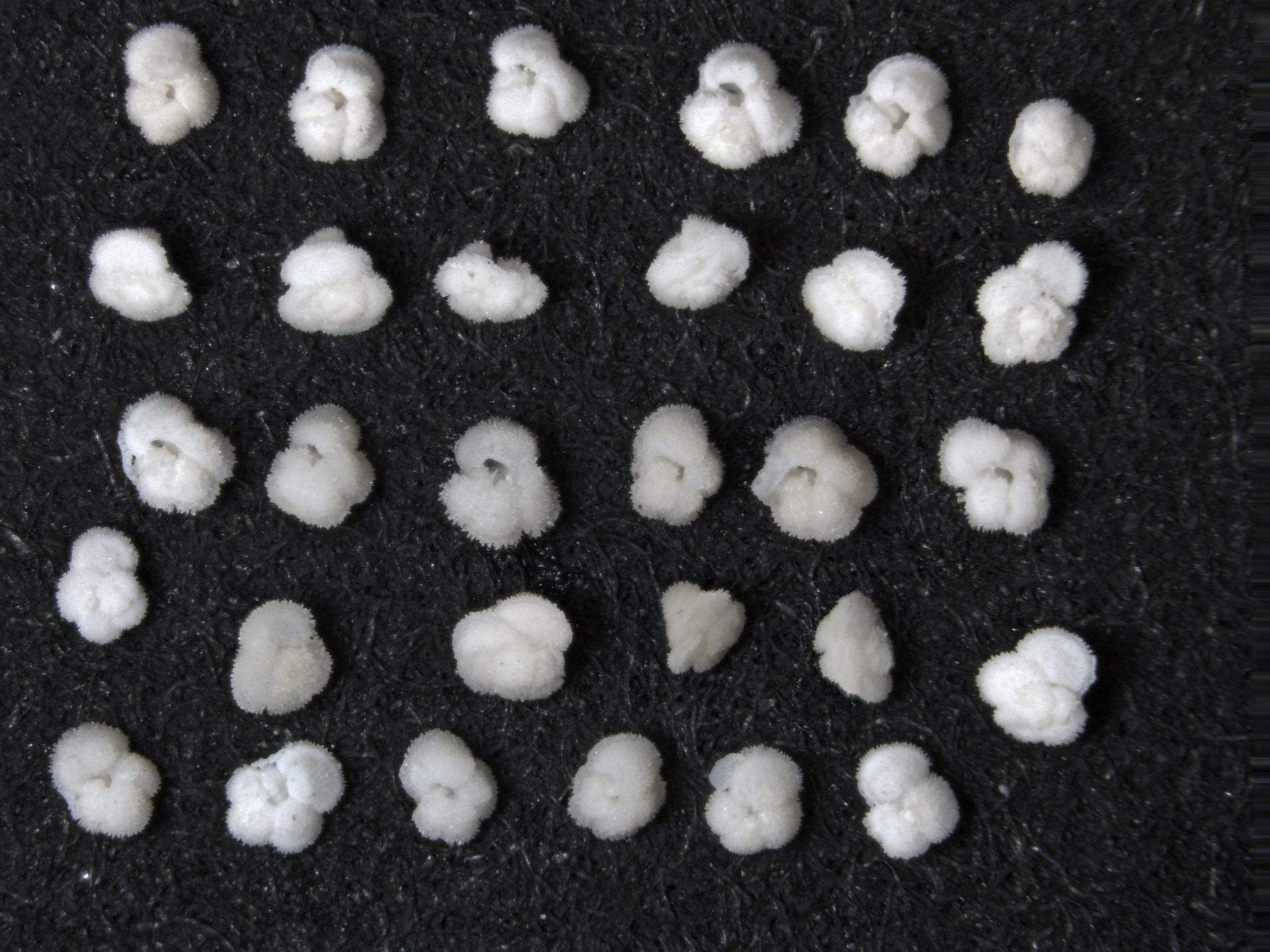

Supplement: S1 Data — (ZIP) [file pone.0267636.s001.zip › SDataImages/1209A-21H-3W_117-119_355_Mor1_2.0x_STACKED.jpg]

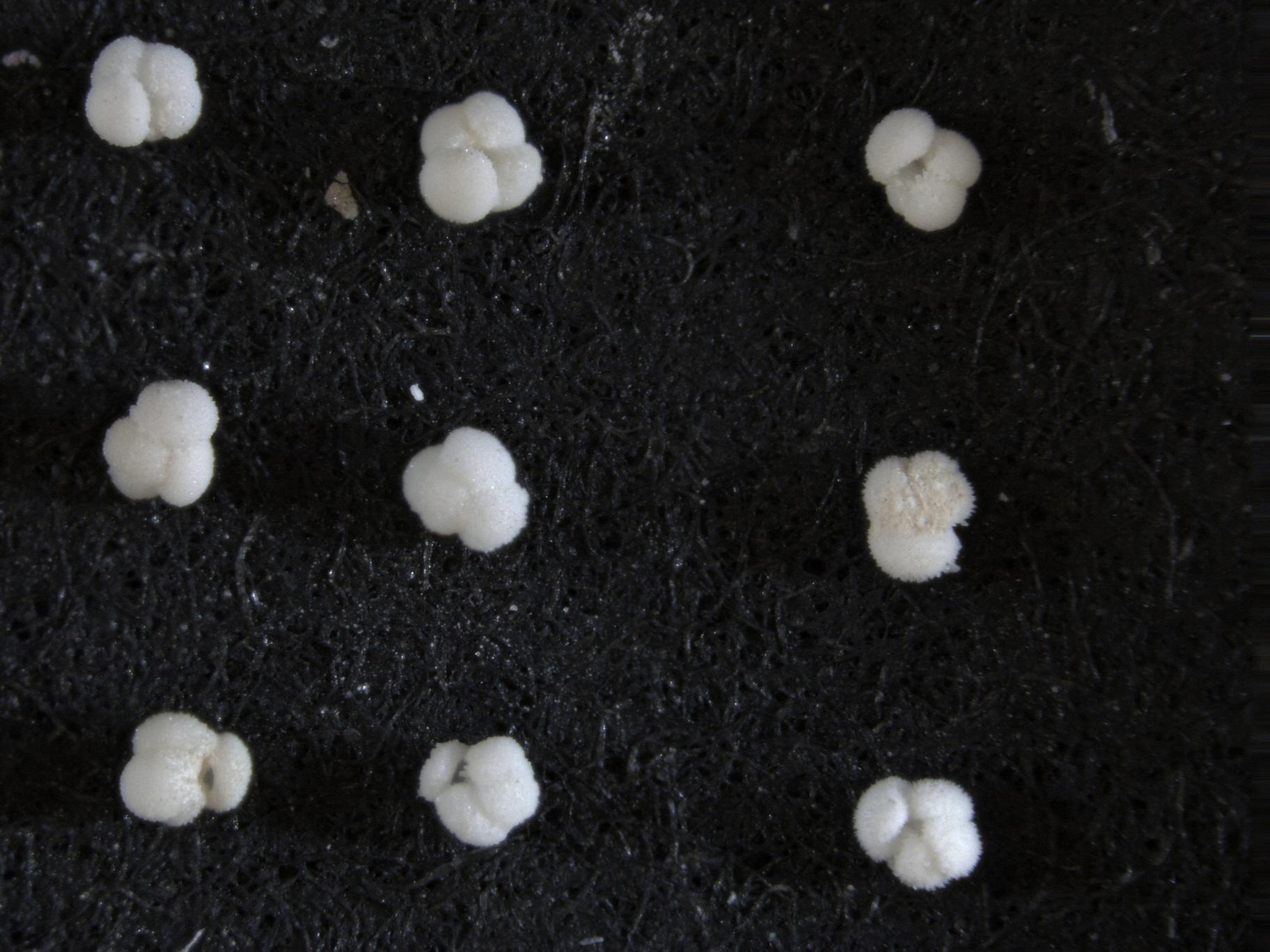

Supplement: S1 Data — (ZIP) [file pone.0267636.s001.zip › SDataImages/1209A-21H-3W_58-60_355_Aca3_2.5x_STACKED.jpg]

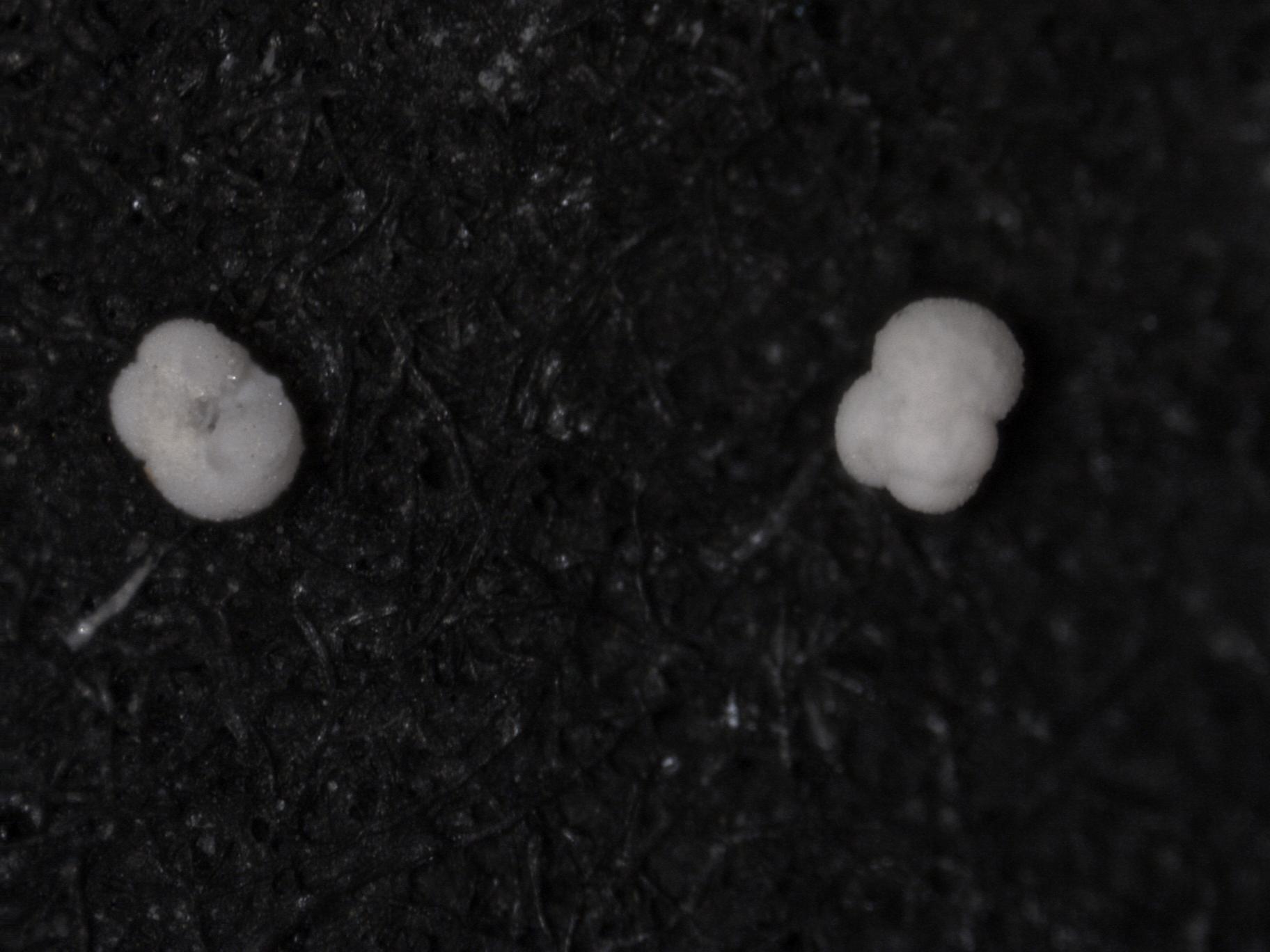

Supplement: S1 Data — (ZIP) [file pone.0267636.s001.zip › SDataImages/1209A-21H-3W_58-60_212_Sub1_6.3x_STACKED.jpg]

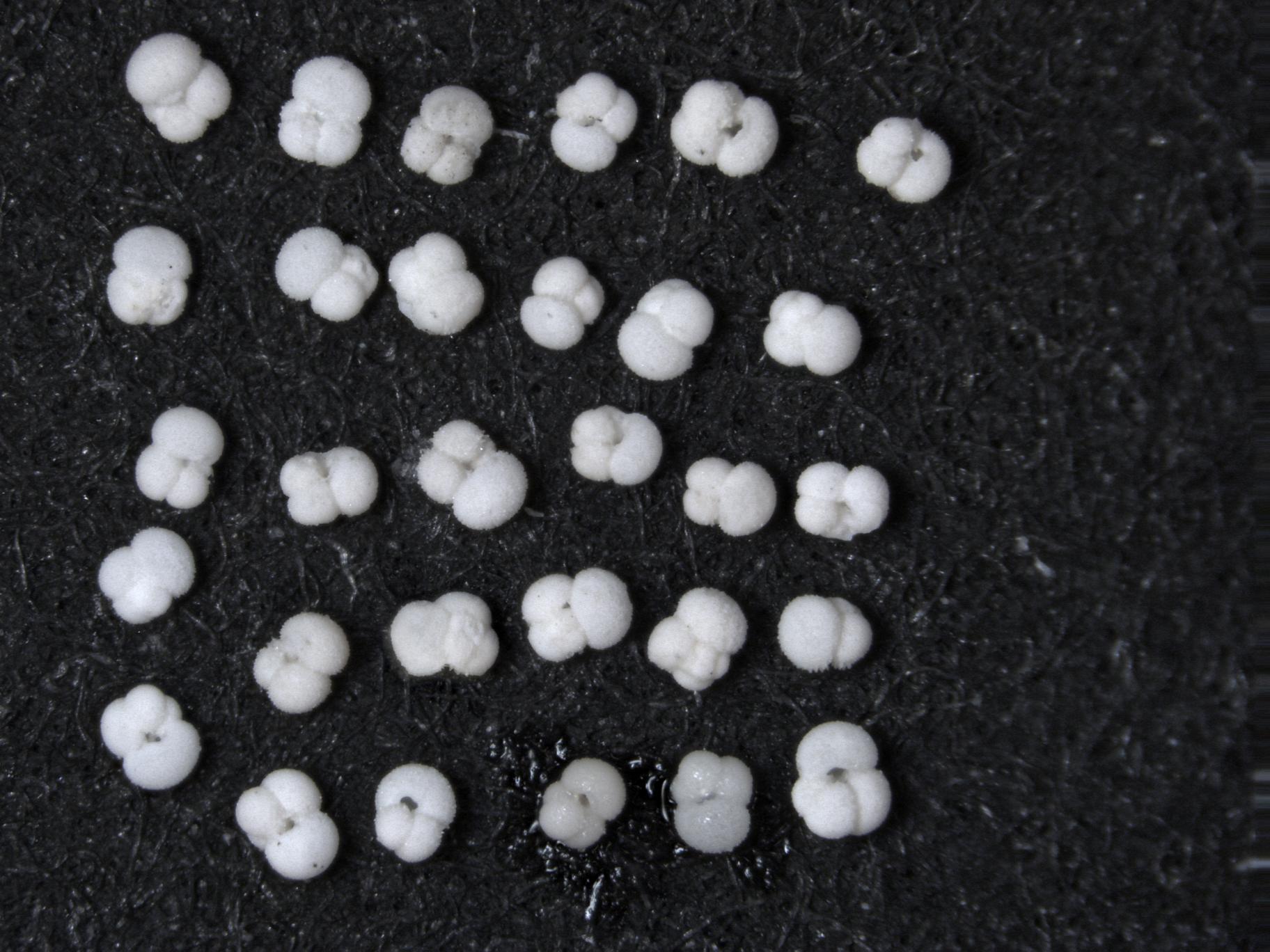

Supplement: S1 Data — (ZIP) [file pone.0267636.s001.zip › SDataImages/1209A-21H-3W_87-89_250_Sub1_2.5x_STACKED.jpg]

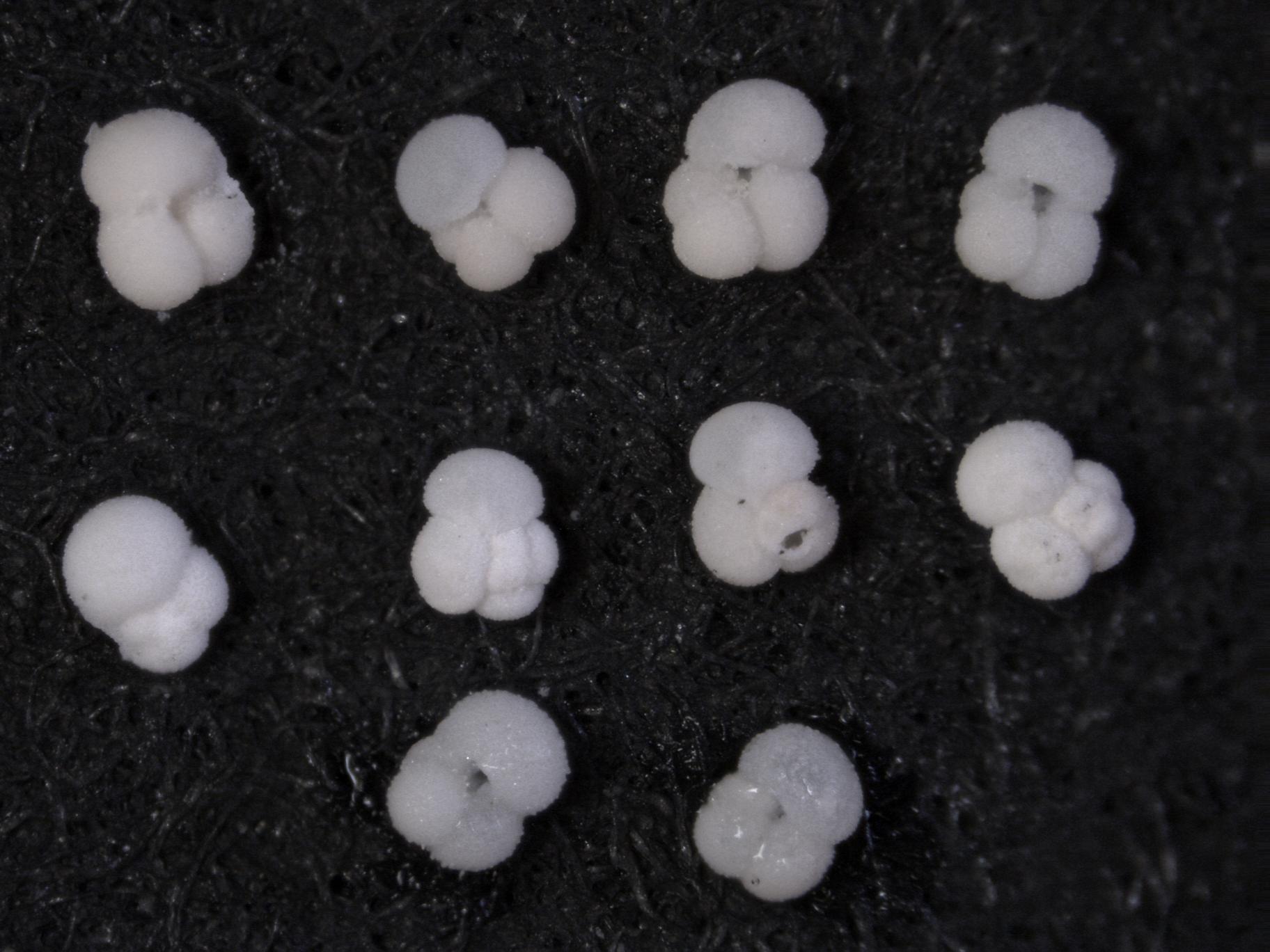

Supplement: S1 Data — (ZIP) [file pone.0267636.s001.zip › SDataImages/1209A-21H-3W_18-20_300_Sub1_4.0x_STACKED.jpg]

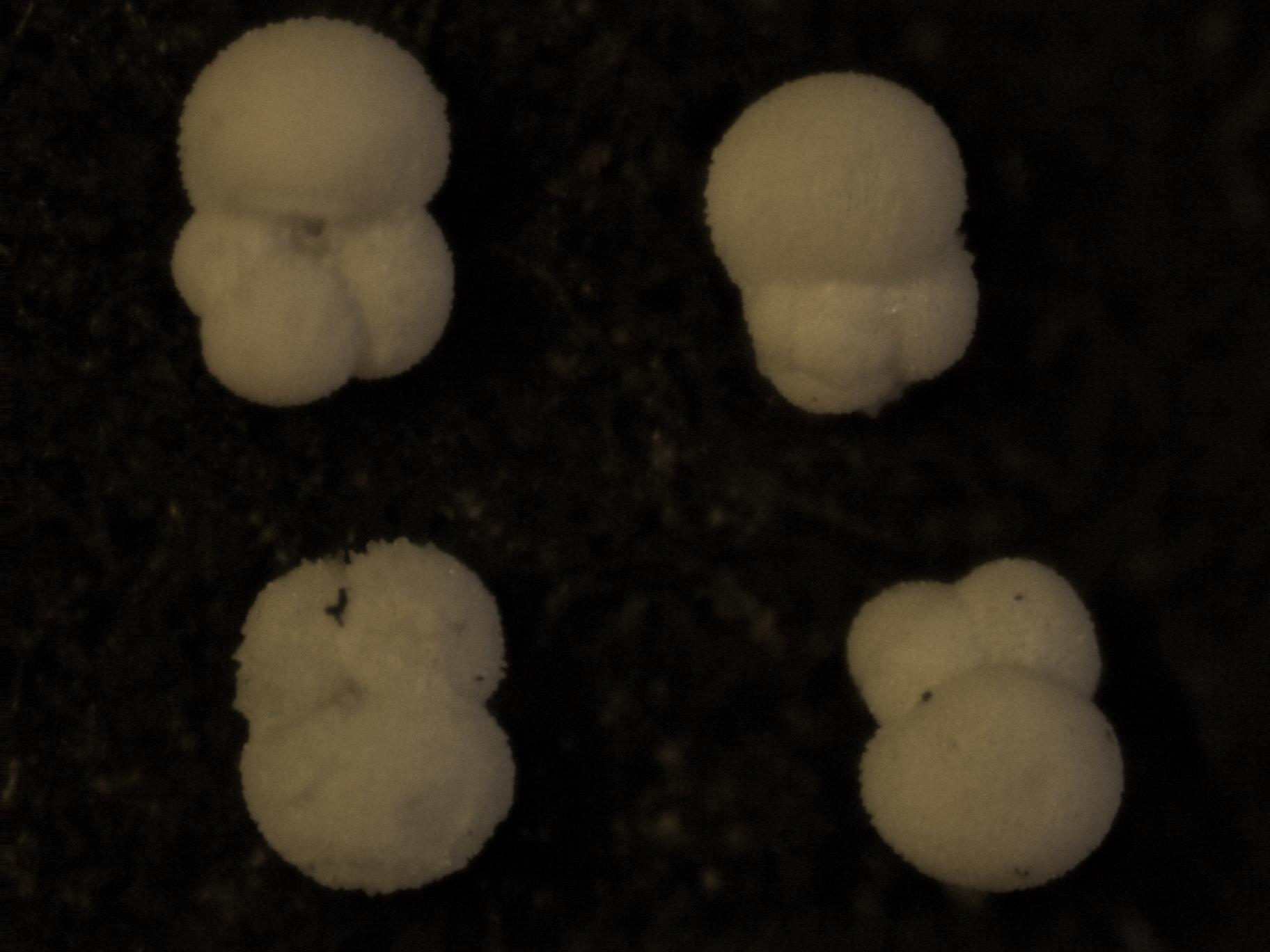

Supplement: S1 Data — (ZIP) [file pone.0267636.s001.zip › SDataImages/1209A-21H-3W_58-60_300_Sub1_8.0x_STACKED.jpg]

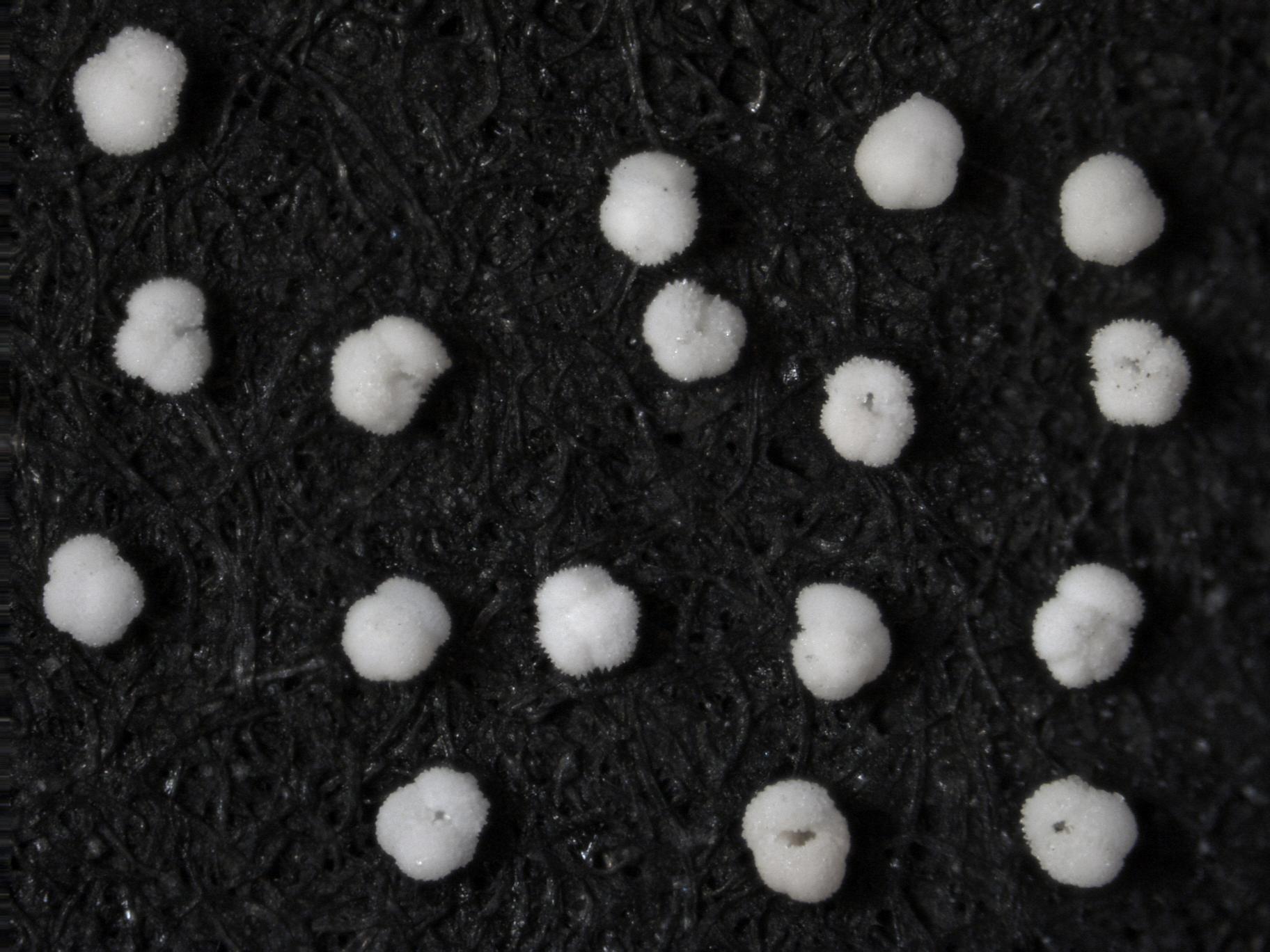

Supplement: S1 Data — (ZIP) [file pone.0267636.s001.zip › SDataImages/1209A-21H-3W_38-40_150_Aca2_5.0x_STACKED.jpg]

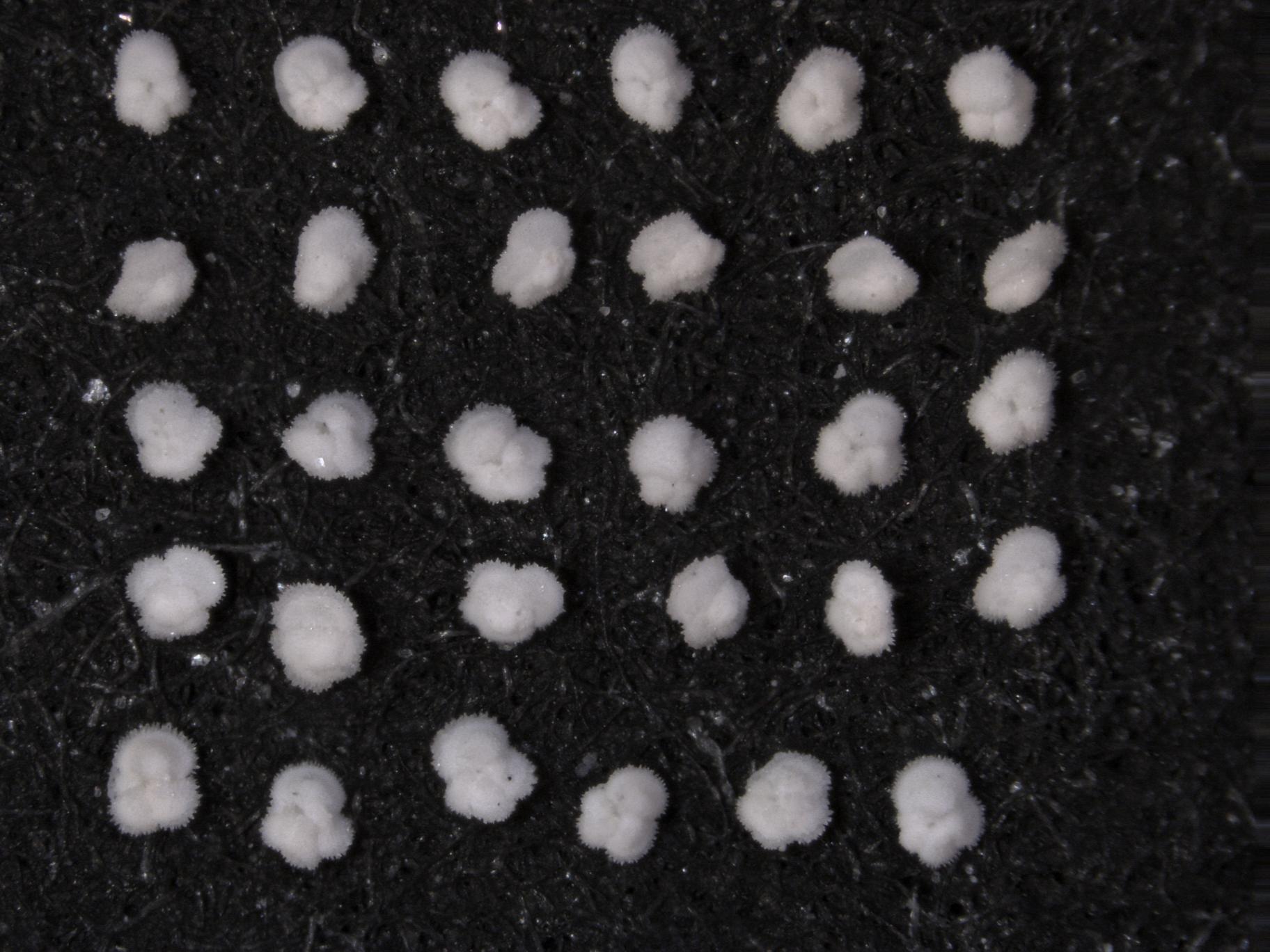

Supplement: S1 Data — (ZIP) [file pone.0267636.s001.zip › SDataImages/1209A-21H-2W_146-148_180_Mor1_3.2x_STACKED.jpg]

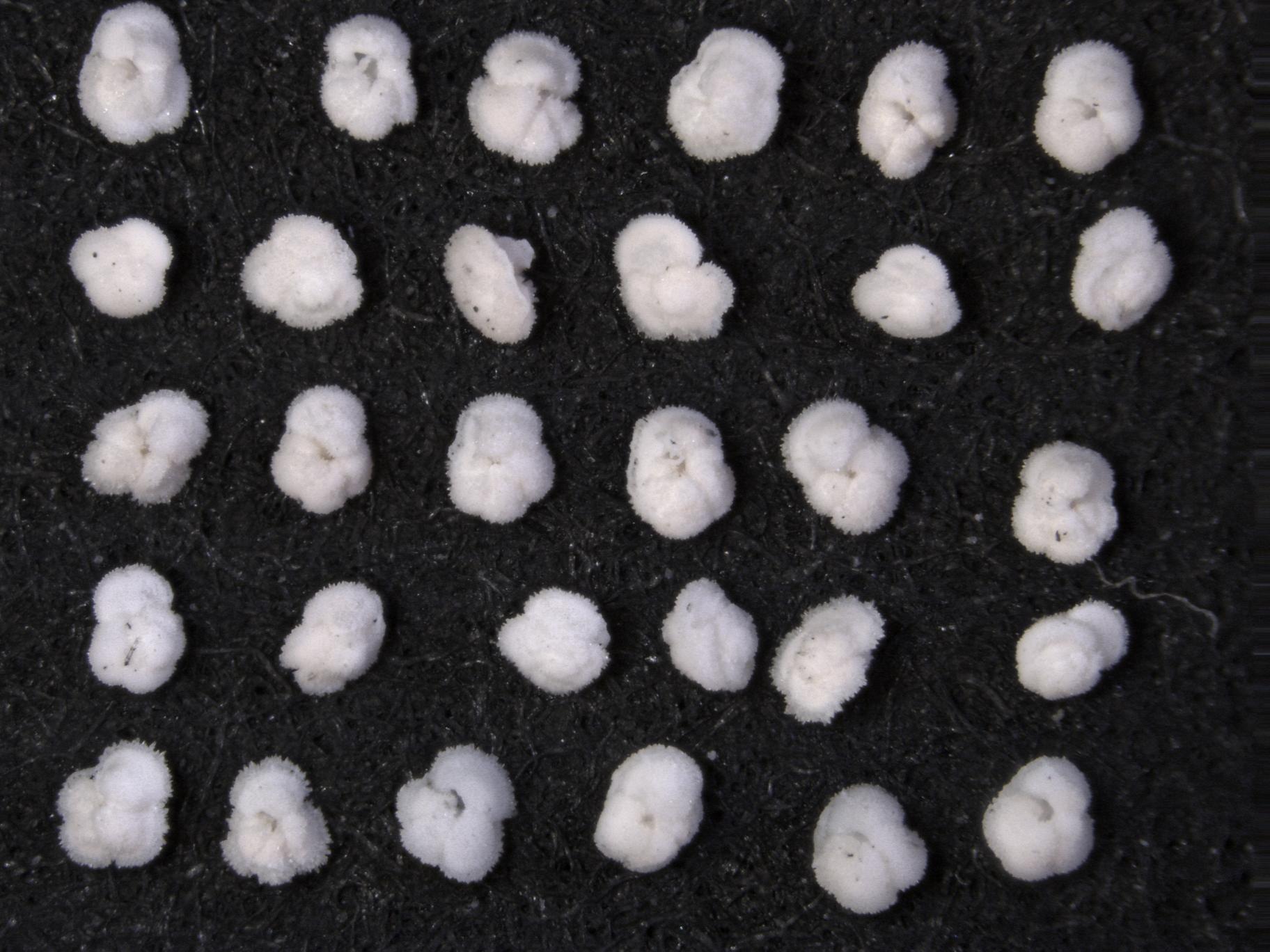

Supplement: S1 Data — (ZIP) [file pone.0267636.s001.zip › SDataImages/1209A-21H-3W_18-20_300_Mor1_2.5x_STACKED.jpg]

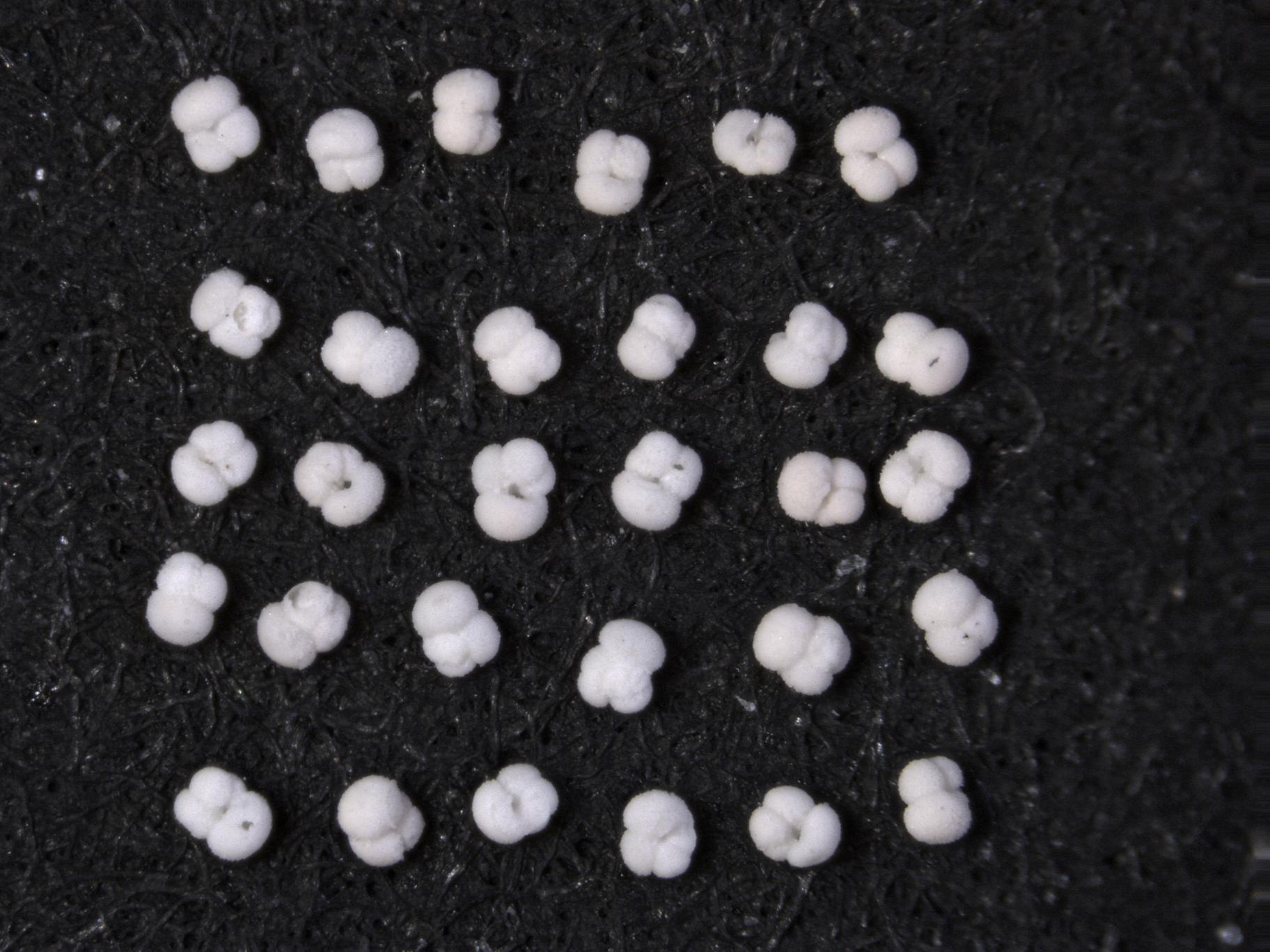

Supplement: S1 Data — (ZIP) [file pone.0267636.s001.zip › SDataImages/1209A-21H-3W_117-119_180_Sub1_3.2x_STACKED.jpg]

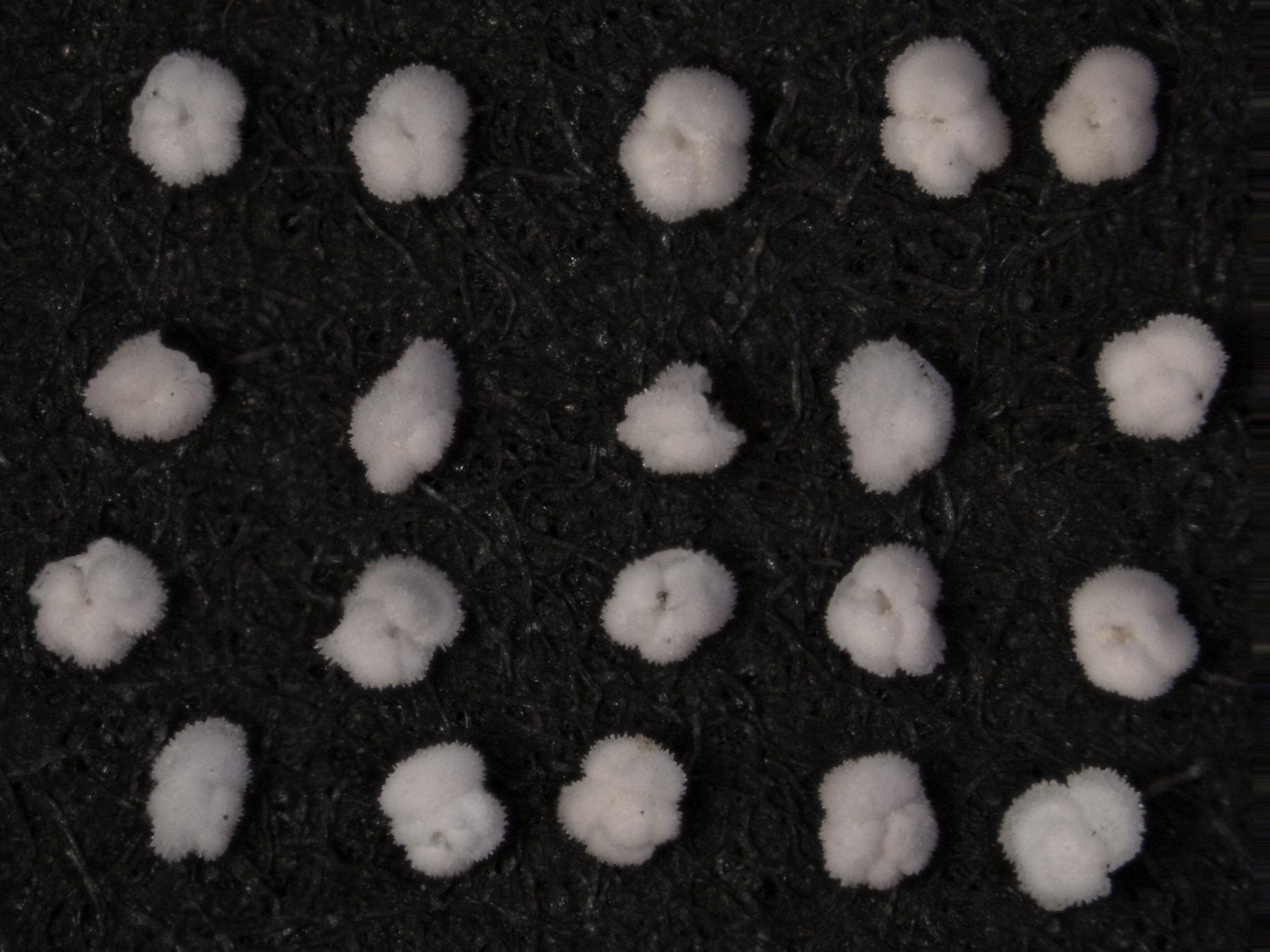

Supplement: S1 Data — (ZIP) [file pone.0267636.s001.zip › SDataImages/1209A-21H-3W_87-89_212_Mor1_4.0x_STACKED.jpg]

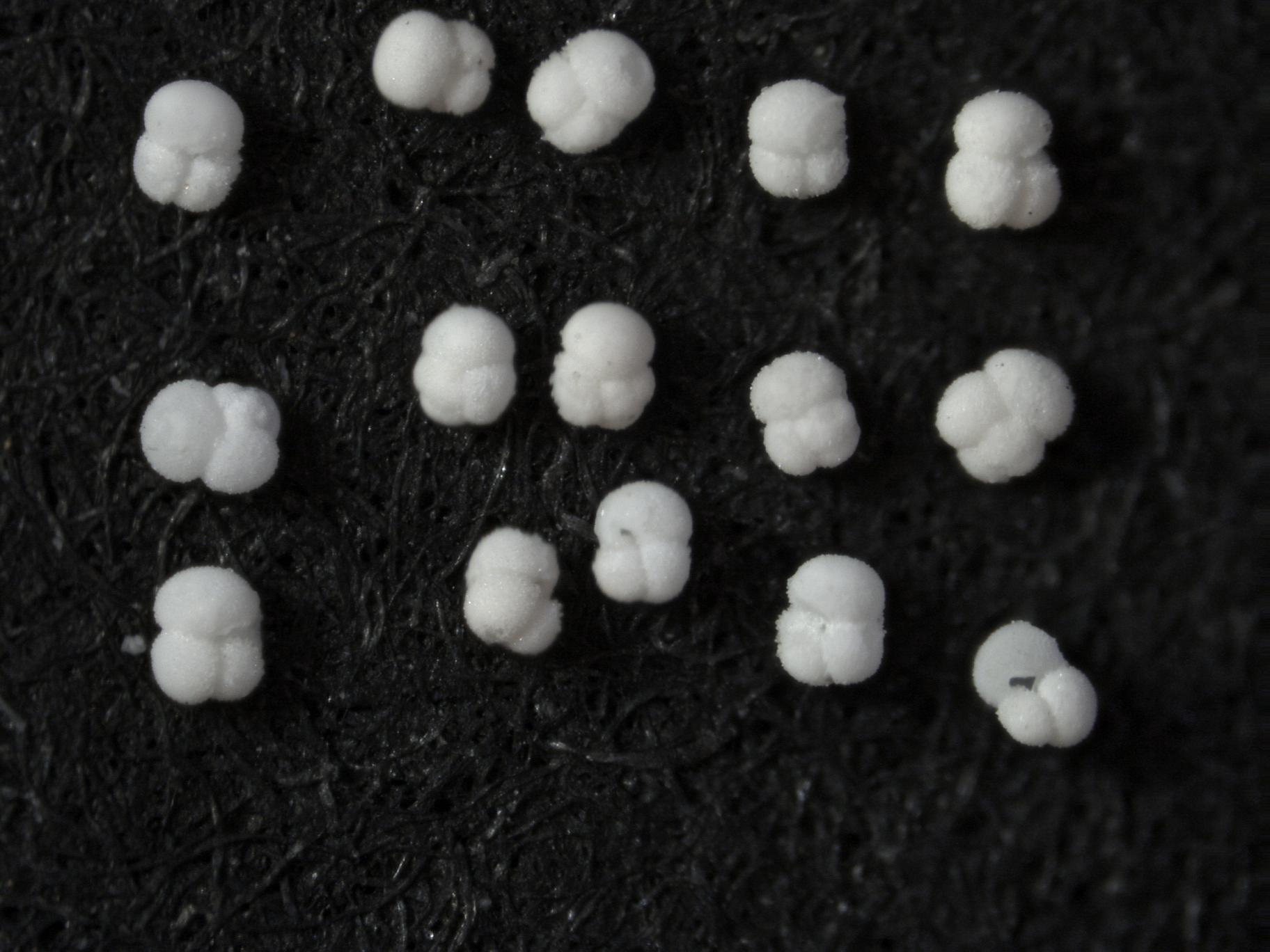

Supplement: S1 Data — (ZIP) [file pone.0267636.s001.zip › SDataImages/1209A-21H-3W_38-40_212_Sub1_4.0x_STACKED.jpg]

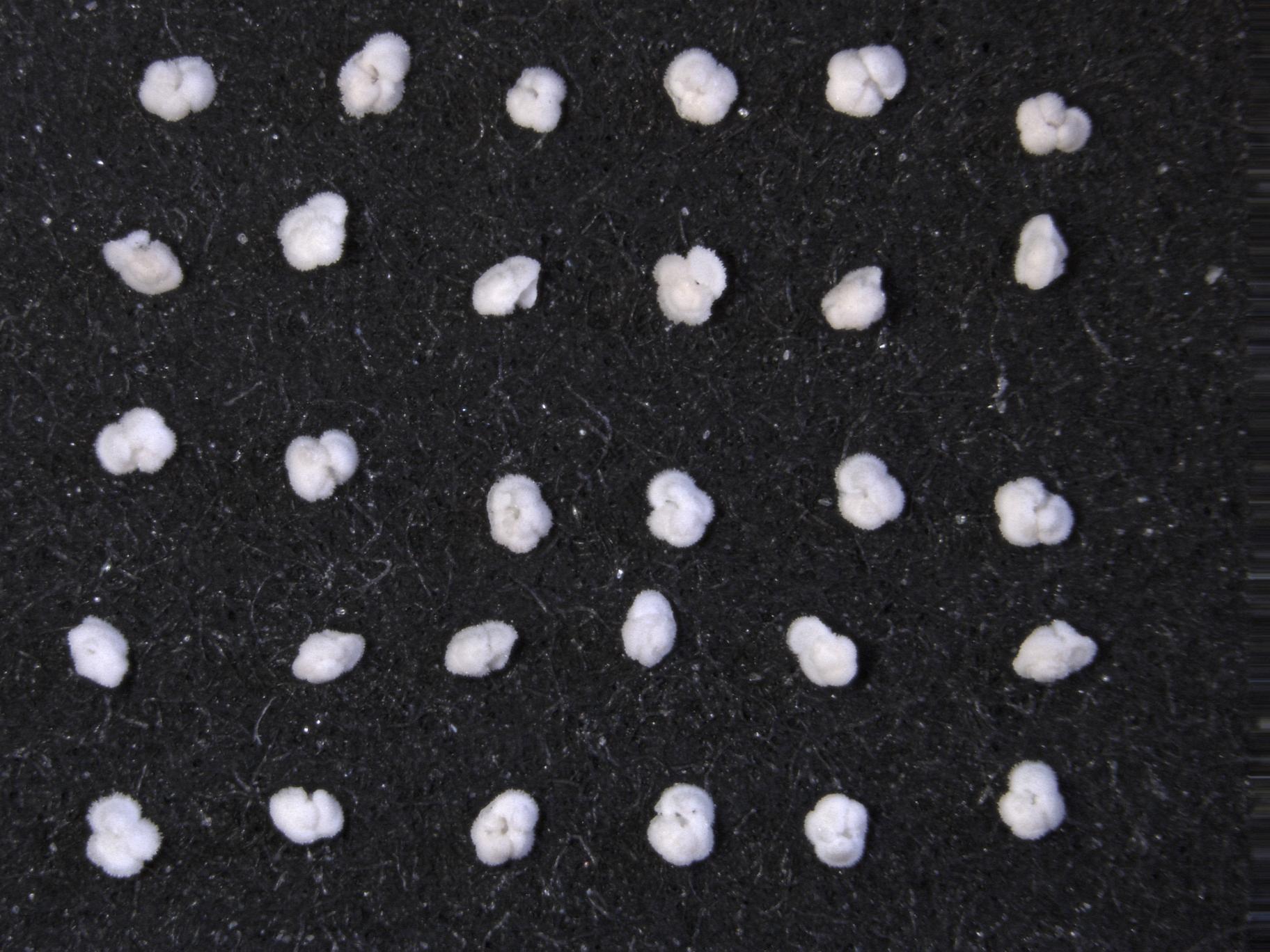

Supplement: S1 Data — (ZIP) [file pone.0267636.s001.zip › SDataImages/1209A-21H-2W_86-88_300_Mor1_1.6x_STACKED.jpg]

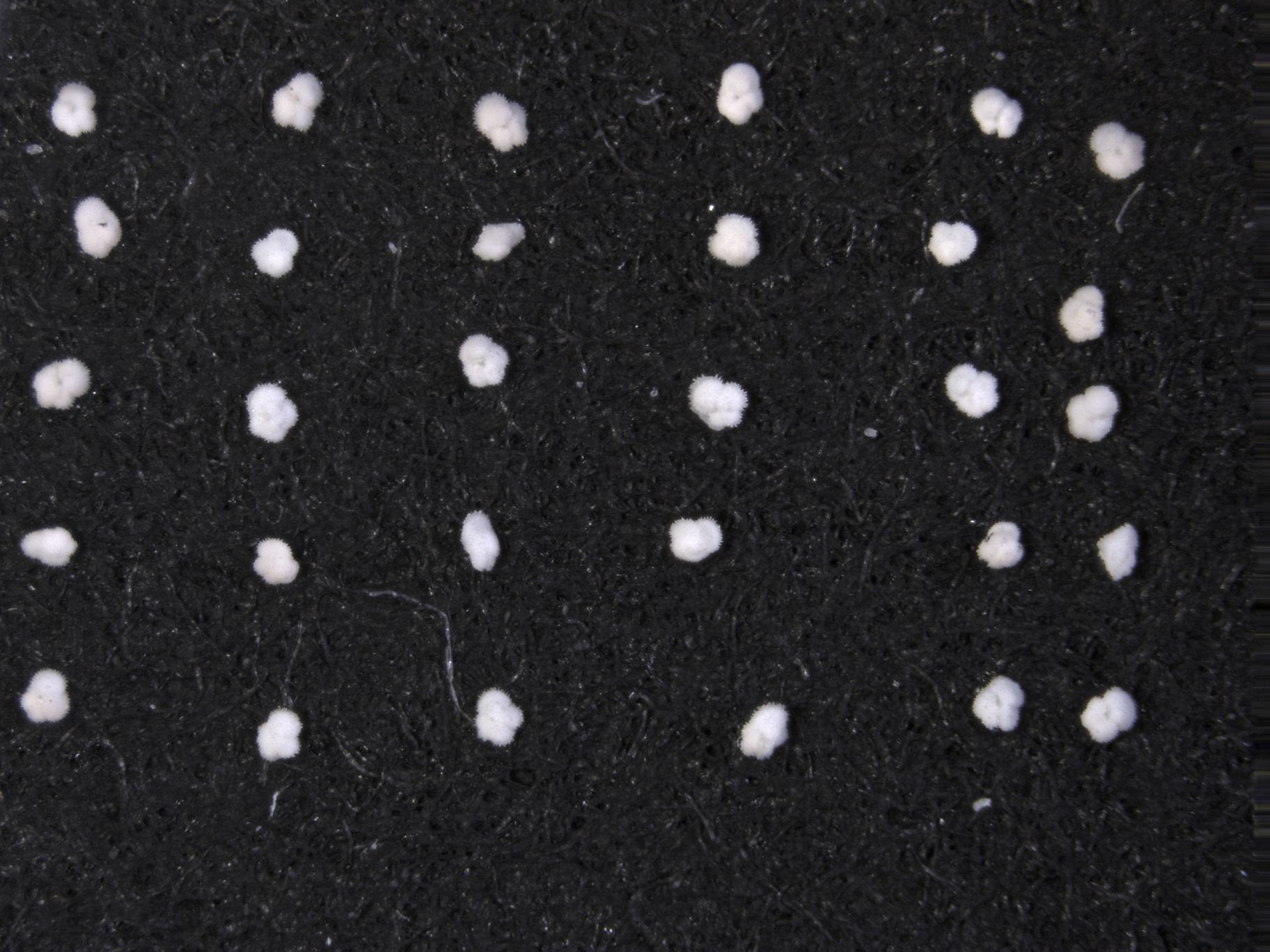

Supplement: S1 Data — (ZIP) [file pone.0267636.s001.zip › SDataImages/1209A-21H-3W_117-119_150_Mor1_2.0x_STACKED.jpg]

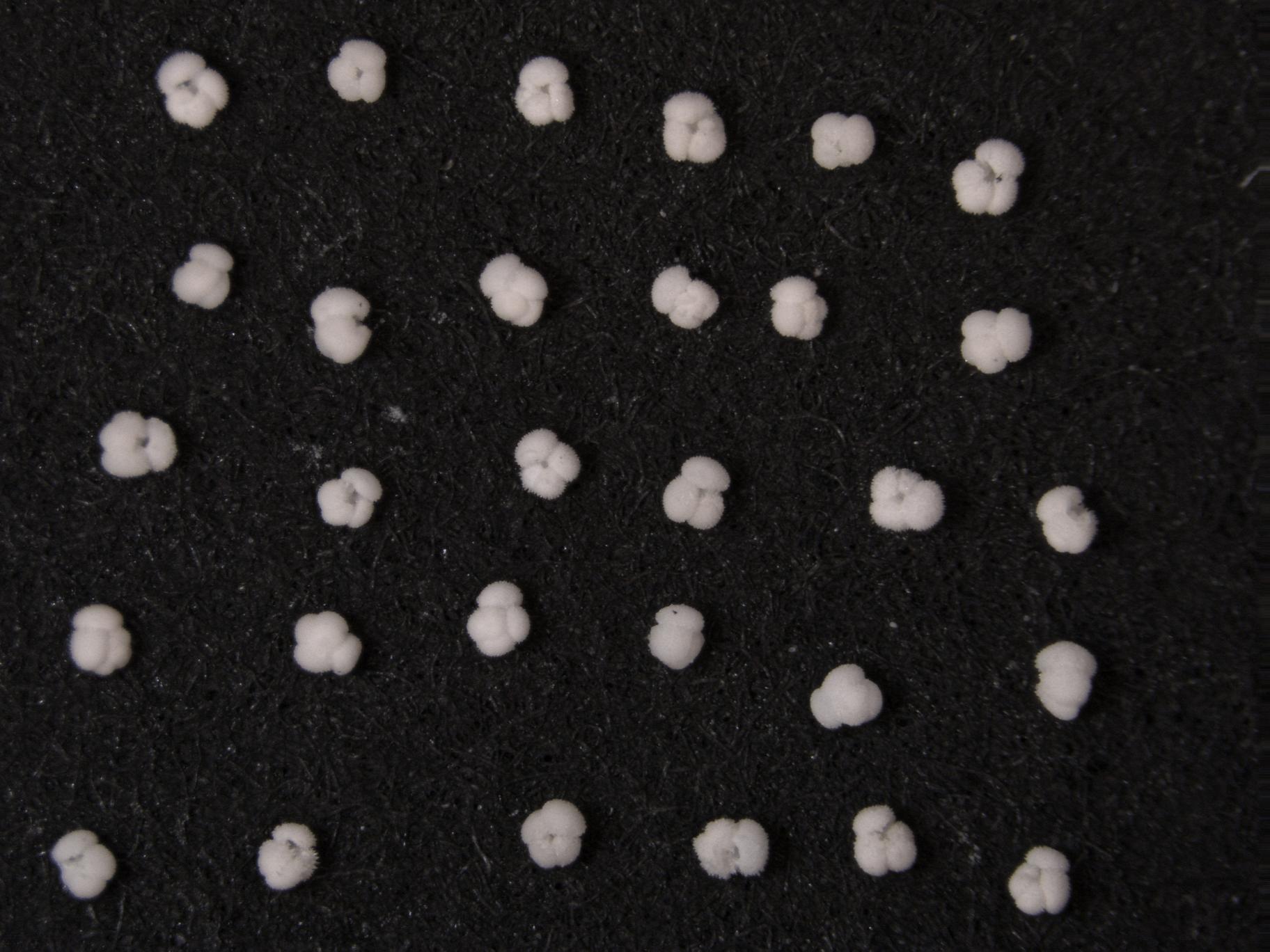

Supplement: S1 Data — (ZIP) [file pone.0267636.s001.zip › SDataImages/1209A-21H-3W_47-49_300_Aca1_1.6x_STACKED.jpg]

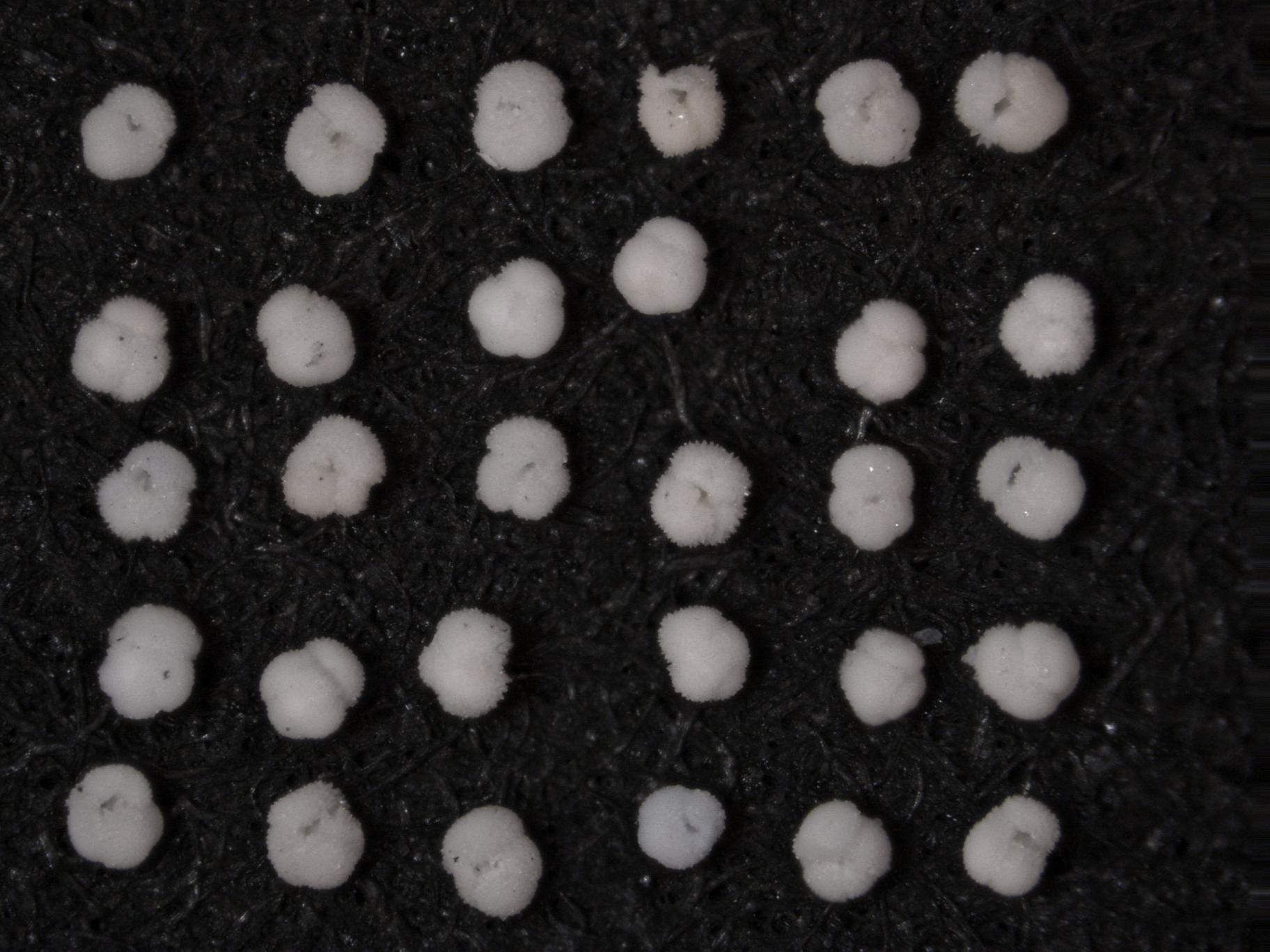

Supplement: S1 Data — (ZIP) [file pone.0267636.s001.zip › SDataImages/1209A-21H-3W_18-20_150_Aca1_4.0x_STACKED.jpg]

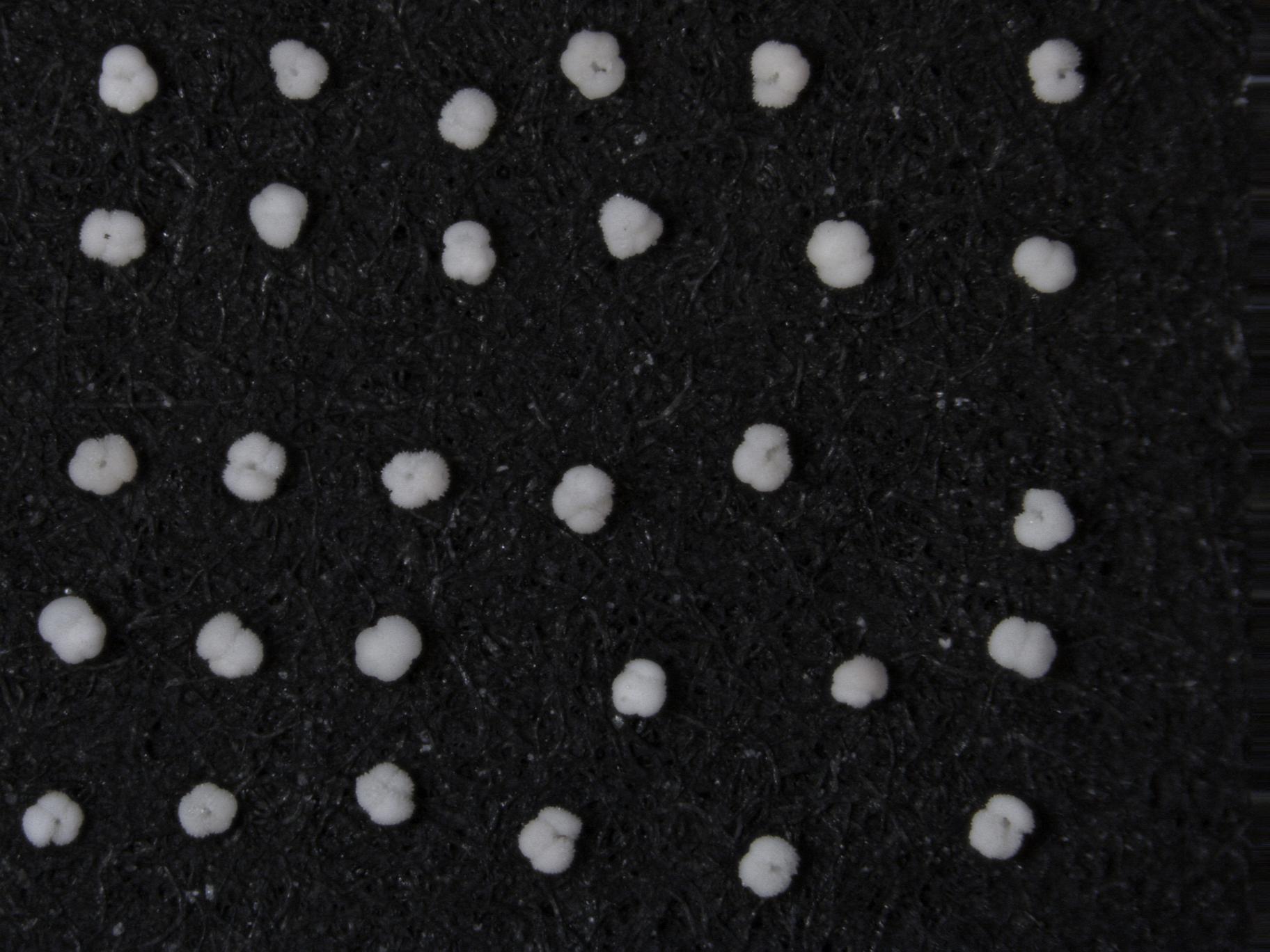

Supplement: S1 Data — (ZIP) [file pone.0267636.s001.zip › SDataImages/1209A-21H-3W_47-49_180_Aca1_2.5x_STACKED.jpg]

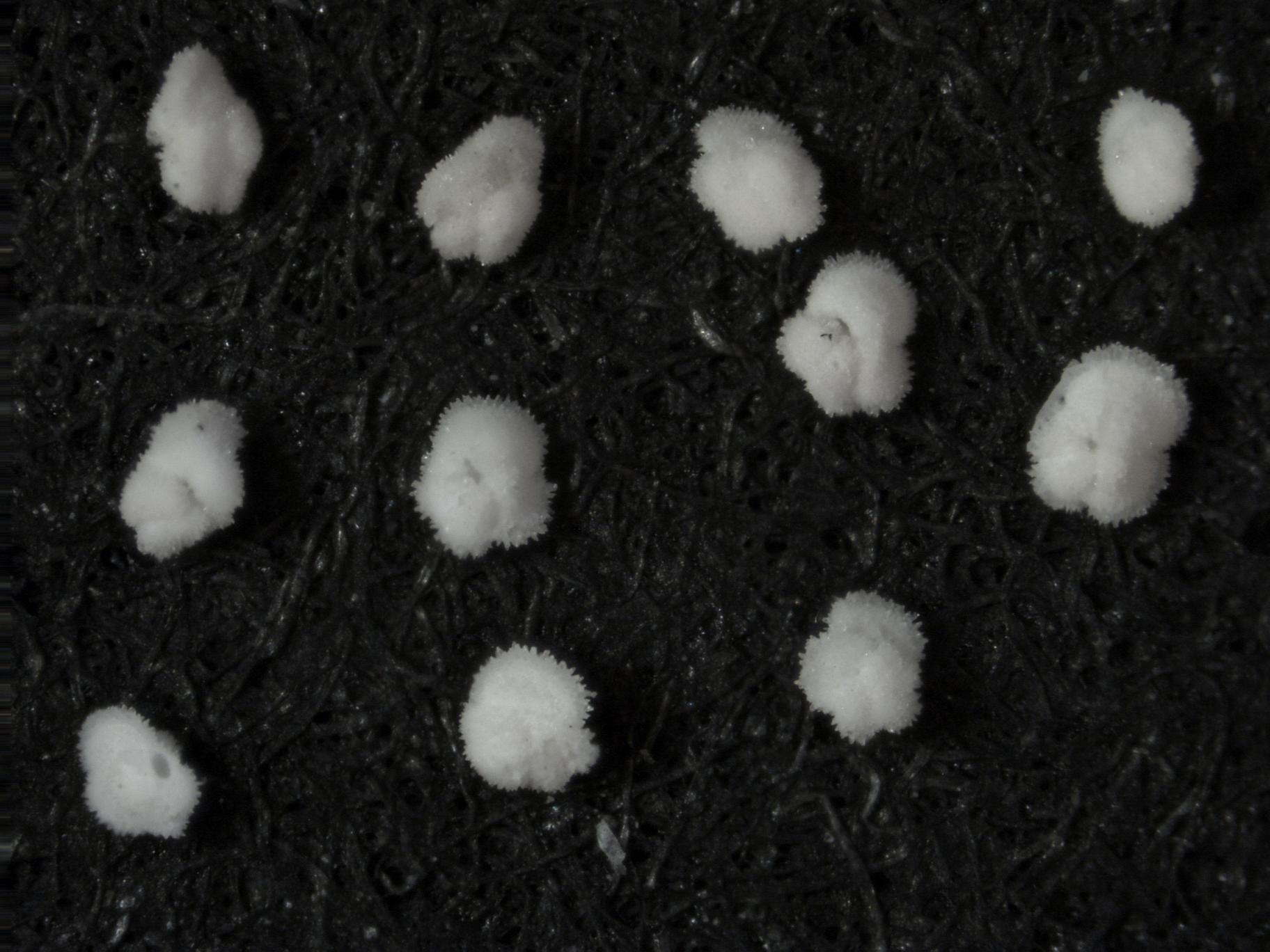

Supplement: S1 Data — (ZIP) [file pone.0267636.s001.zip › SDataImages/1209A-21H-3W_38-40_180_Mor3_5.0x_STACKED.jpg]

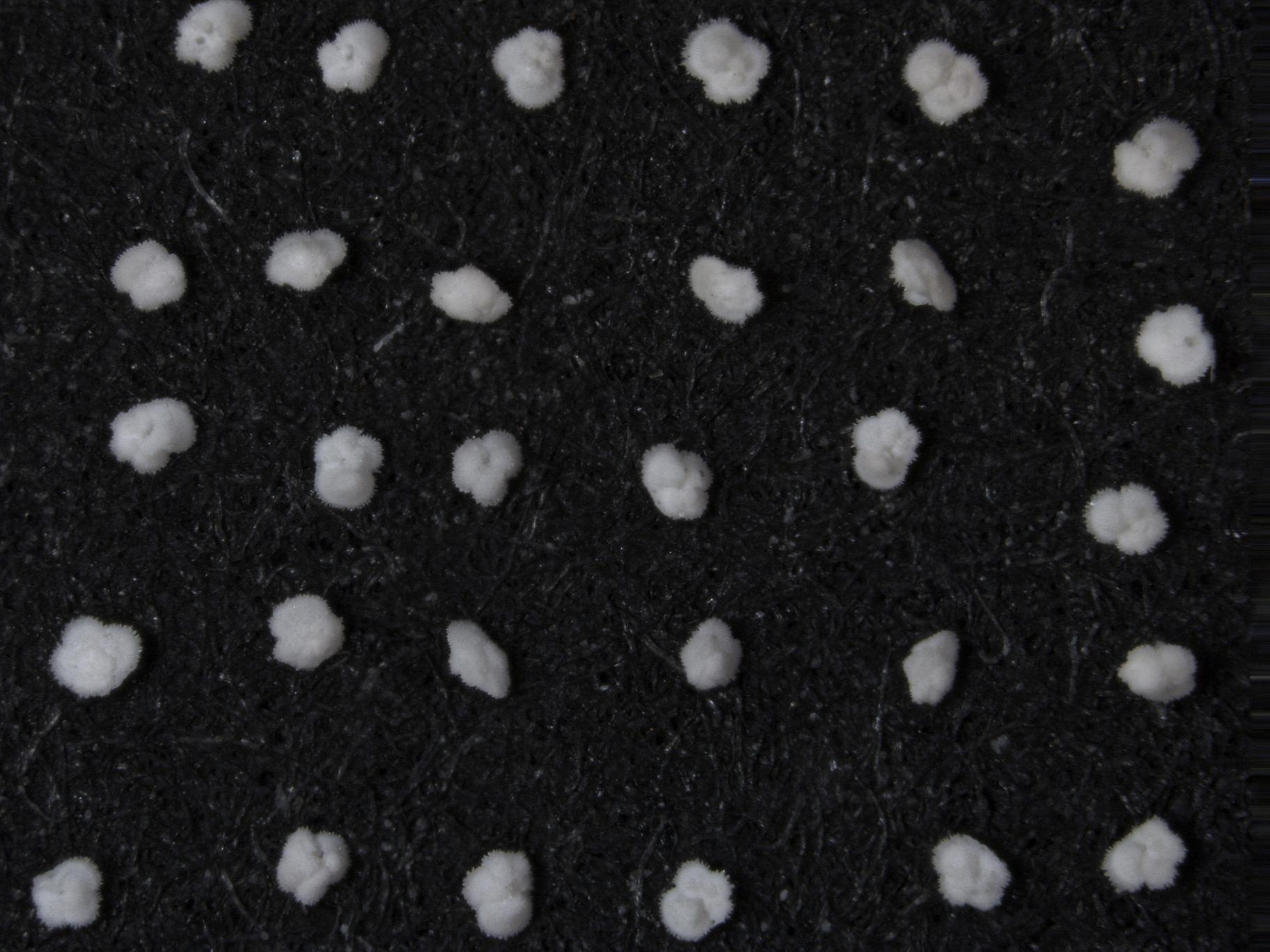

Supplement: S1 Data — (ZIP) [file pone.0267636.s001.zip › SDataImages/1209A-21H-3W_7-9_180_Mor1_2.5x_STACKED.jpg]

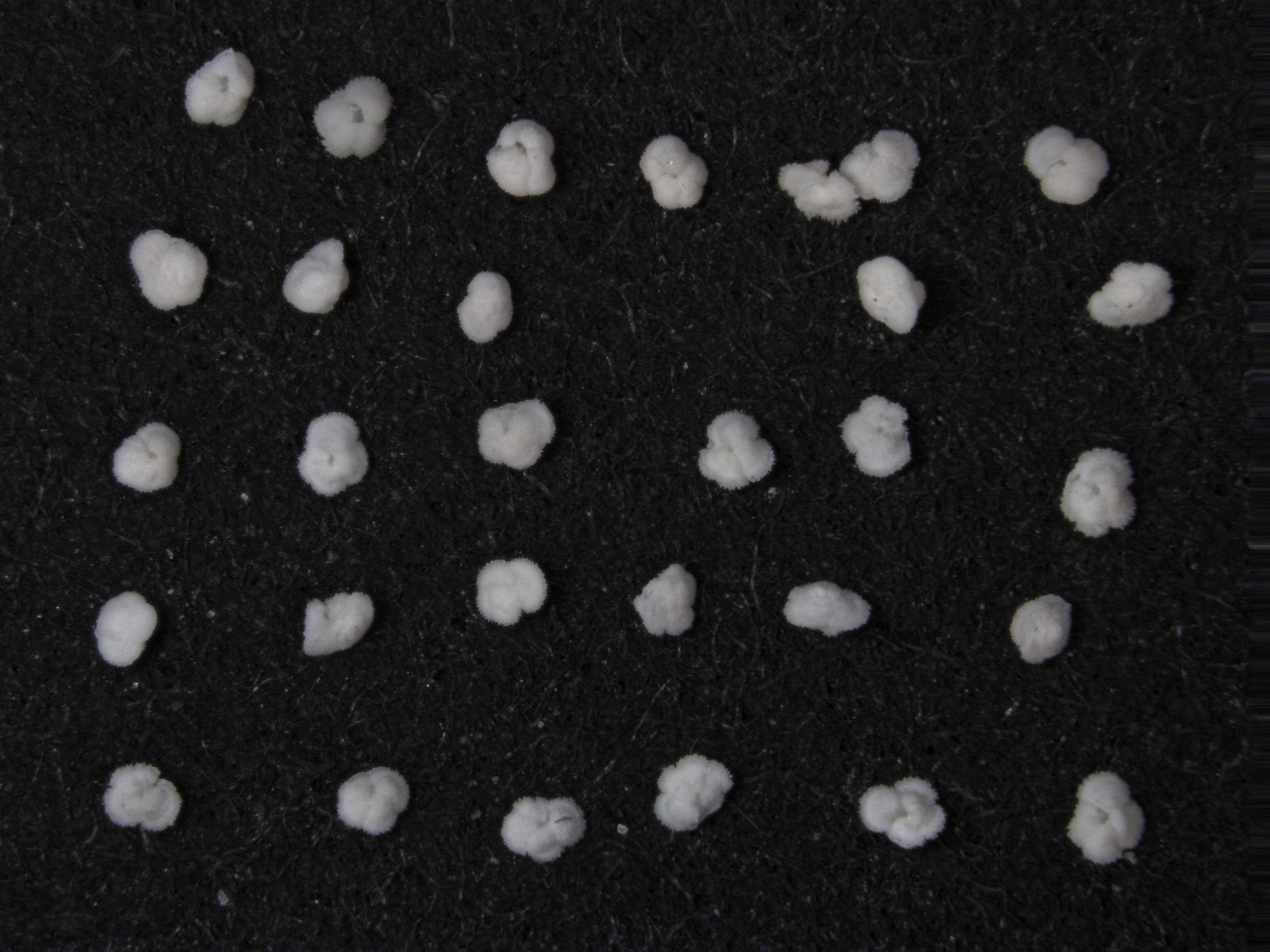

Supplement: S1 Data — (ZIP) [file pone.0267636.s001.zip › SDataImages/1209A-21H-3W_7-9_300_Mor1_1.6x_STACKED.jpg]

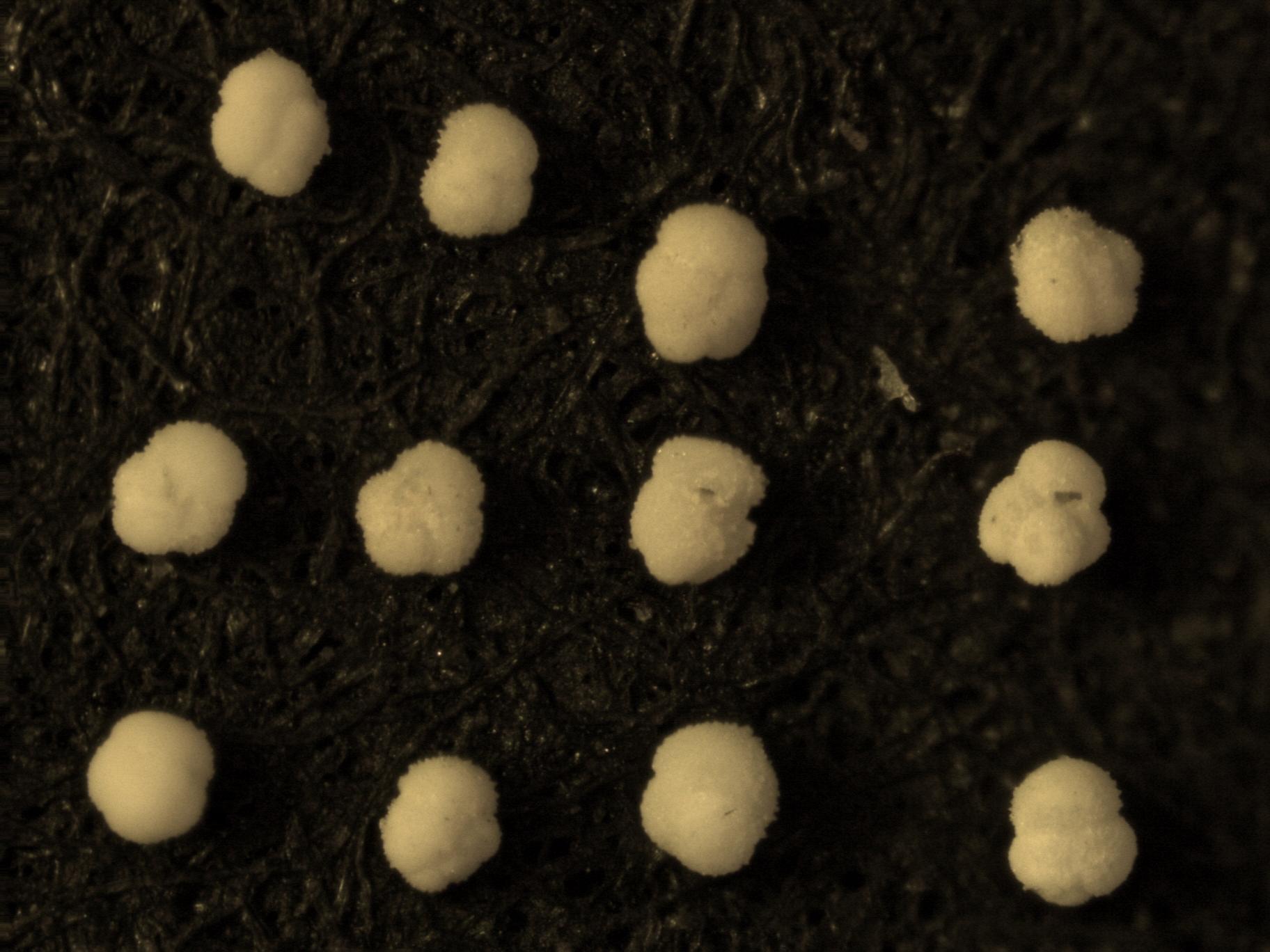

Supplement: S1 Data — (ZIP) [file pone.0267636.s001.zip › SDataImages/1209A-21H-3W_58-60_150_Aca2_6.3x_STACKED.jpg]

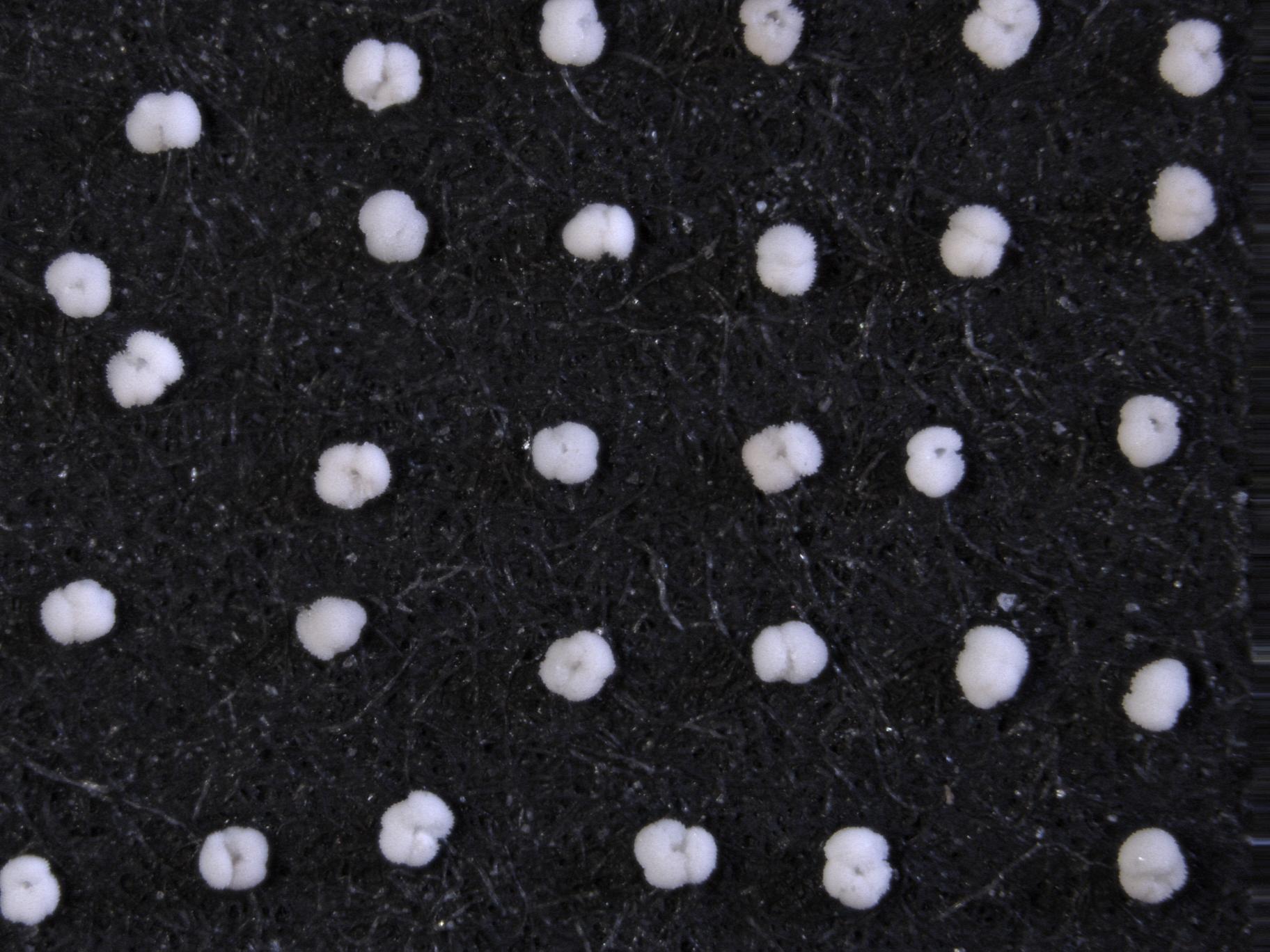

Supplement: S1 Data — (ZIP) [file pone.0267636.s001.zip › SDataImages/1209A-21H-2W_86-88_212_Aca1_2.5x_STACKED.jpg]

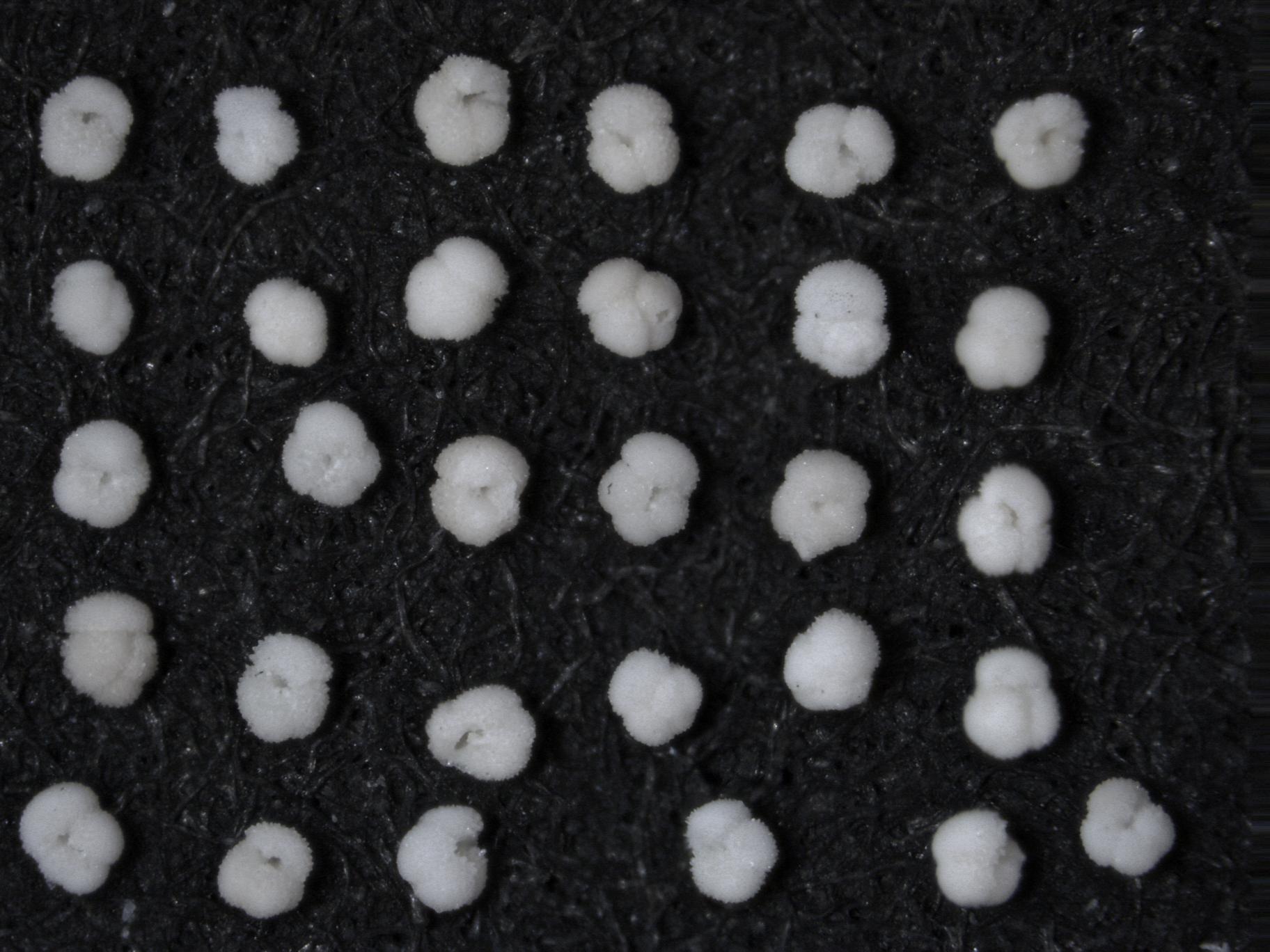

Supplement: S1 Data — (ZIP) [file pone.0267636.s001.zip › SDataImages/1209A-21H-3W_87-89_180_Aca1_4.0x_STACKED.jpg]

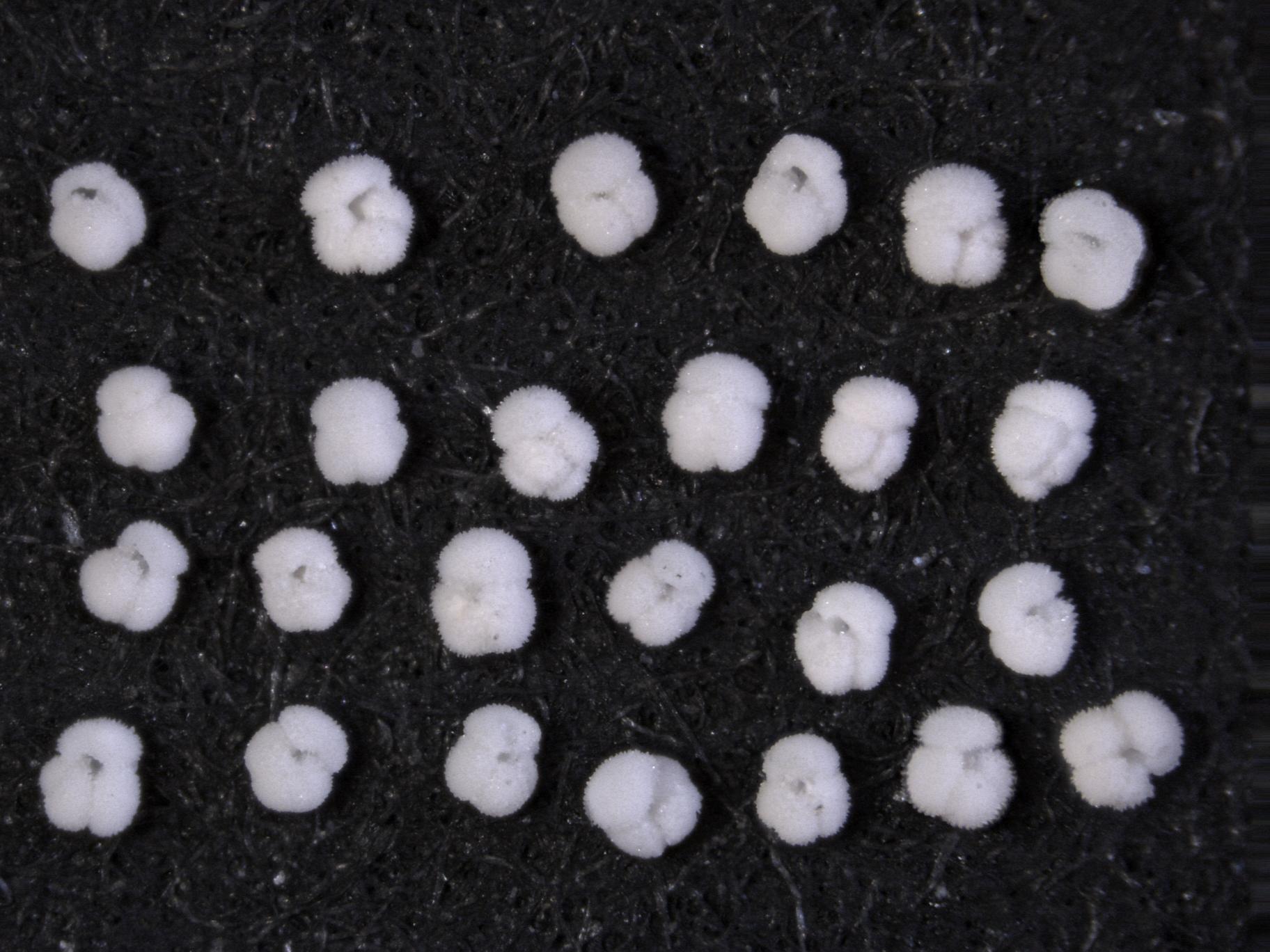

Supplement: S1 Data — (ZIP) [file pone.0267636.s001.zip › SDataImages/1209A-21H-2W_146-148_250_Aca1_3.2x_STACKED.jpg]

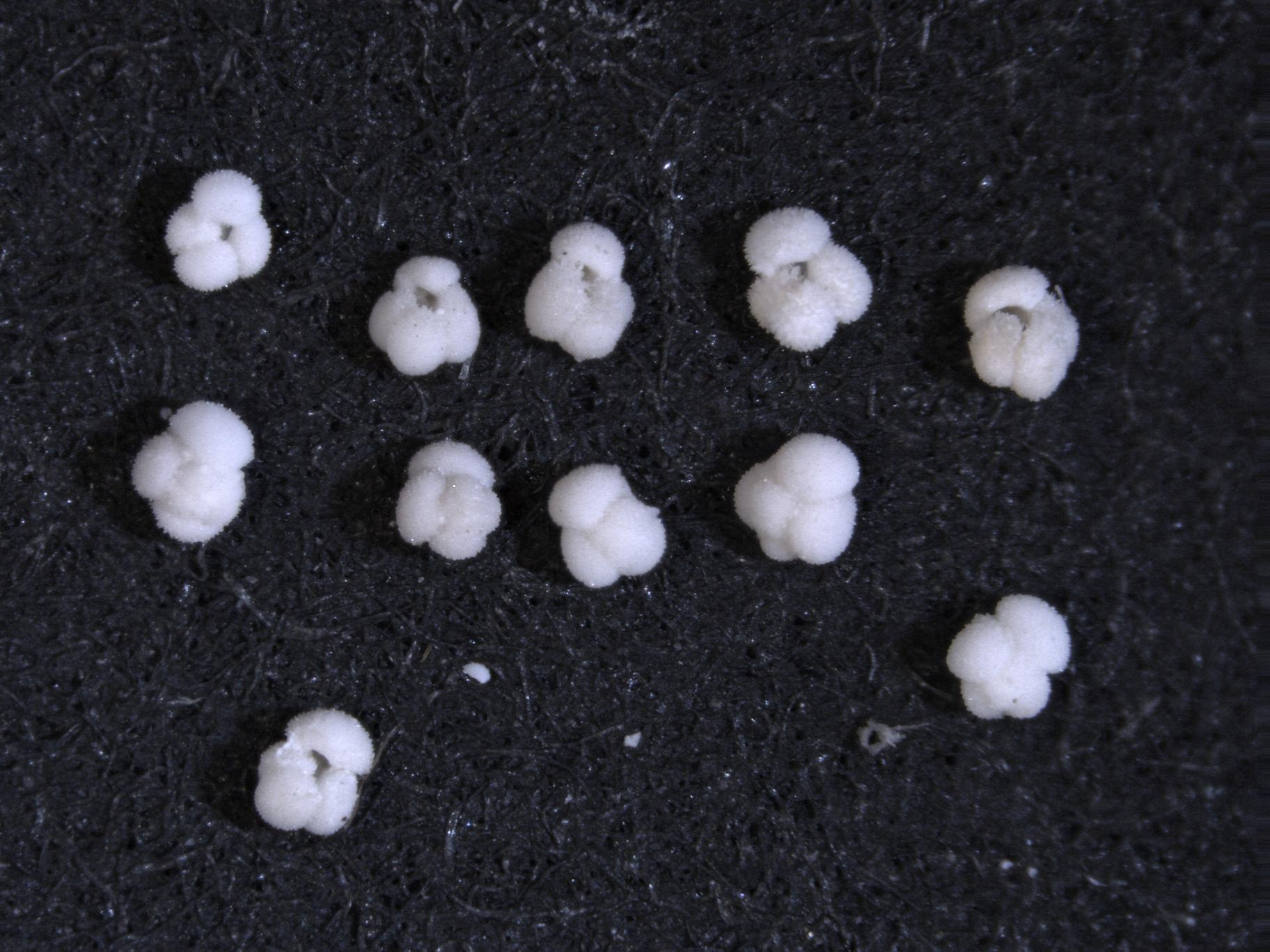

Supplement: S1 Data — (ZIP) [file pone.0267636.s001.zip › SDataImages/1209A-21H-3W_87-89_355_Aca2_2.5x_STACKED.jpg]

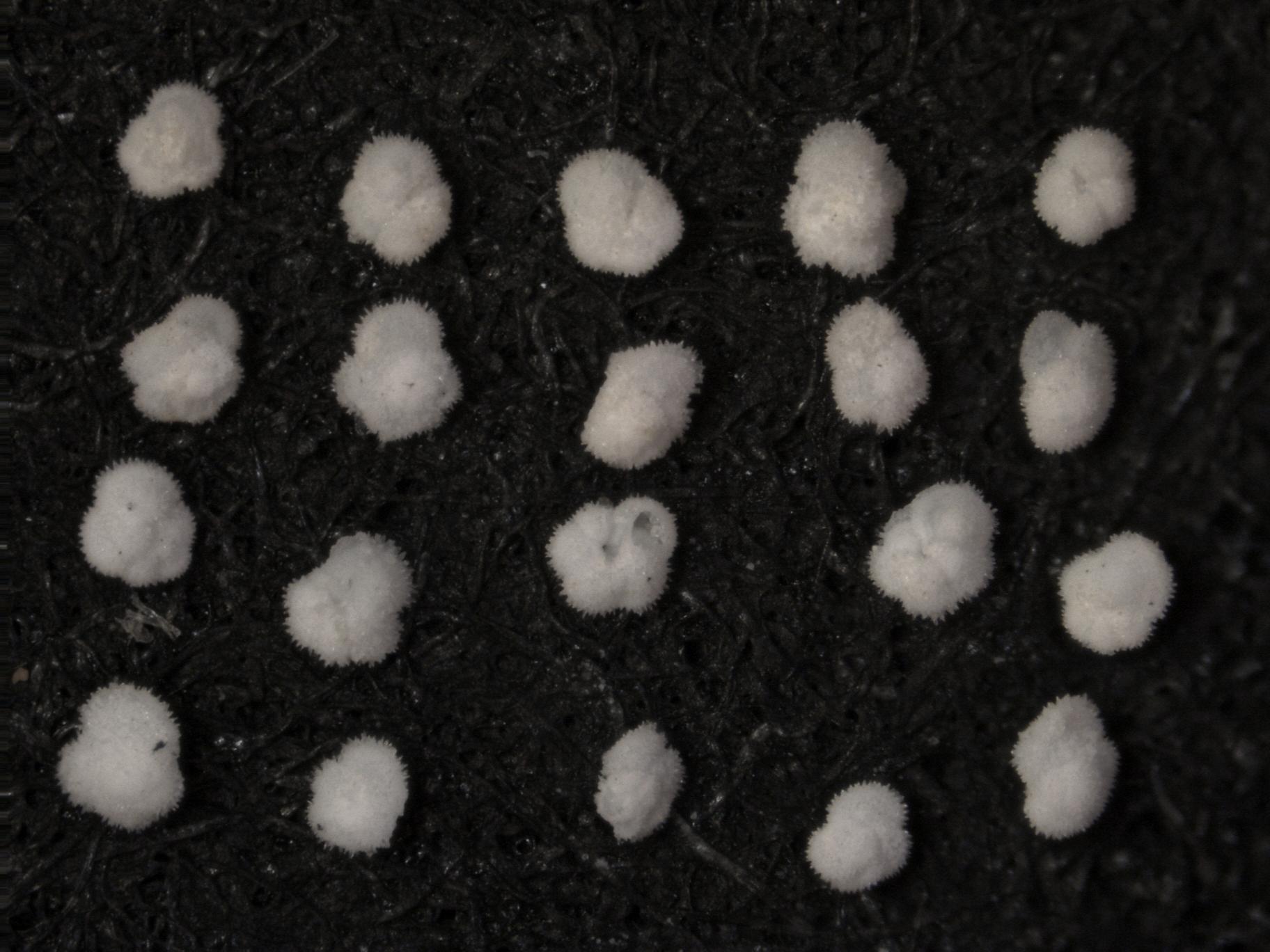

Supplement: S1 Data — (ZIP) [file pone.0267636.s001.zip › SDataImages/1209A-21H-3W_58-60_150_Mor1_5.0x_STACKED.jpg]

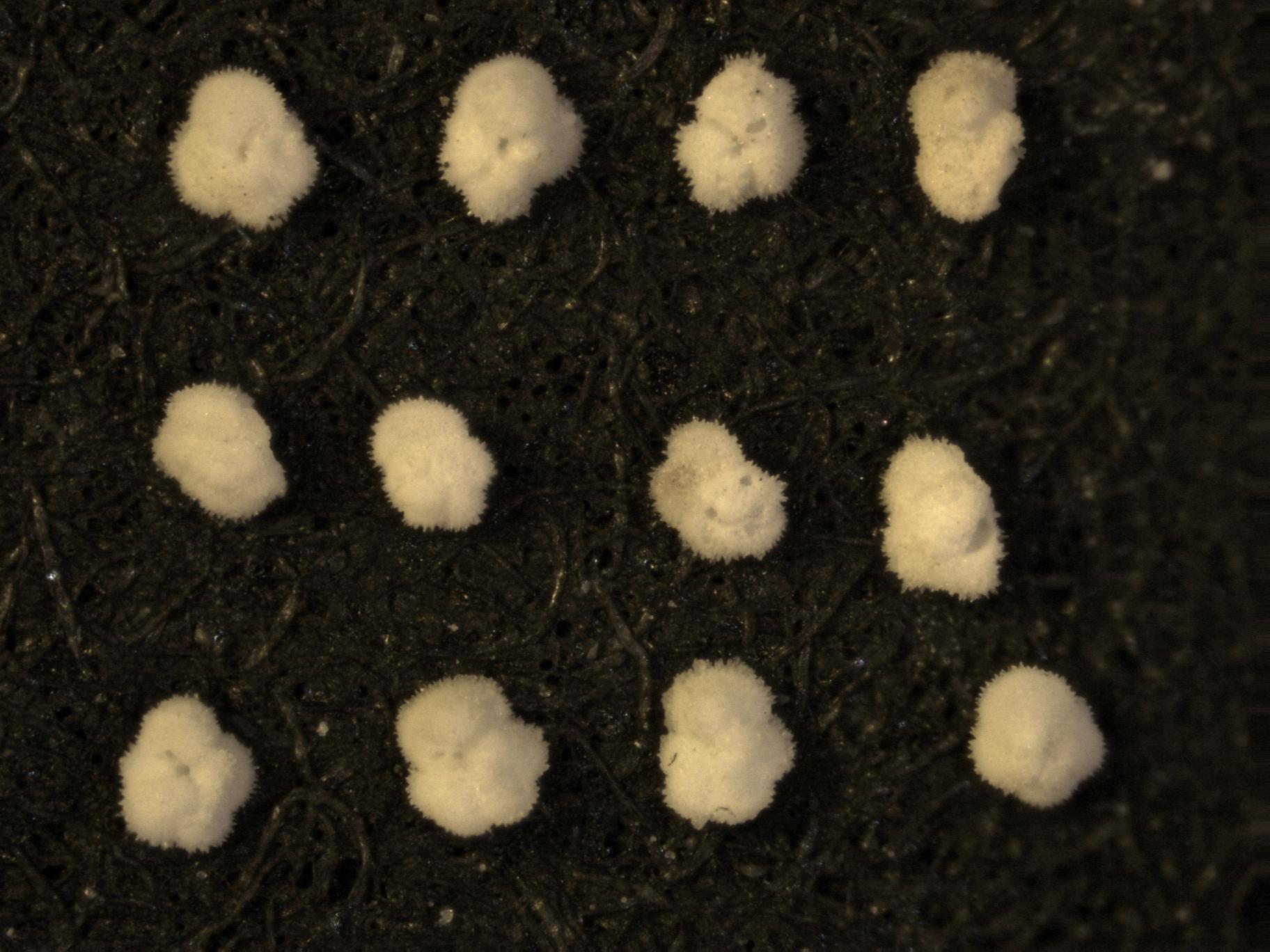

Supplement: S1 Data — (ZIP) [file pone.0267636.s001.zip › SDataImages/1209A-21H-3W_58-60_180_Mor1_5.0x_STACKED.jpg]

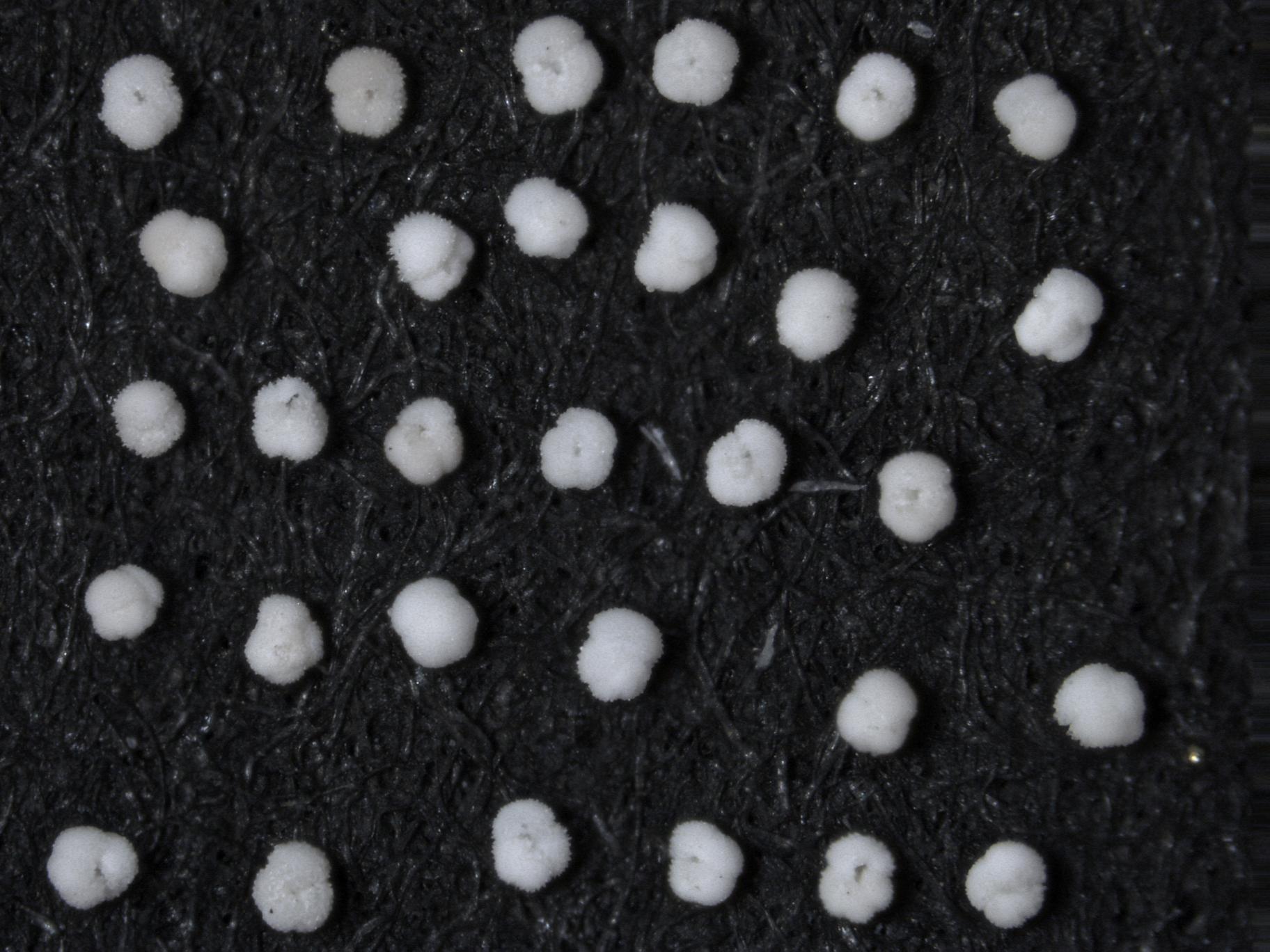

Supplement: S1 Data — (ZIP) [file pone.0267636.s001.zip › SDataImages/1209A-21H-3W_87-89_150_Aca1_4.0x_STACKED.jpg]

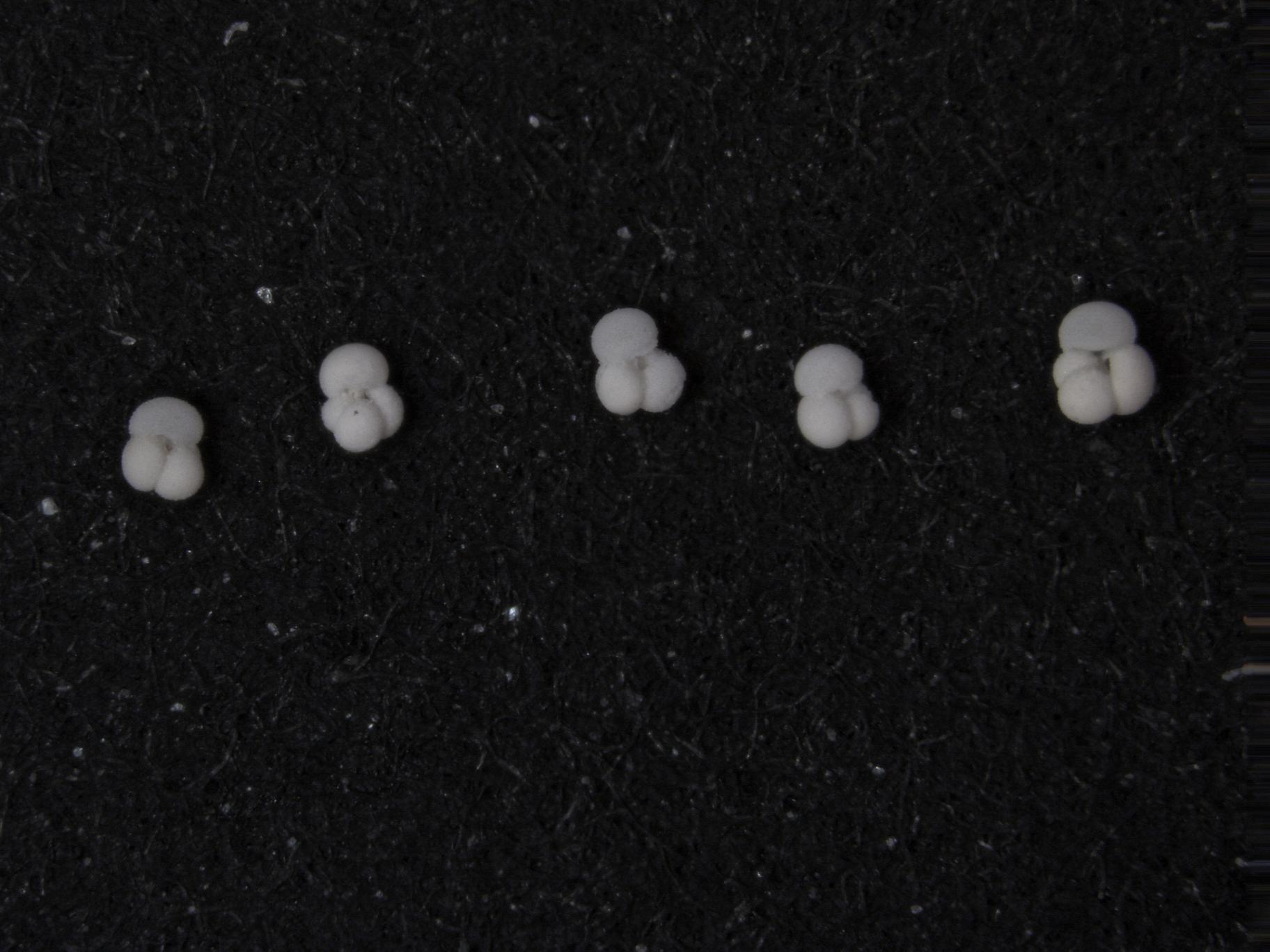

Supplement: S1 Data — (ZIP) [file pone.0267636.s001.zip › SDataImages/1209A-21H-3W_77-79_355_Sub1_2.0x_STACKED.jpg]

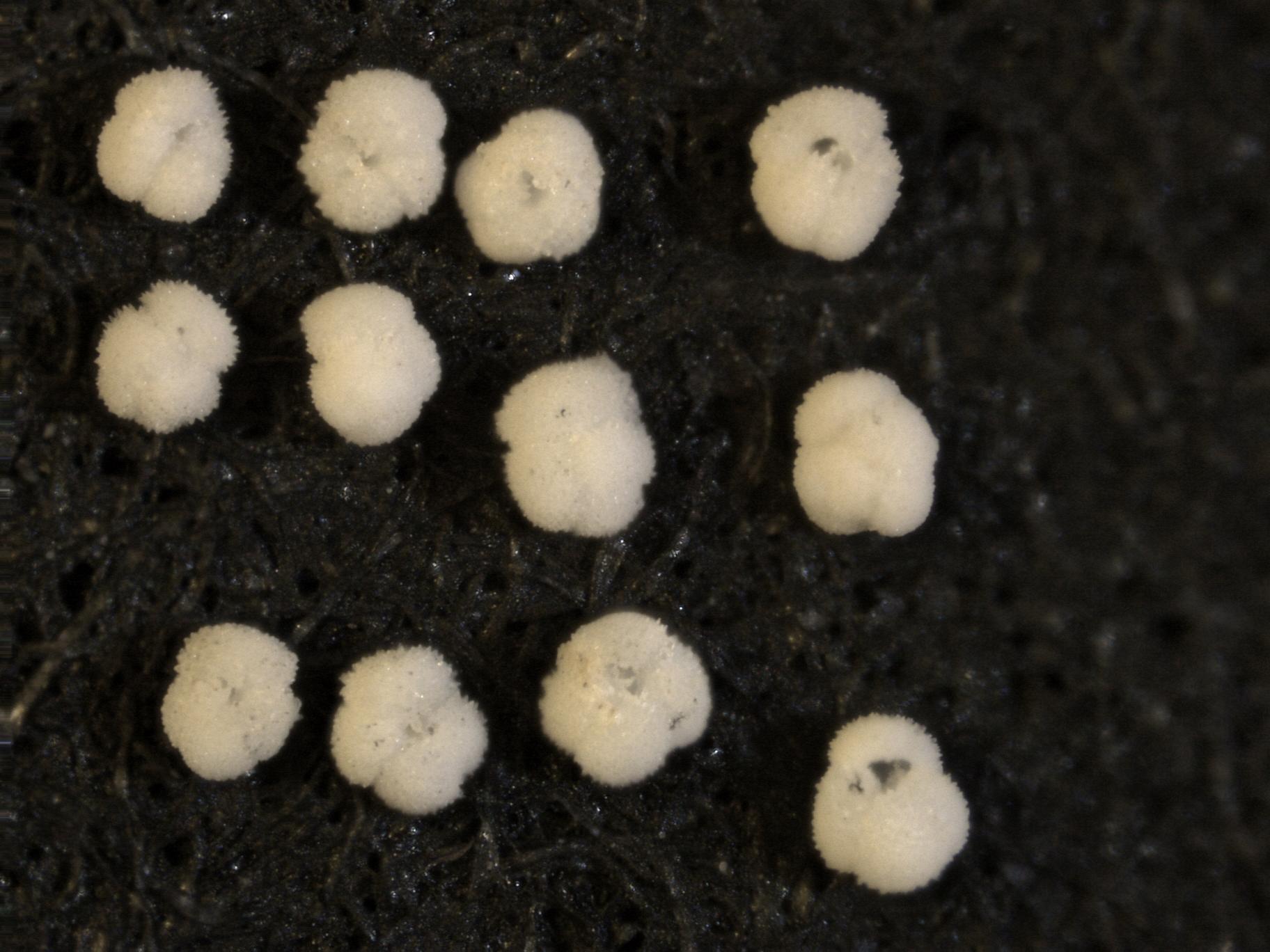

Supplement: S1 Data — (ZIP) [file pone.0267636.s001.zip › SDataImages/1209A-21H-3W_58-60_180_Aca2_6.3x_STACKED.jpg]

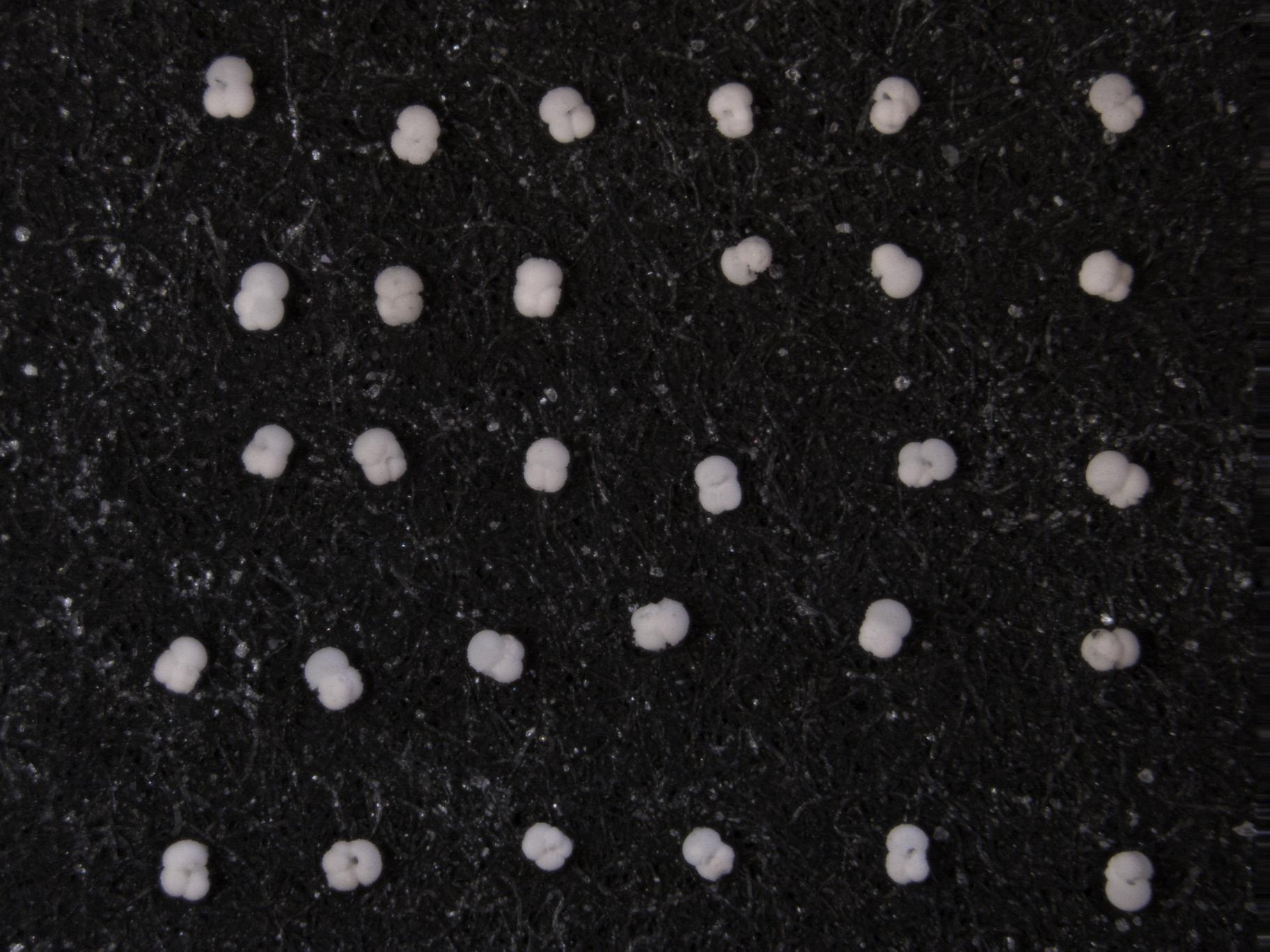

Supplement: S1 Data — (ZIP) [file pone.0267636.s001.zip › SDataImages/1209A-21H-2W_86-88_180_Sub1_2.0x_STACKED.jpg]

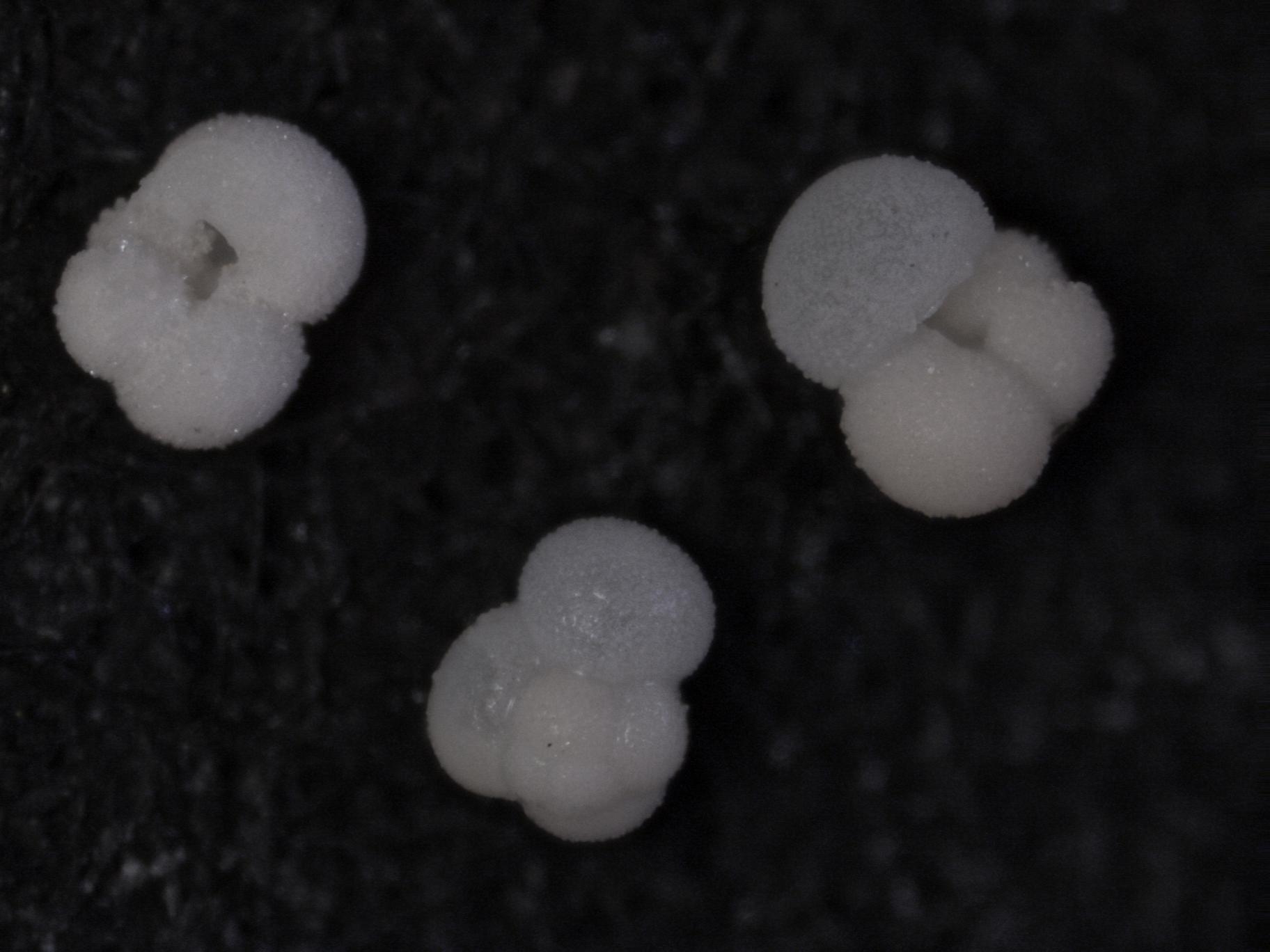

Supplement: S1 Data — (ZIP) [file pone.0267636.s001.zip › SDataImages/1209A-21H-3W_18-20_355_Sub1_6.3x_STACKED.jpg]

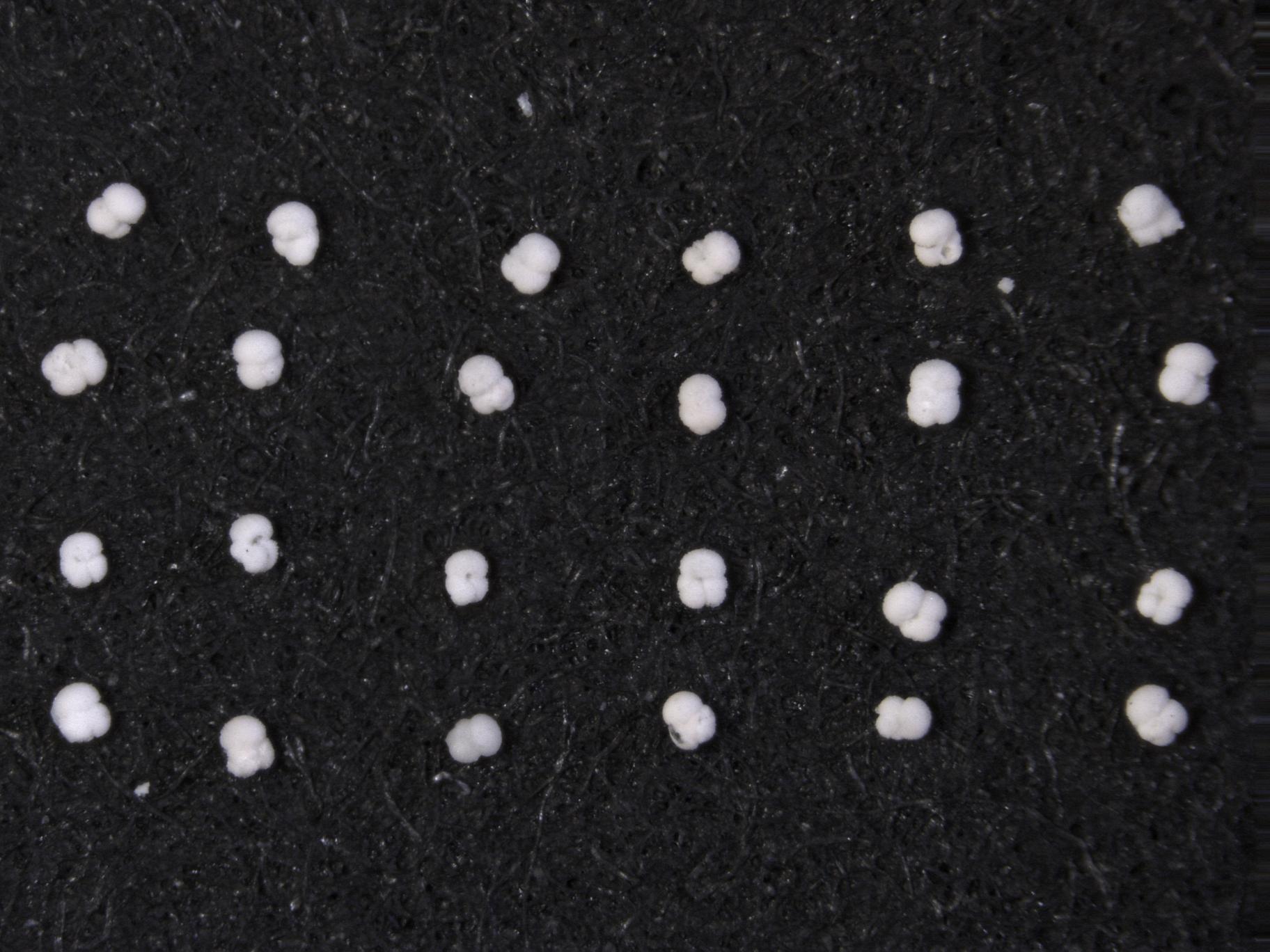

Supplement: S1 Data — (ZIP) [file pone.0267636.s001.zip › SDataImages/1209A-21H-3W_117-119_150_Sub1_2.5x_STACKED.jpg]

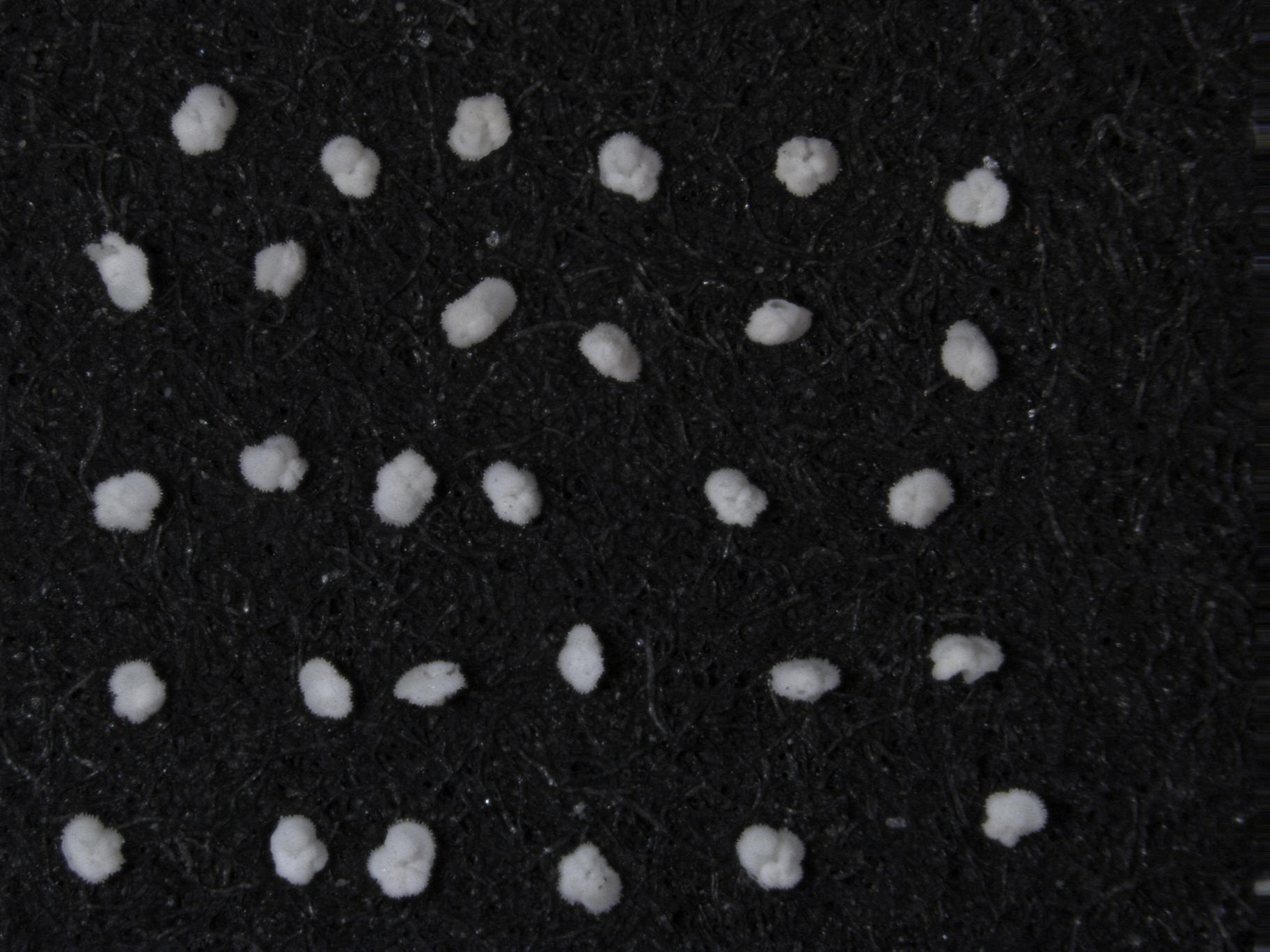

Supplement: S1 Data — (ZIP) [file pone.0267636.s001.zip › SDataImages/1209A-21H-3W_7-9_150_Mor1_2.5x_STACKED.jpg]

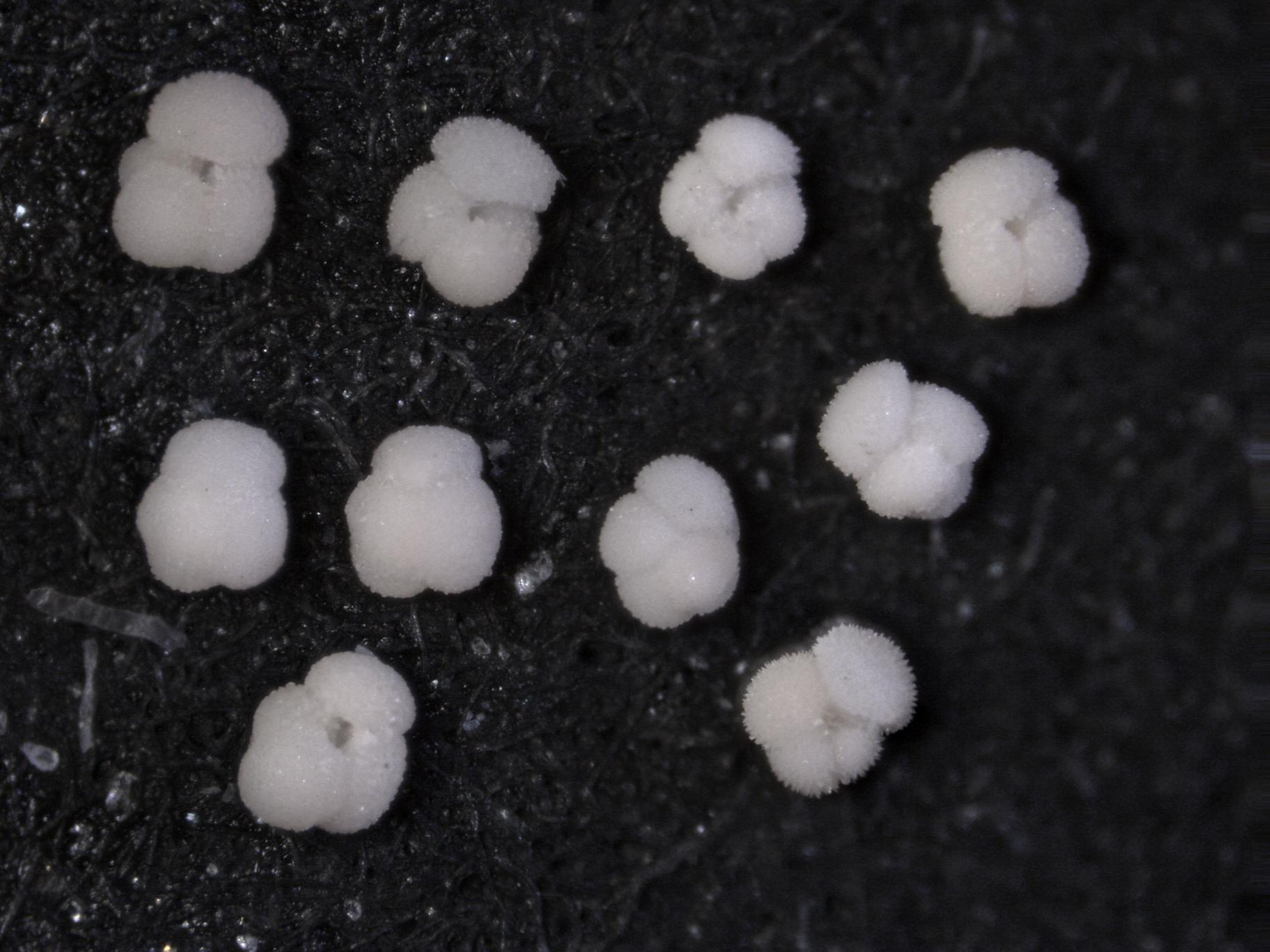

Supplement: S1 Data — (ZIP) [file pone.0267636.s001.zip › SDataImages/1209A-21H-2W_146-148_300_Aca1_4.0x_STACKED.jpg]

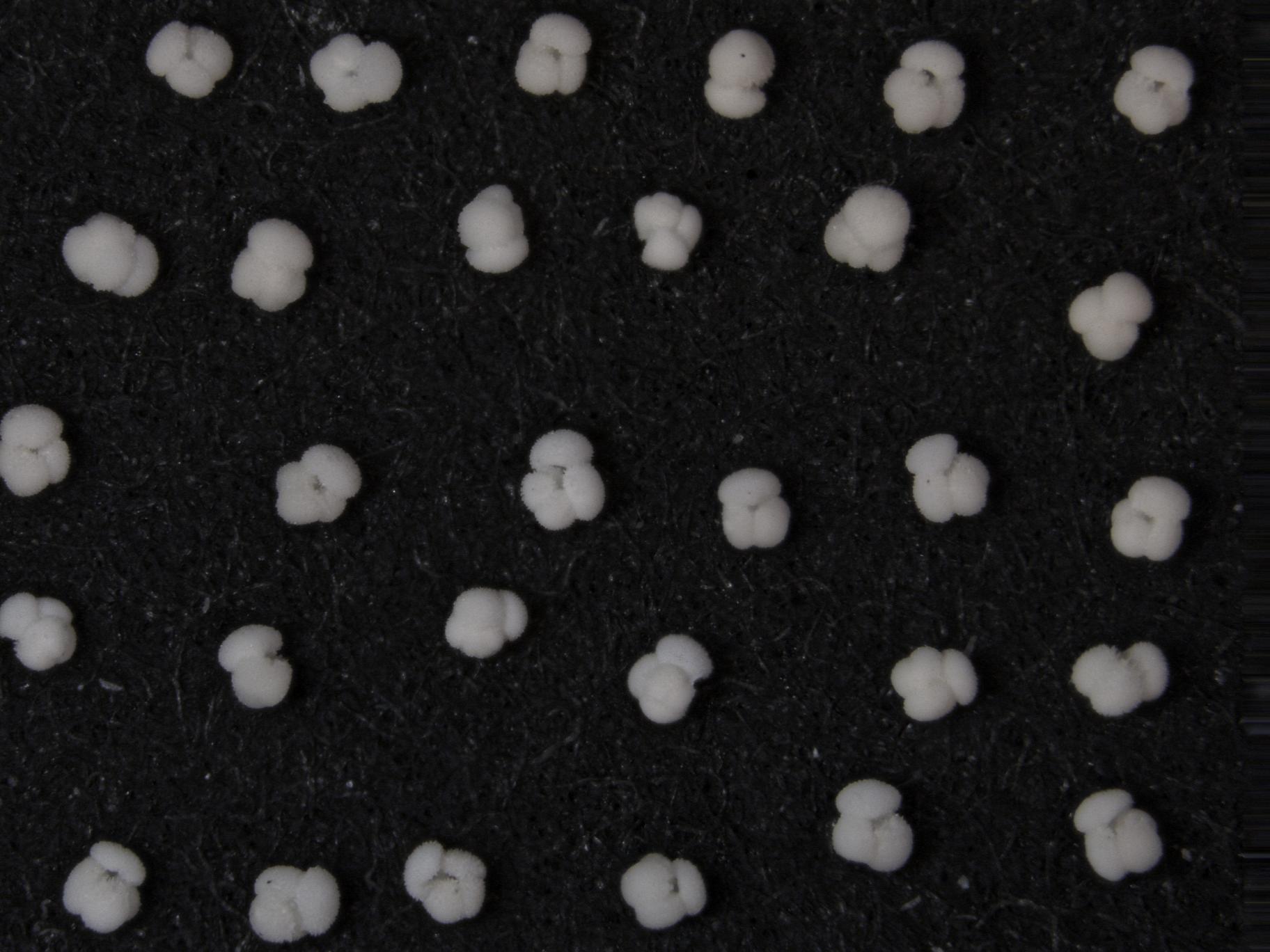

Supplement: S1 Data — (ZIP) [file pone.0267636.s001.zip › SDataImages/1209A-21H-3W_77-79_300_Aca1_2.0x_STACKED.jpg]

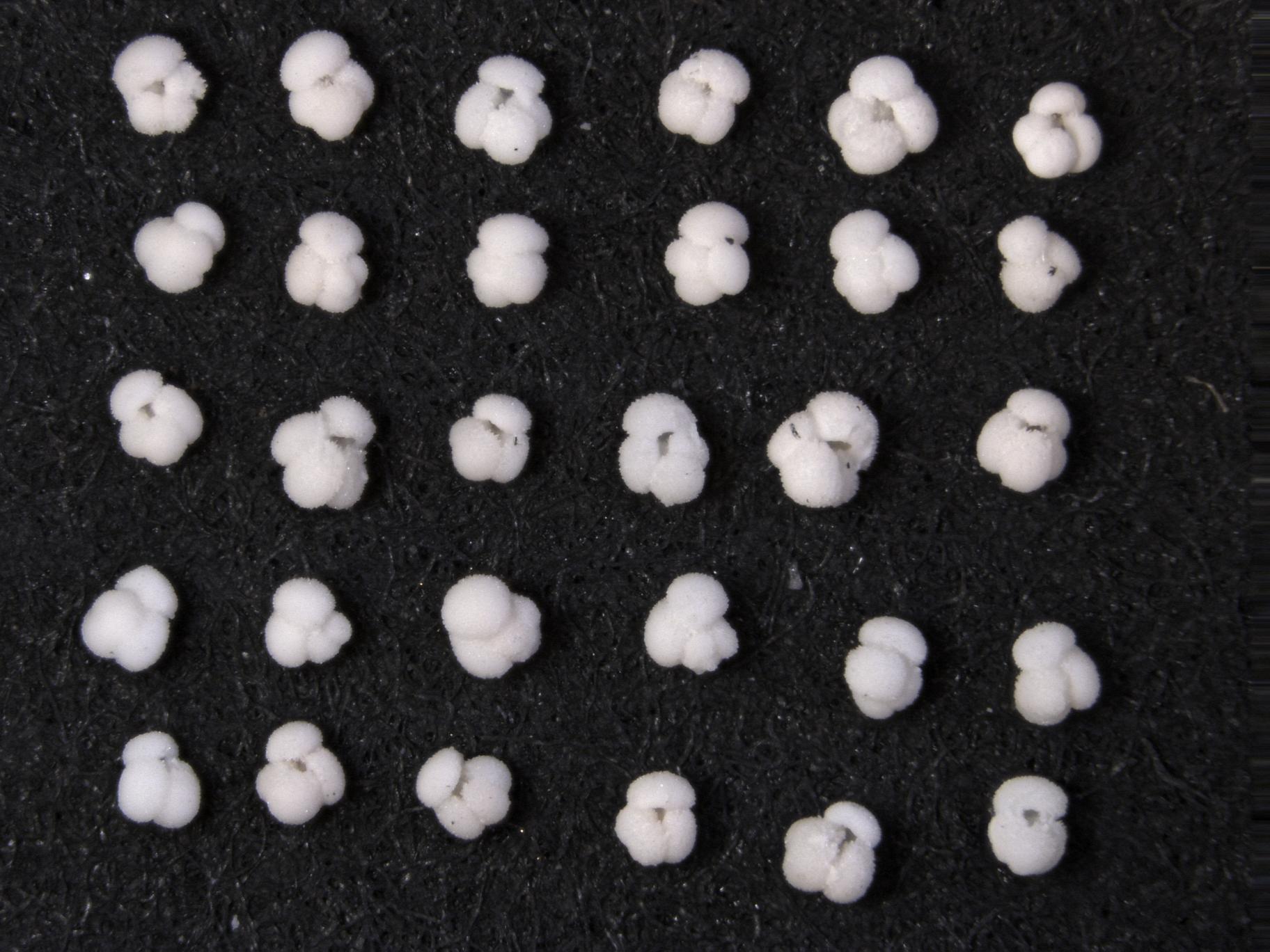

Supplement: S1 Data — (ZIP) [file pone.0267636.s001.zip › SDataImages/1209A-21H-3W_18-20_355_Aca1_2.0x_STACKED.jpg]

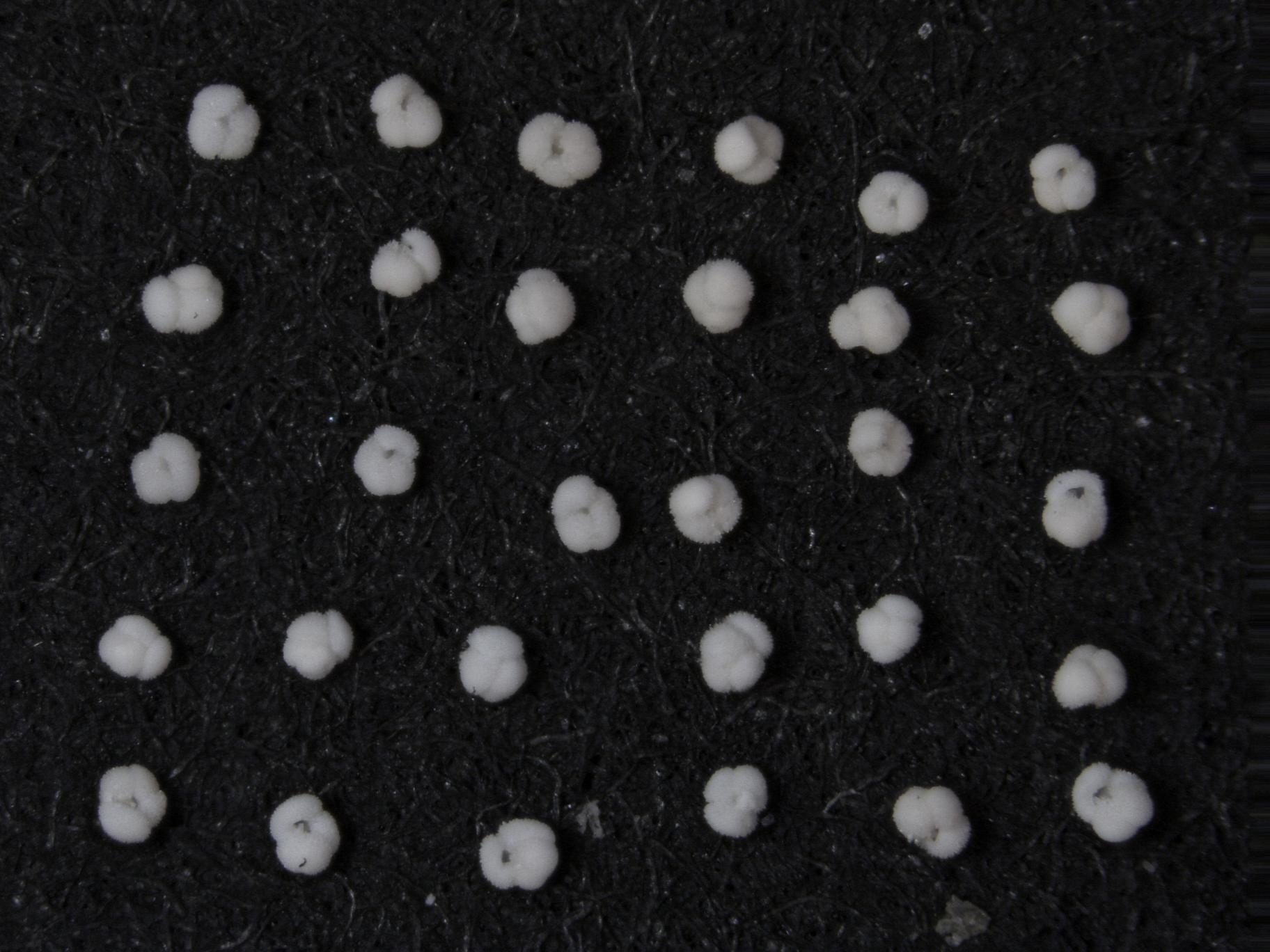

Supplement: S1 Data — (ZIP) [file pone.0267636.s001.zip › SDataImages/1209A-21H-3W_77-79_212_Aca1_2.5x_STACKED.jpg]

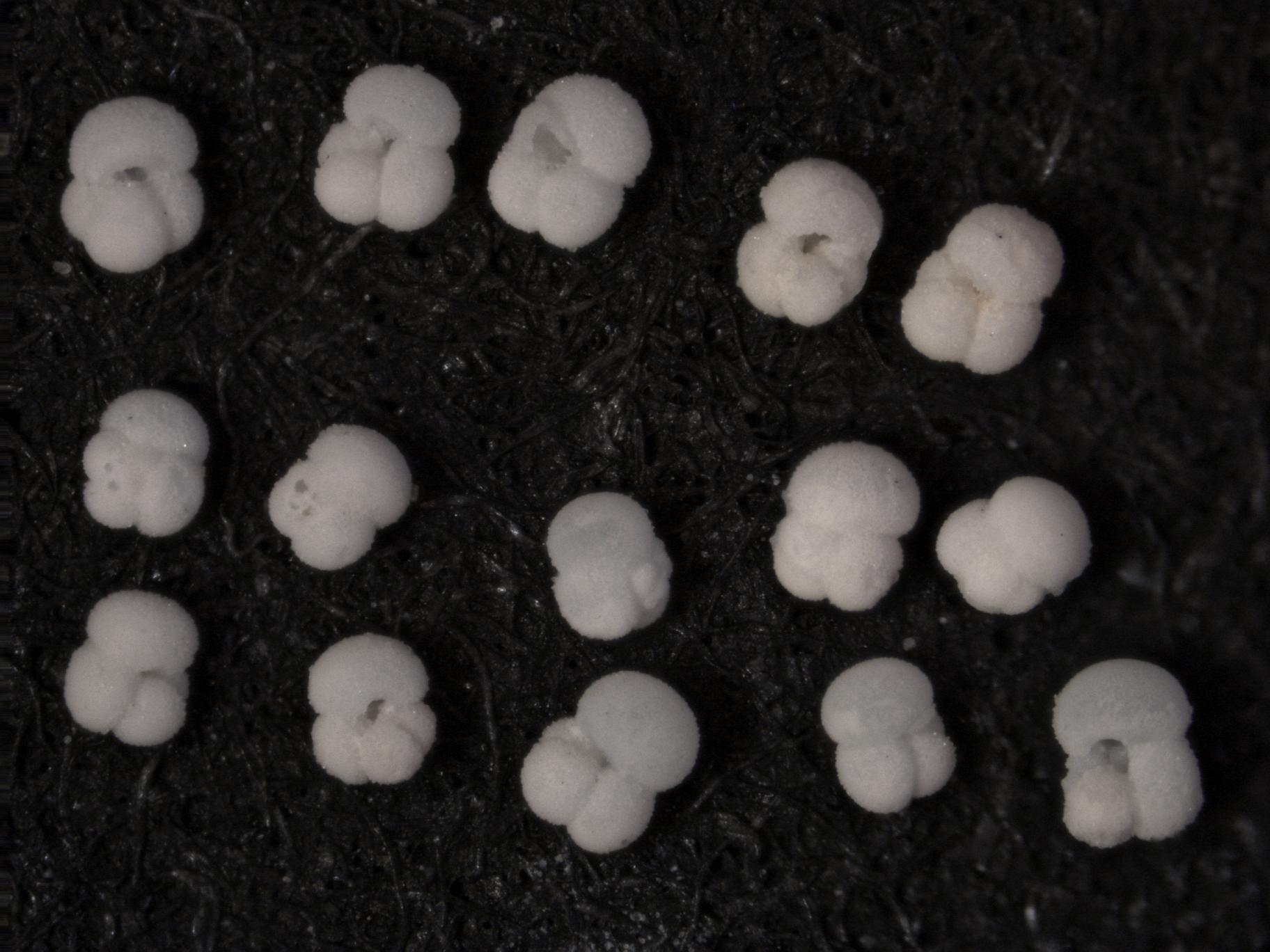

Supplement: S1 Data — (ZIP) [file pone.0267636.s001.zip › SDataImages/1209A-21H-3W_87-89_212_Sub2_5.0x_STACKED.jpg]

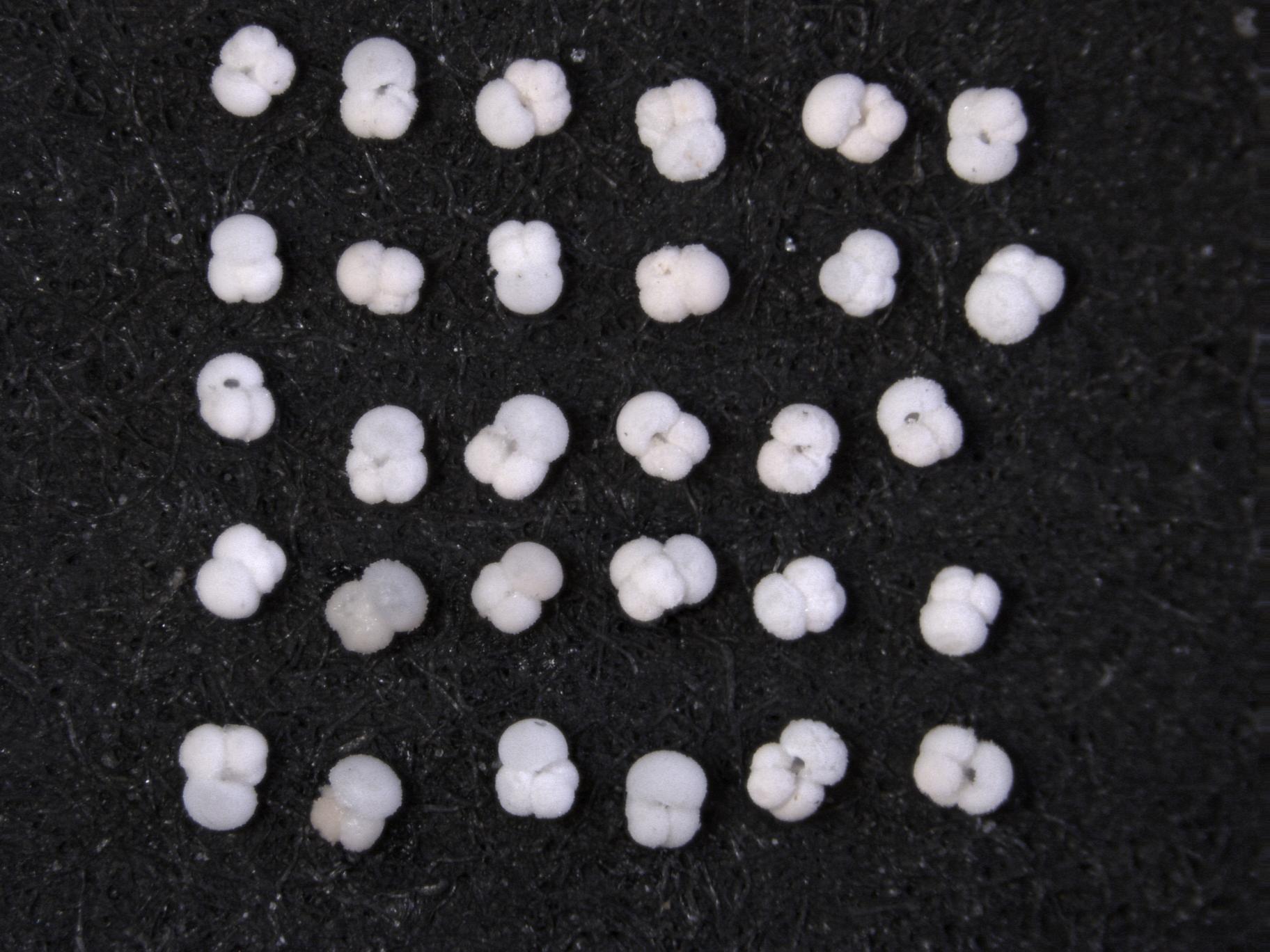

Supplement: S1 Data — (ZIP) [file pone.0267636.s001.zip › SDataImages/1209A-21H-3W_18-20_250_Sub1_2.5x_STACKED.jpg]

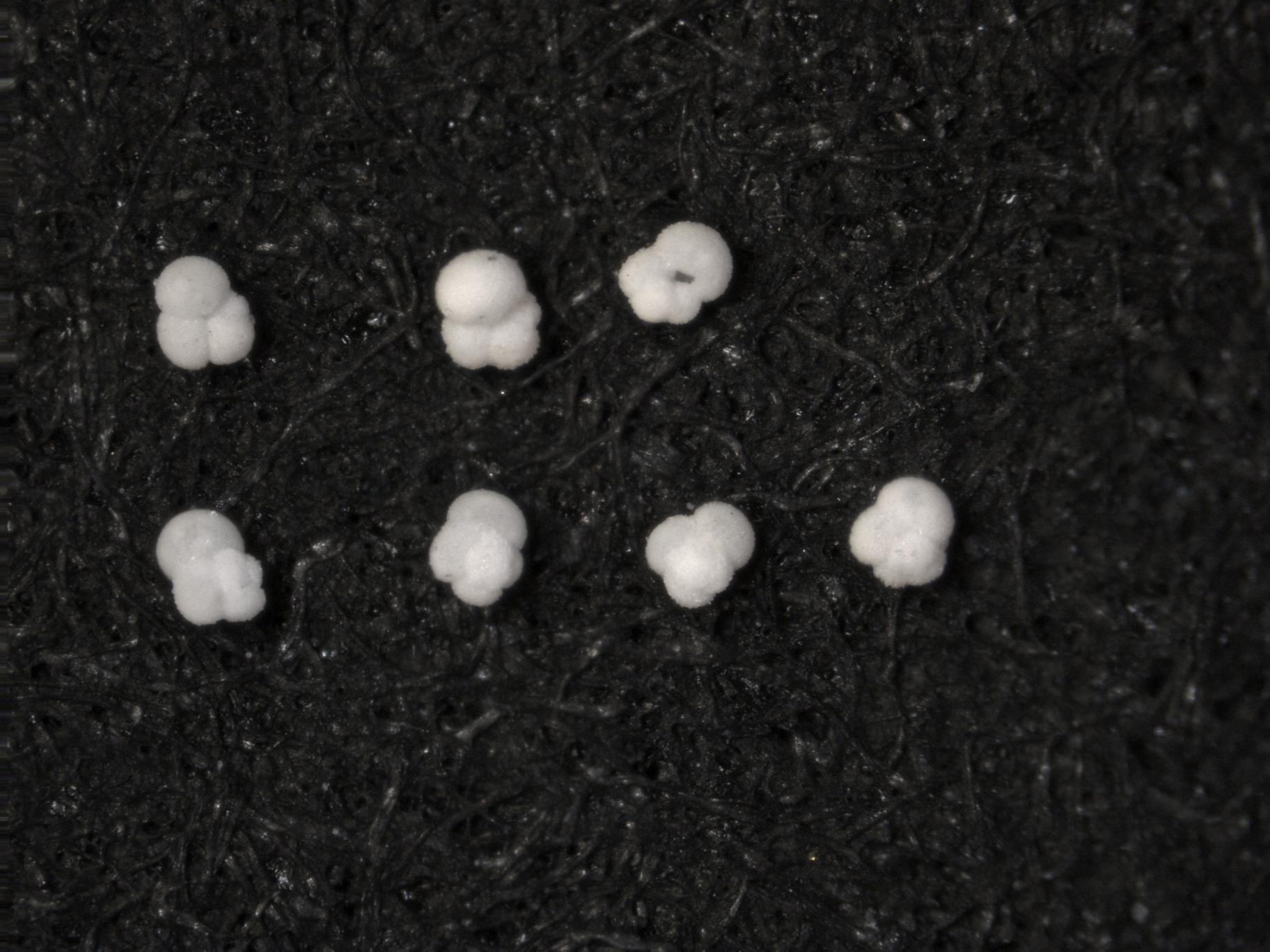

Supplement: S1 Data — (ZIP) [file pone.0267636.s001.zip › SDataImages/1209A-21H-3W_58-60_150_Sub2_5.0x_STACKED.jpg]

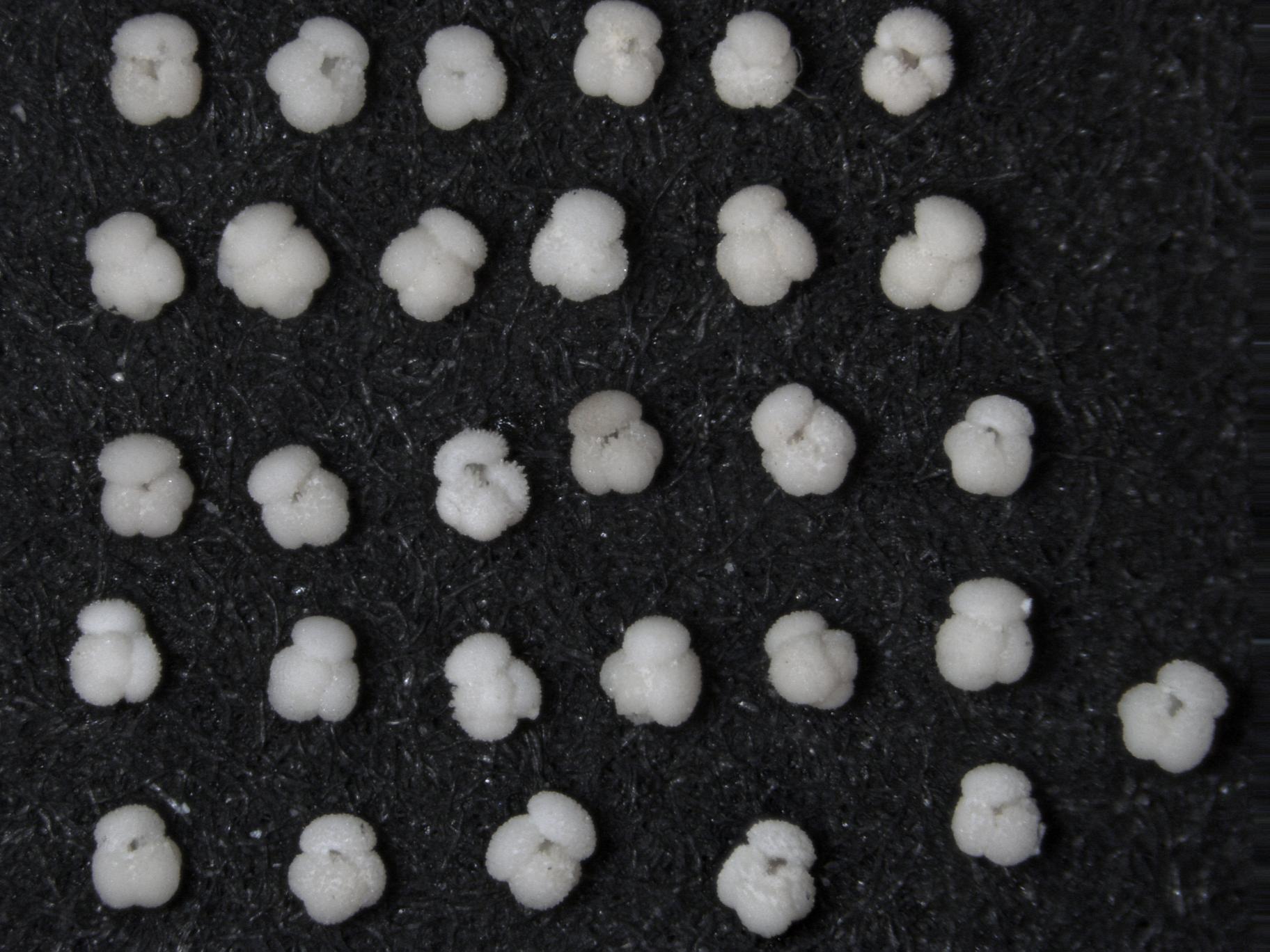

Supplement: S1 Data — (ZIP) [file pone.0267636.s001.zip › SDataImages/1209A-21H-3W_117-119_300_Aca1_2.5x_STACKED.jpg]

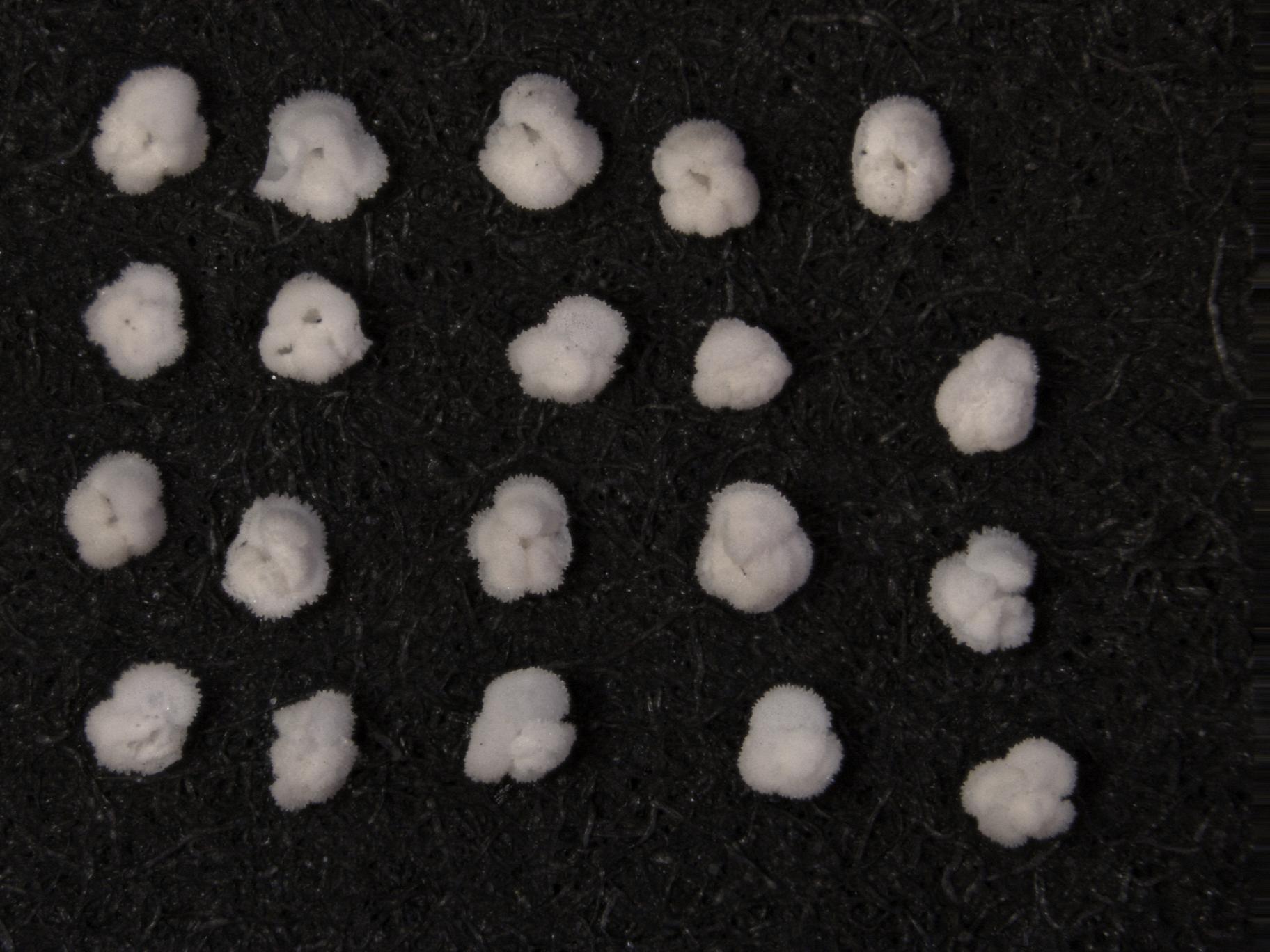

Supplement: S1 Data — (ZIP) [file pone.0267636.s001.zip › SDataImages/1209A-21H-3W_87-89_300_Mor1_2.5x_STACKED.jpg]

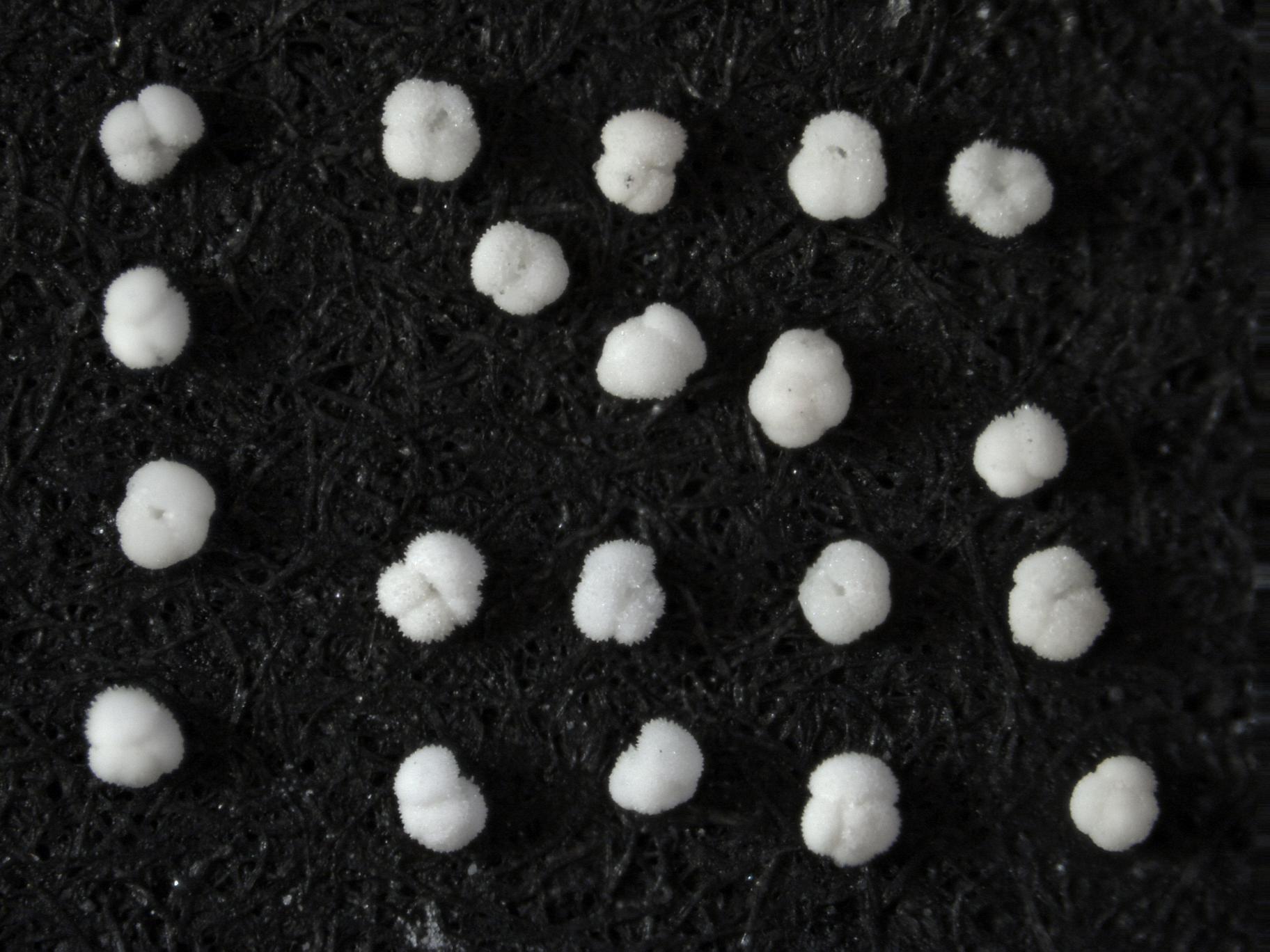

Supplement: S1 Data — (ZIP) [file pone.0267636.s001.zip › SDataImages/1209A-21H-3W_38-40_180_Aca2_4.0x_STACKED.jpg]

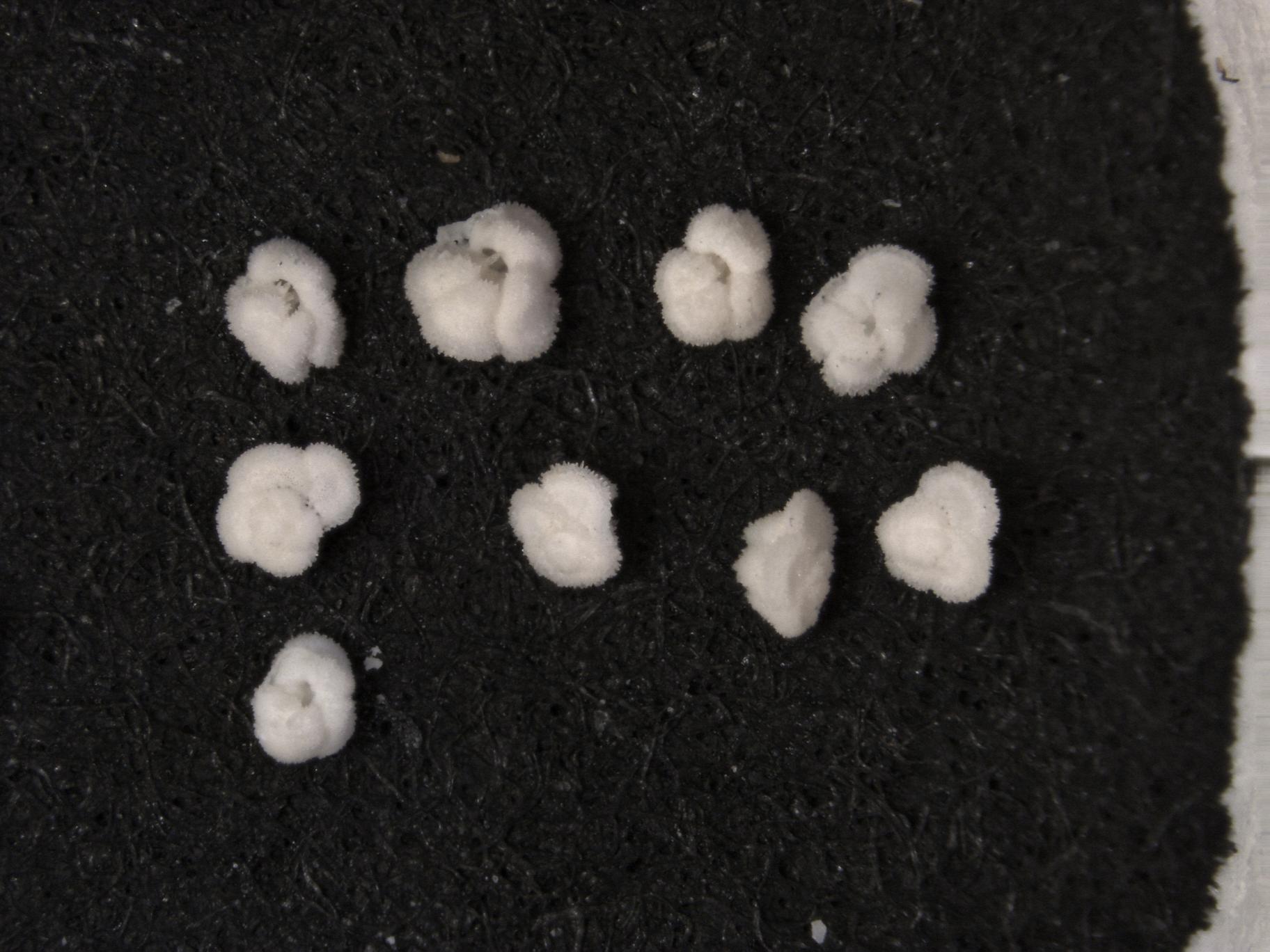

Supplement: S1 Data — (ZIP) [file pone.0267636.s001.zip › SDataImages/1209A-21H-3W_87-89_355_Mor3_2.5x_STACKED.jpg]

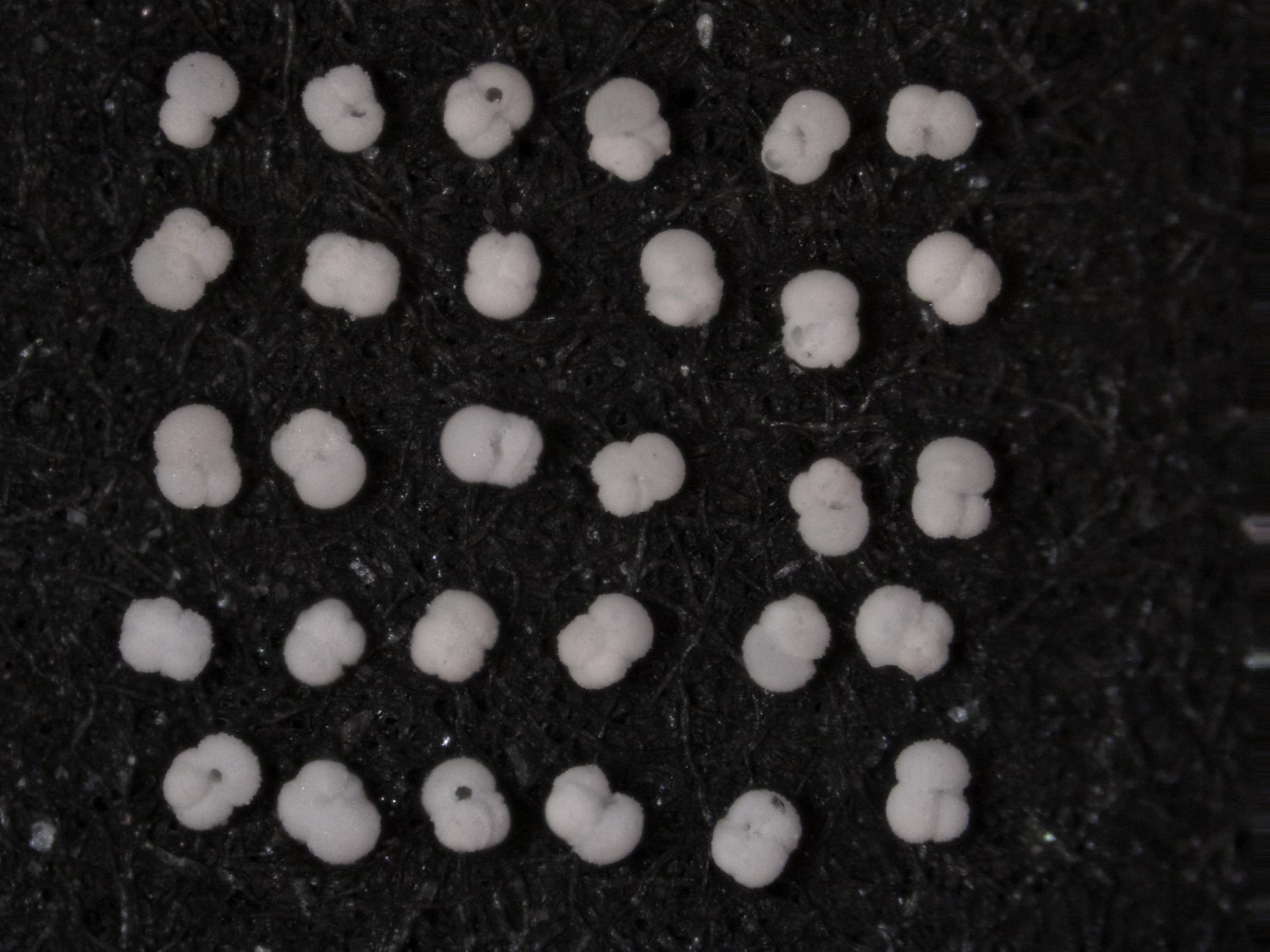

Supplement: S1 Data — (ZIP) [file pone.0267636.s001.zip › SDataImages/1209A-21H-2W_146-148_150_Sub1_4.0x_STACKED.jpg]

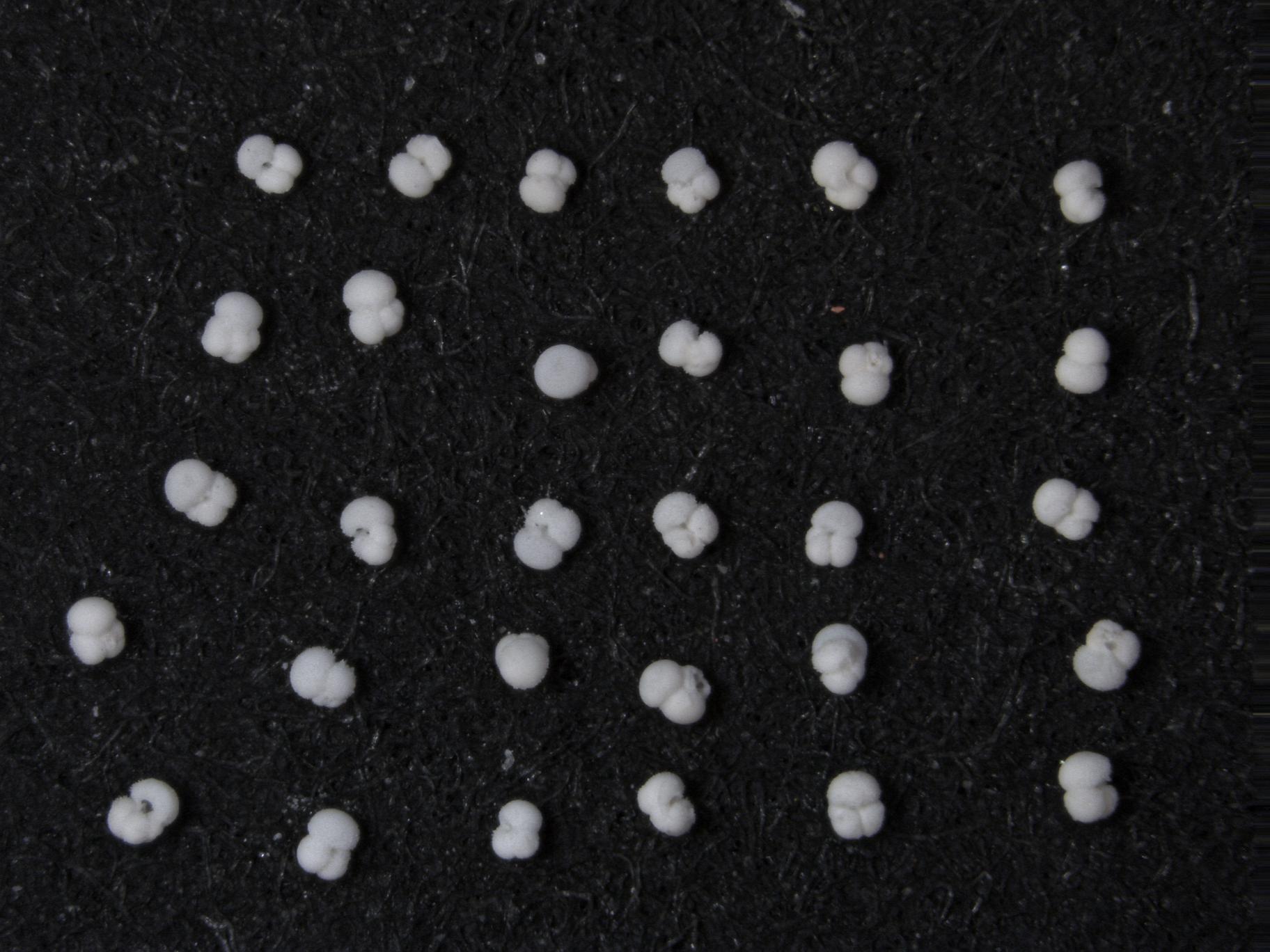

Supplement: S1 Data — (ZIP) [file pone.0267636.s001.zip › SDataImages/1209A-21H-3W_77-79_212_Sub1_2.0x_STACKED.jpg]

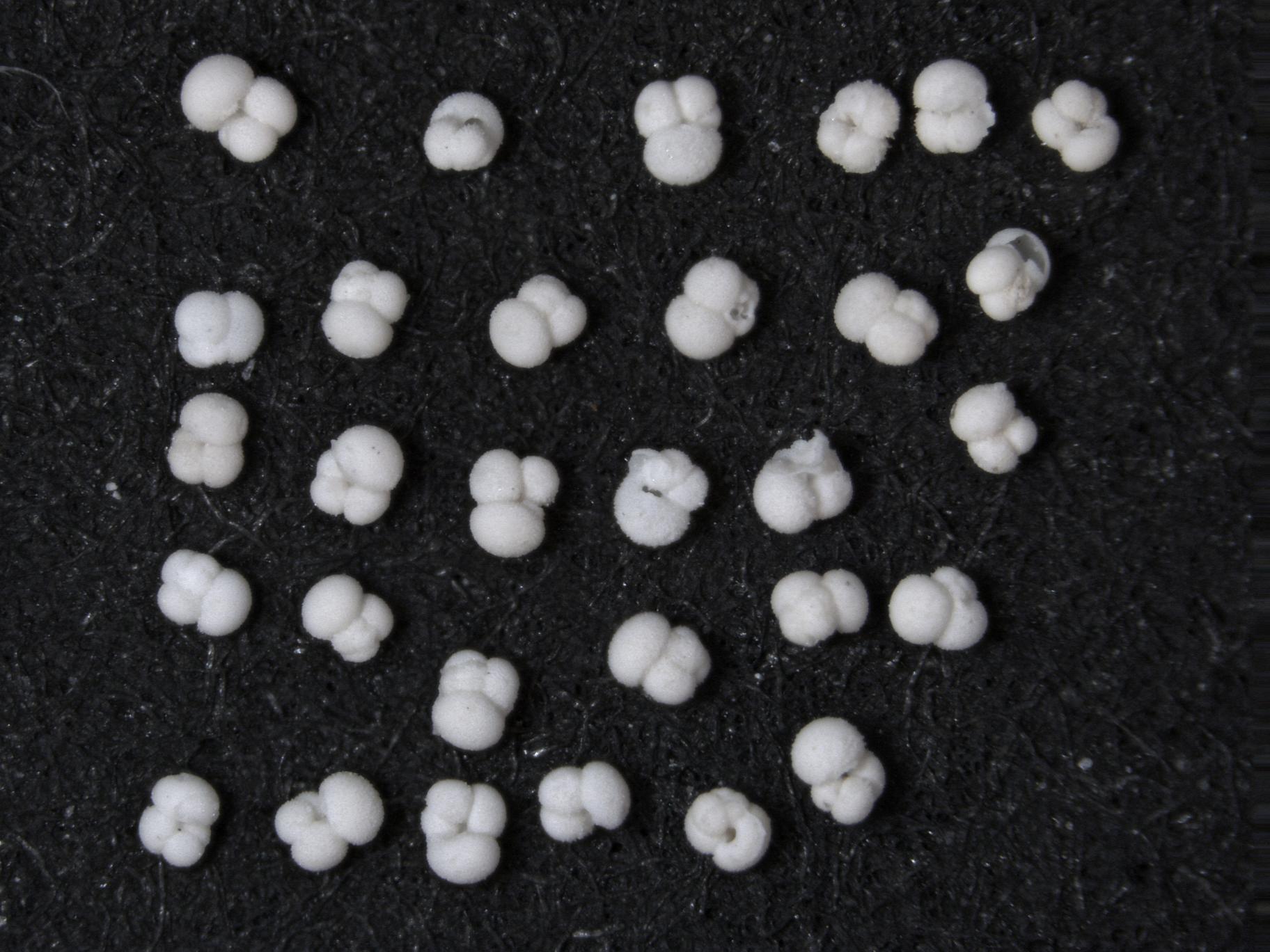

Supplement: S1 Data — (ZIP) [file pone.0267636.s001.zip › SDataImages/1209A-21H-3W_117-119_250_Sub1_2.5x_STACKED.jpg]

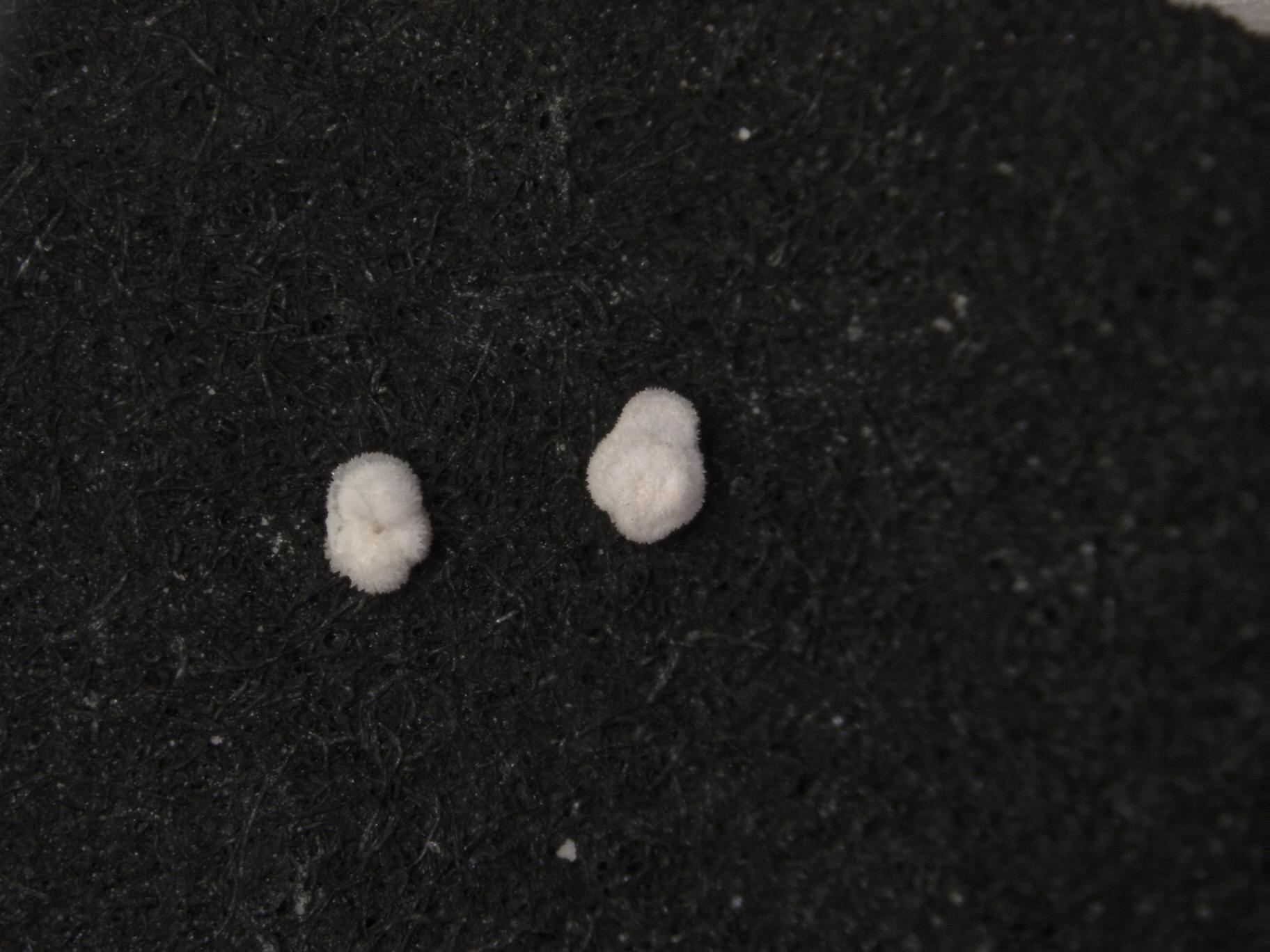

Supplement: S1 Data — (ZIP) [file pone.0267636.s001.zip › SDataImages/1209A-21H-3W_58-60_355_Mor3_2.5x_STACKED.jpg]

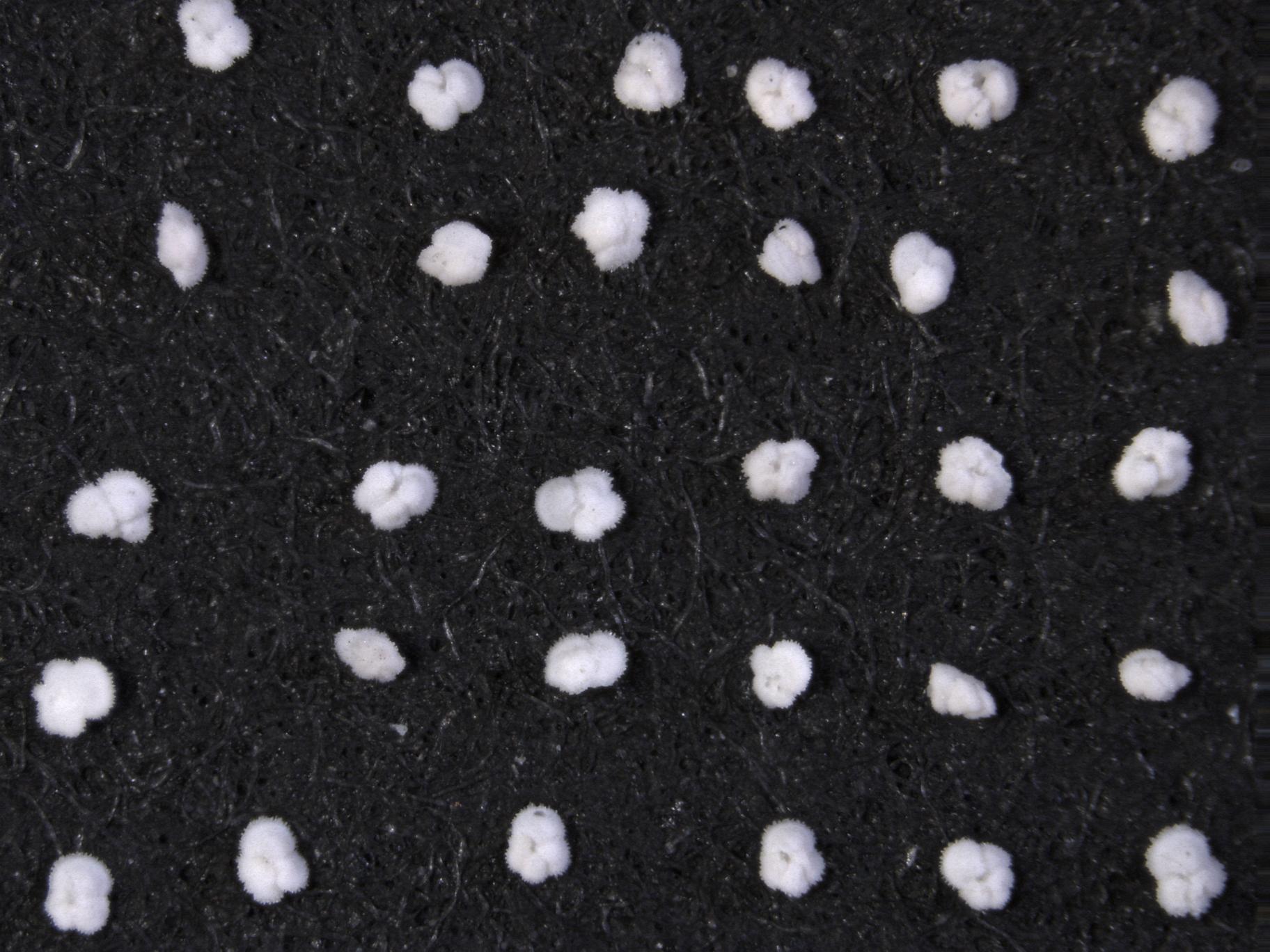

Supplement: S1 Data — (ZIP) [file pone.0267636.s001.zip › SDataImages/1209A-21H-3W_68-70_180_Mor1_2.5x_STACKED.jpg]

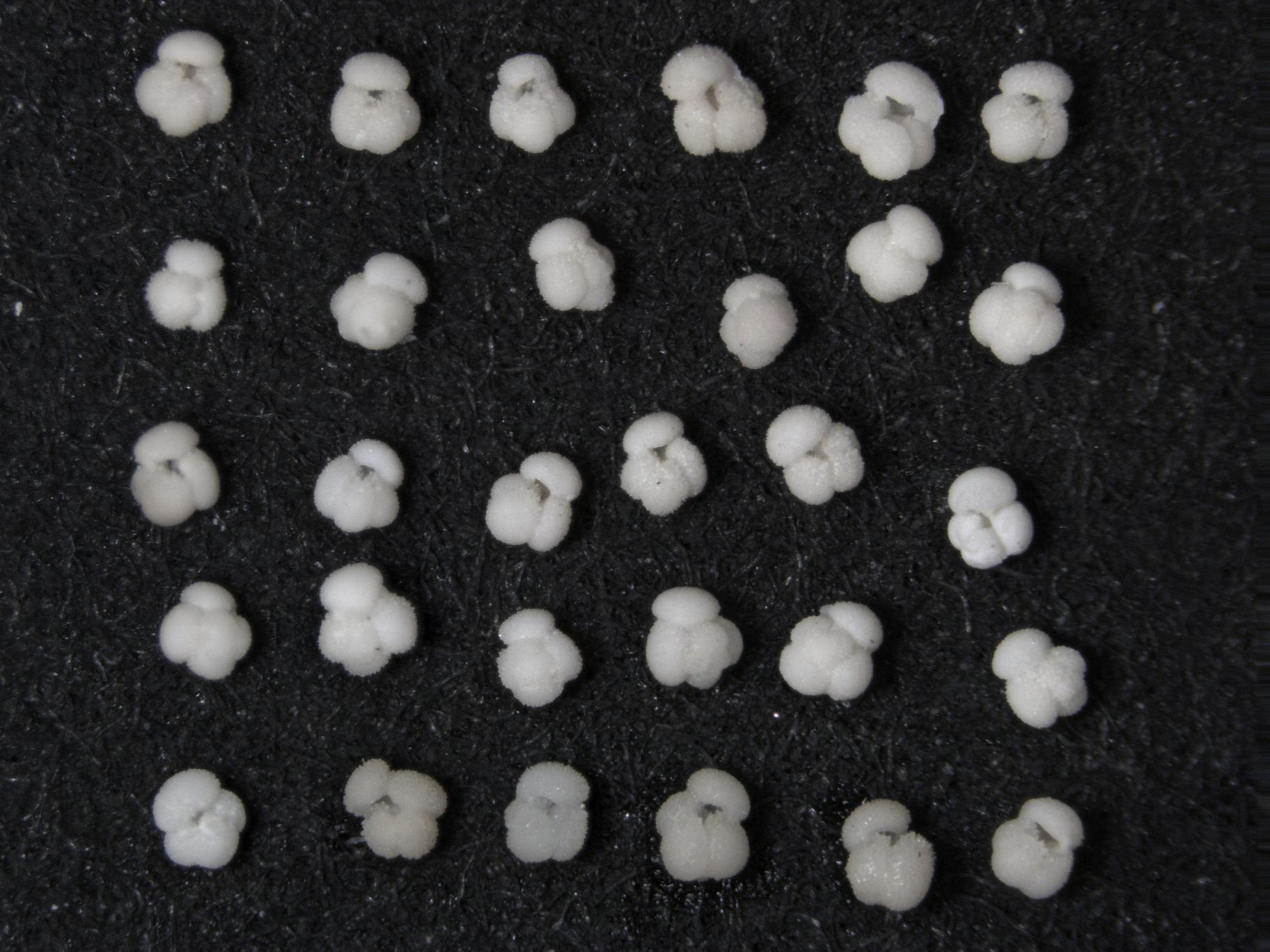

Supplement: S1 Data — (ZIP) [file pone.0267636.s001.zip › SDataImages/1209A-21H-3W_117-119_355_Aca1_2.0x_STACKED.jpg]

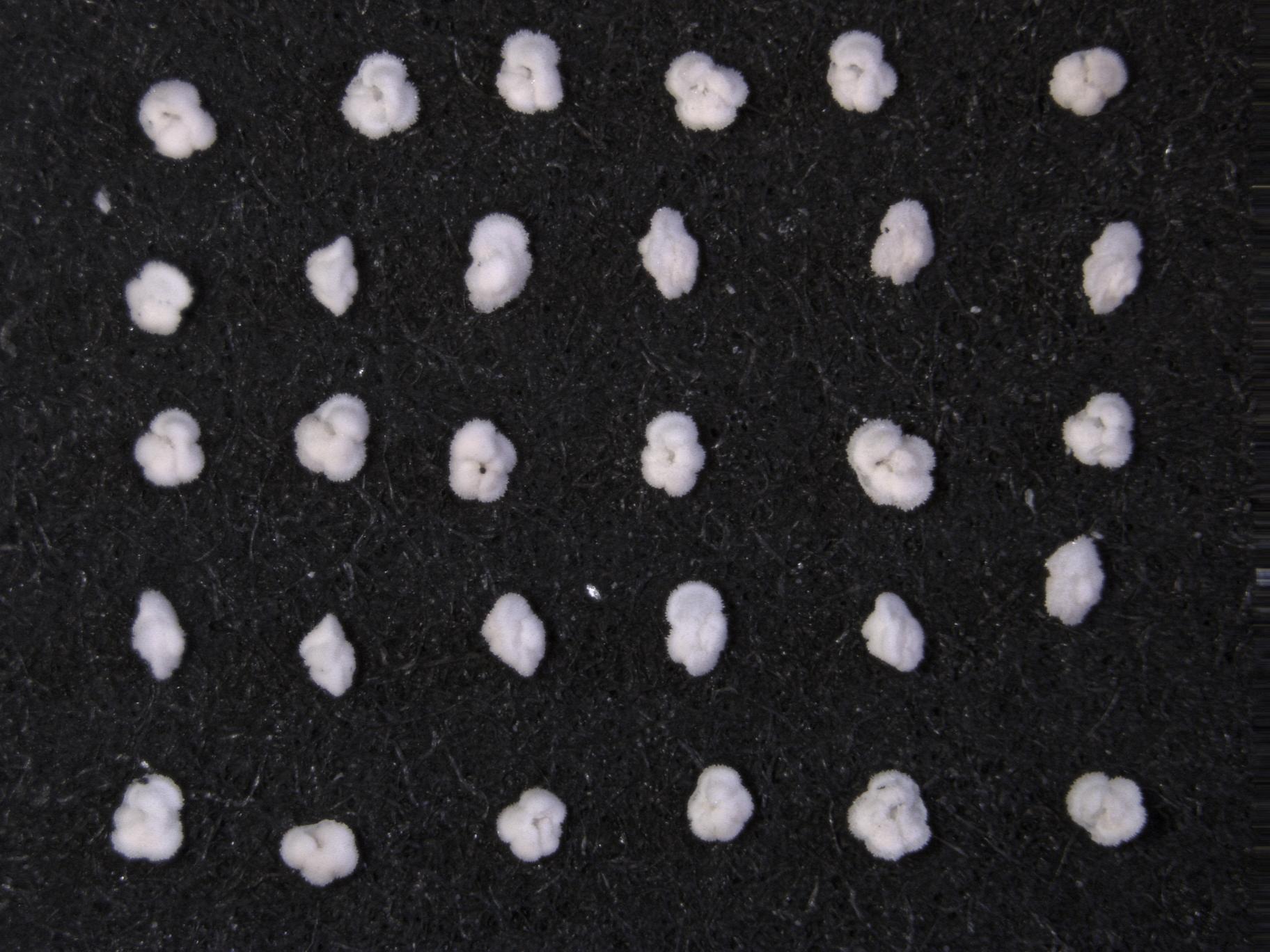

Supplement: S1 Data — (ZIP) [file pone.0267636.s001.zip › SDataImages/1209A-21H-3W_68-70_300_Mor1_1.6x_STACKED.jpg]

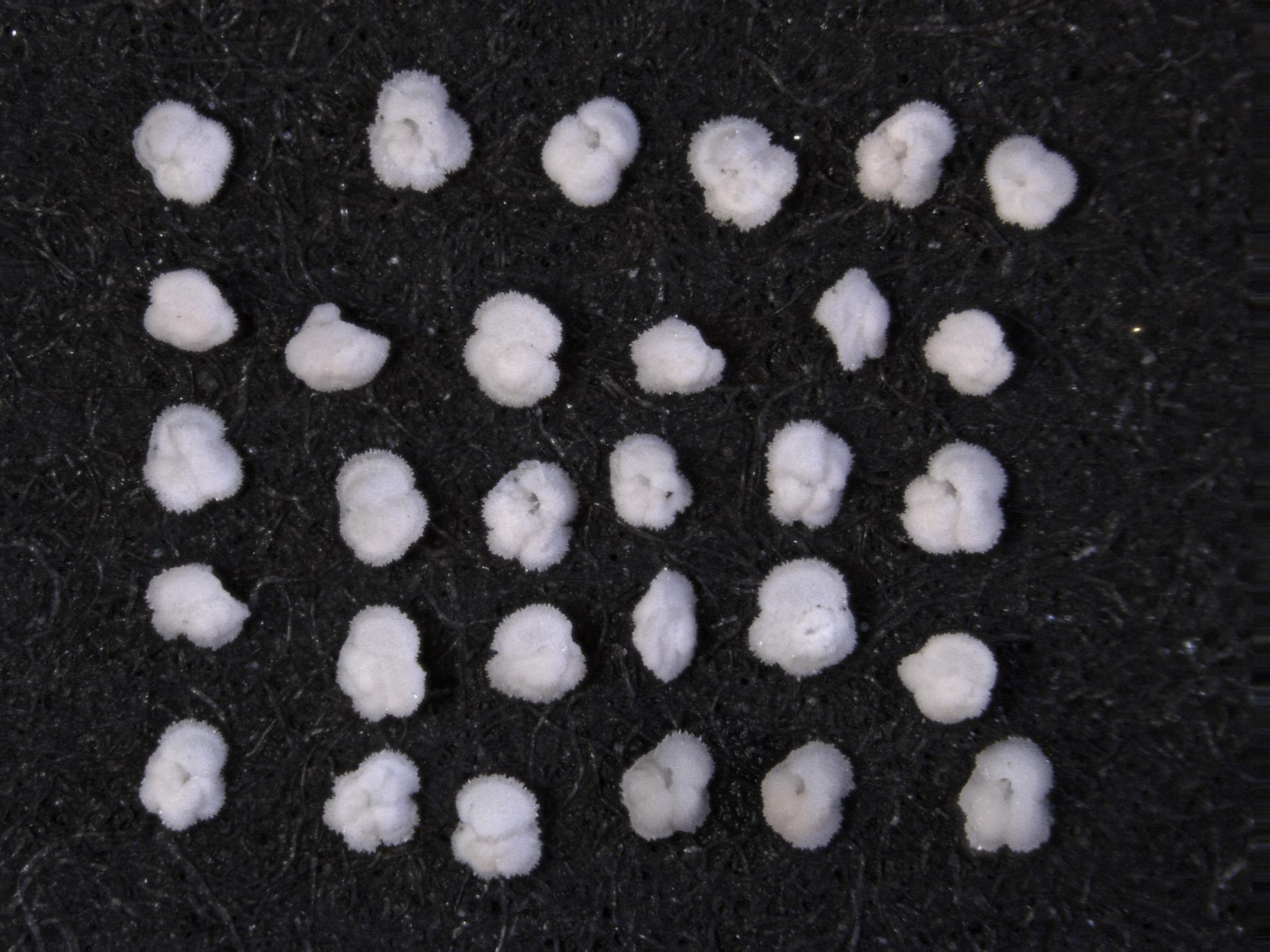

Supplement: S1 Data — (ZIP) [file pone.0267636.s001.zip › SDataImages/1209A-21H-2W_146-148_250_Mor1_2.5x_STACKED.jpg]

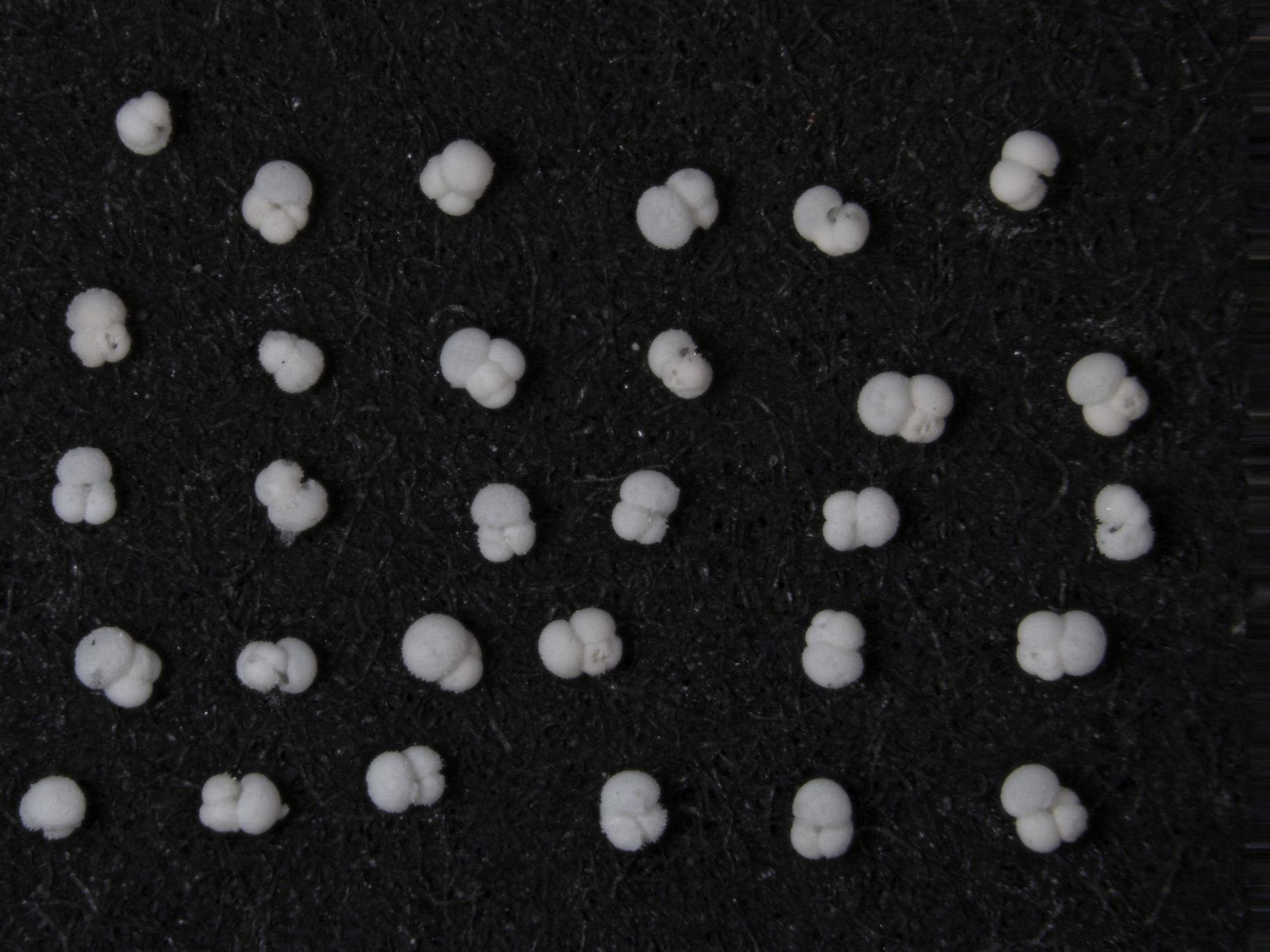

Supplement: S1 Data — (ZIP) [file pone.0267636.s001.zip › SDataImages/1209A-21H-3W_77-79_250_Sub1_2.0x_STACKED.jpg]

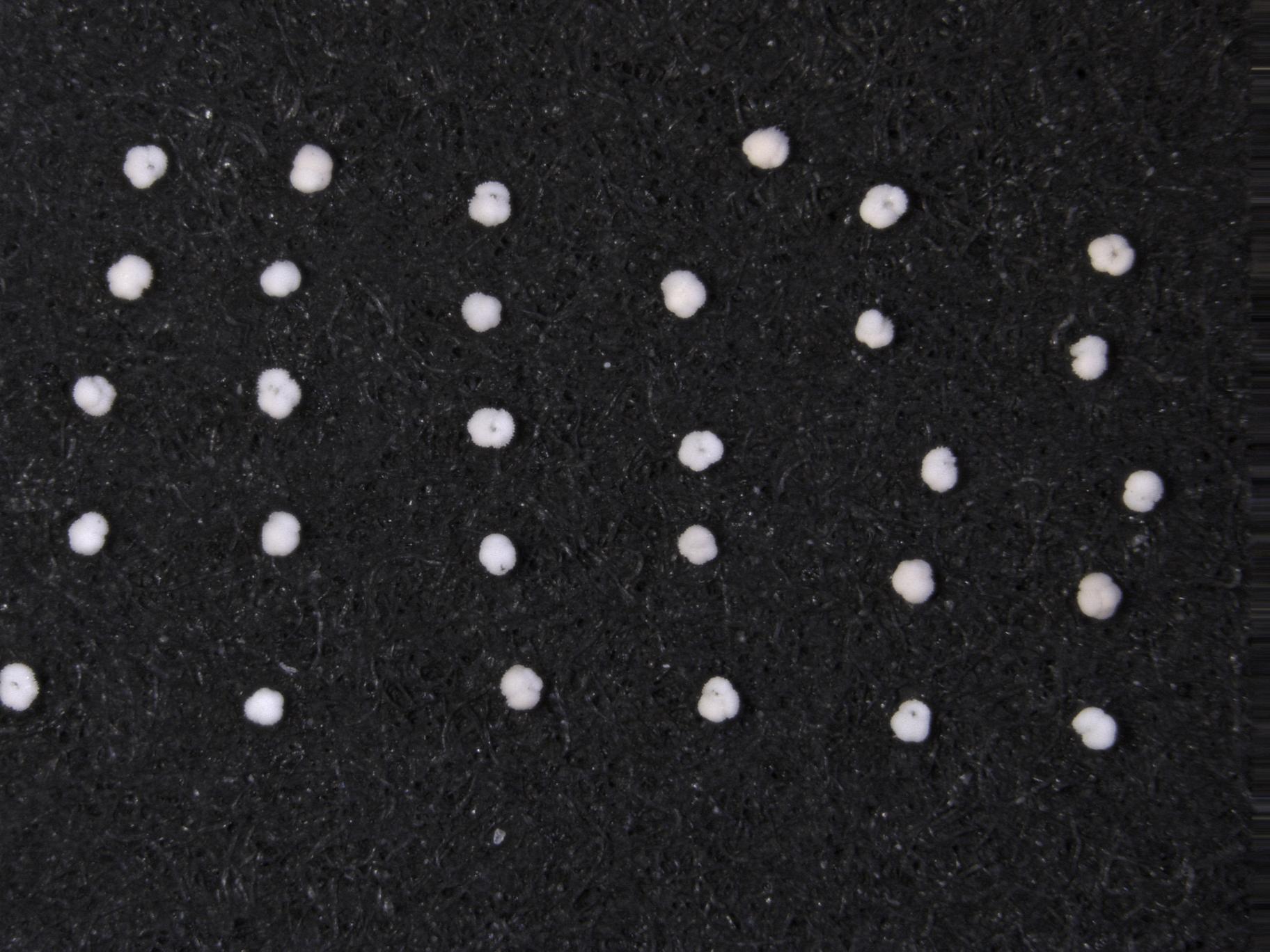

Supplement: S1 Data — (ZIP) [file pone.0267636.s001.zip › SDataImages/1209A-21H-3W_117-119_150_Aca1_2.0x_STACKED.jpg]

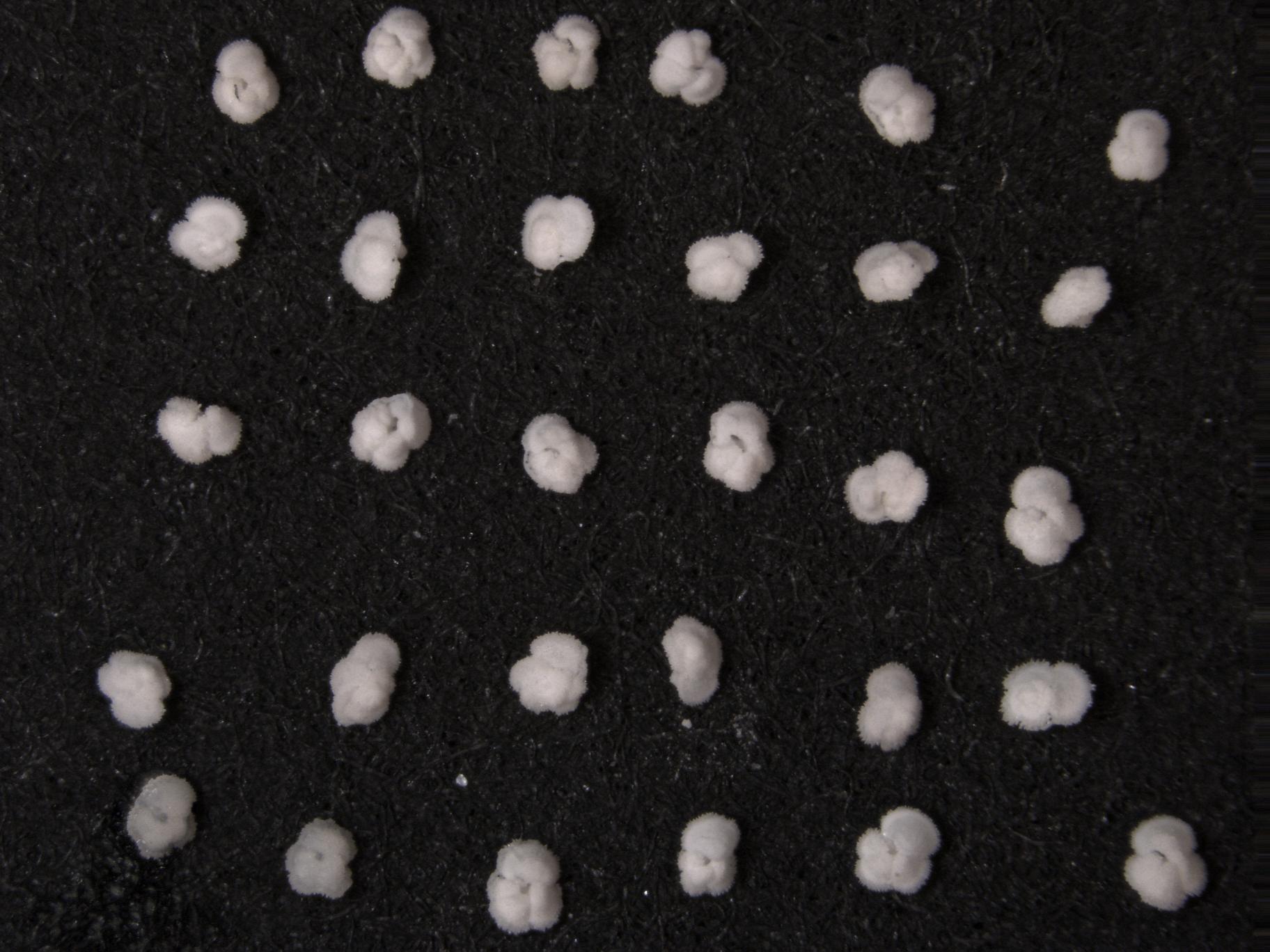

Supplement: S1 Data — (ZIP) [file pone.0267636.s001.zip › SDataImages/1209A-21H-3W_47-49_300_Mor1_1.6x_STACKED.jpg]

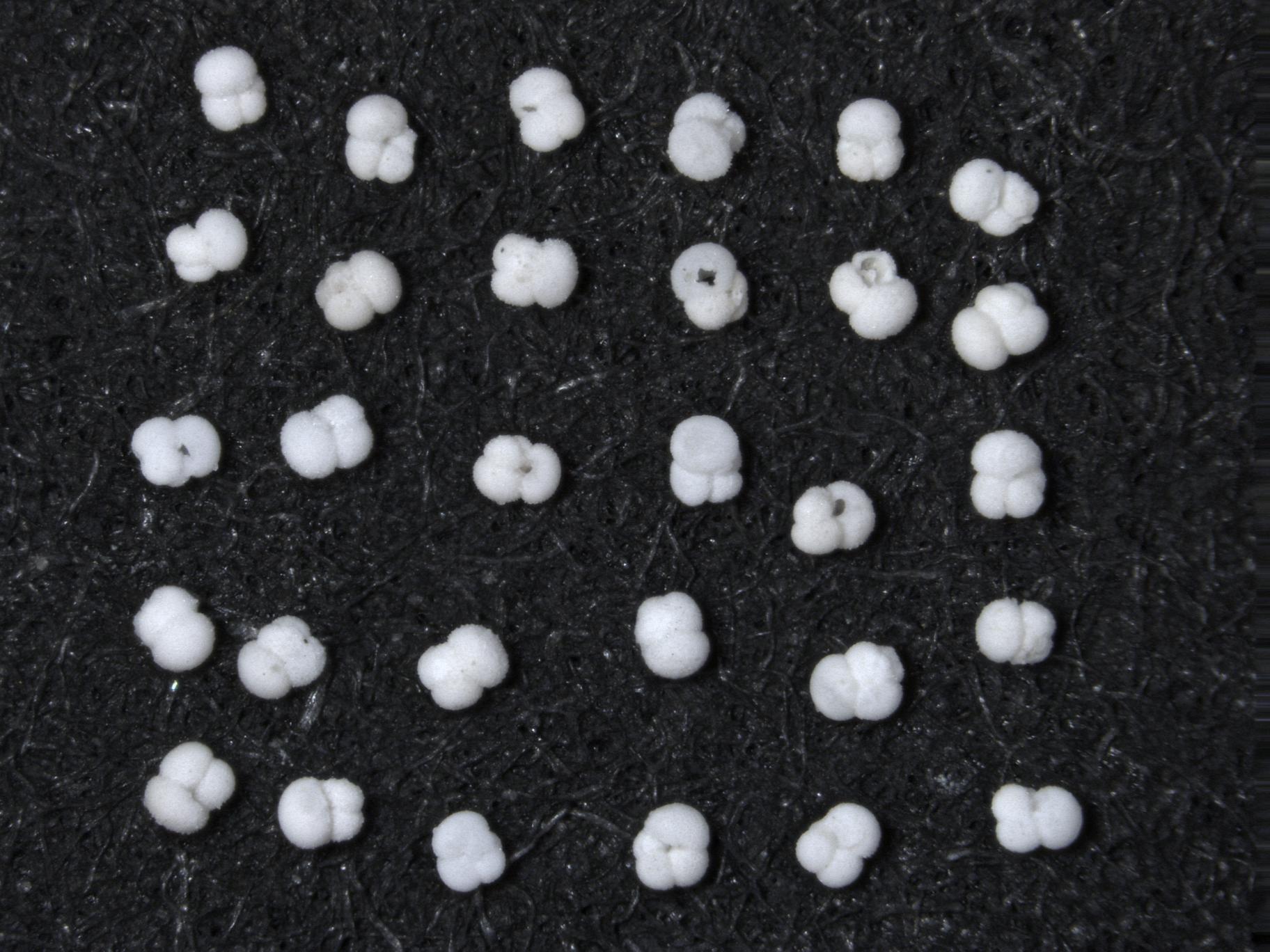

Supplement: S1 Data — (ZIP) [file pone.0267636.s001.zip › SDataImages/1209A-21H-3W_87-89_180_Sub1_3.2x_STACKED.jpg]

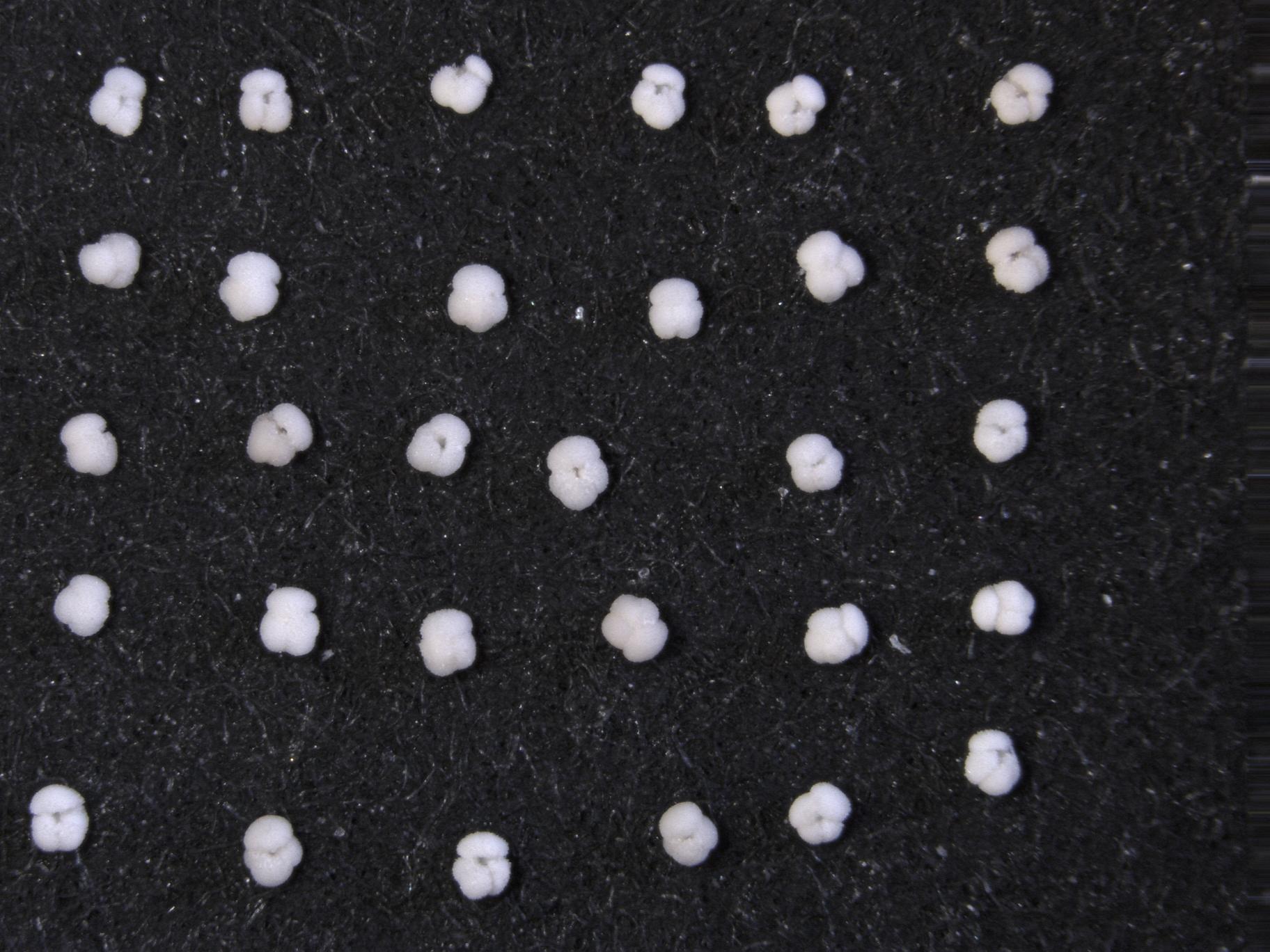

Supplement: S1 Data — (ZIP) [file pone.0267636.s001.zip › SDataImages/1209A-21H-2W_86-88_300_Aca1_1.6x_STACKED.jpg]

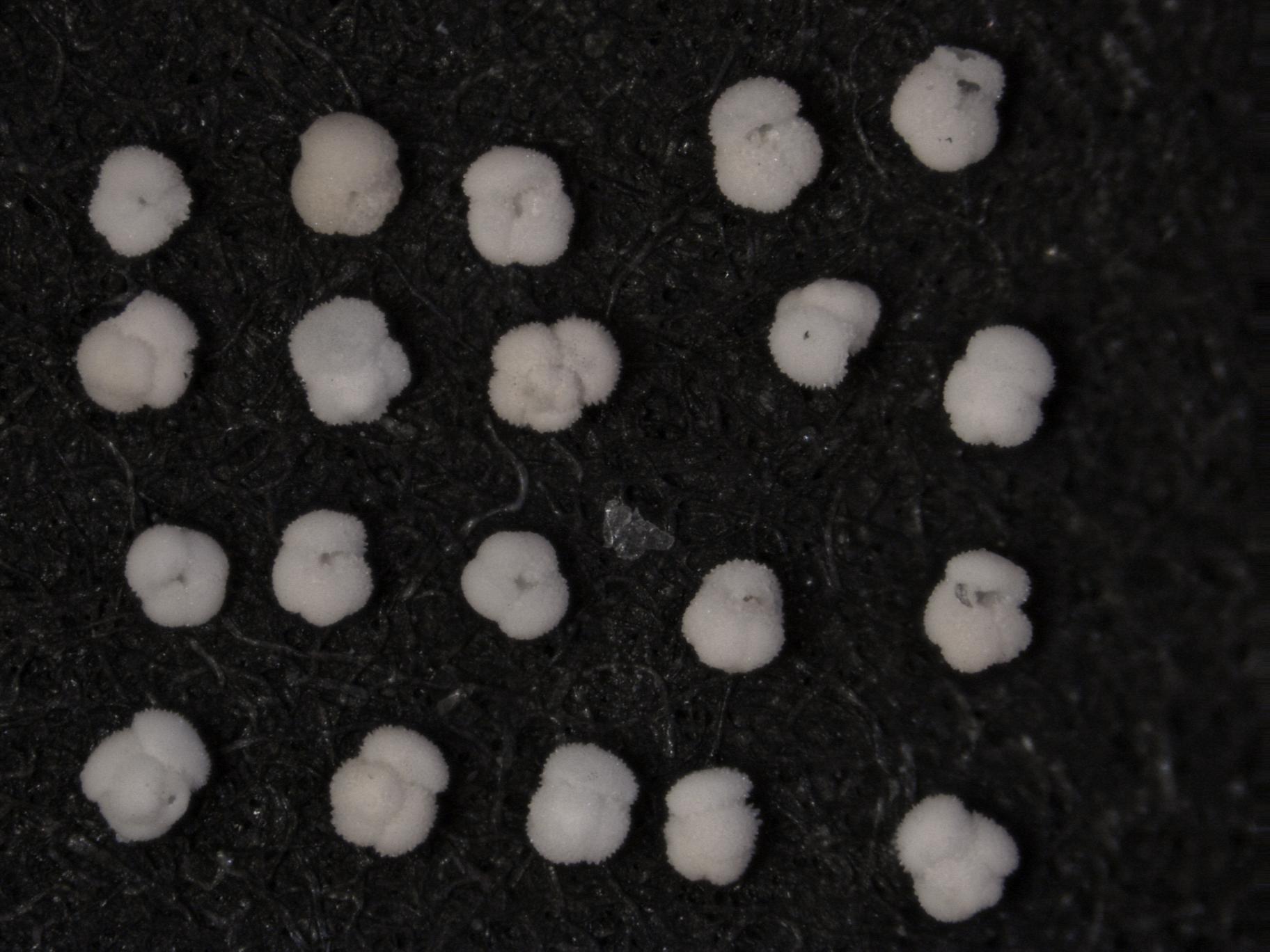

Supplement: S1 Data — (ZIP) [file pone.0267636.s001.zip › SDataImages/1209A-21H-3W_87-89_212_Aca1_4.0x_STACKED.jpg]

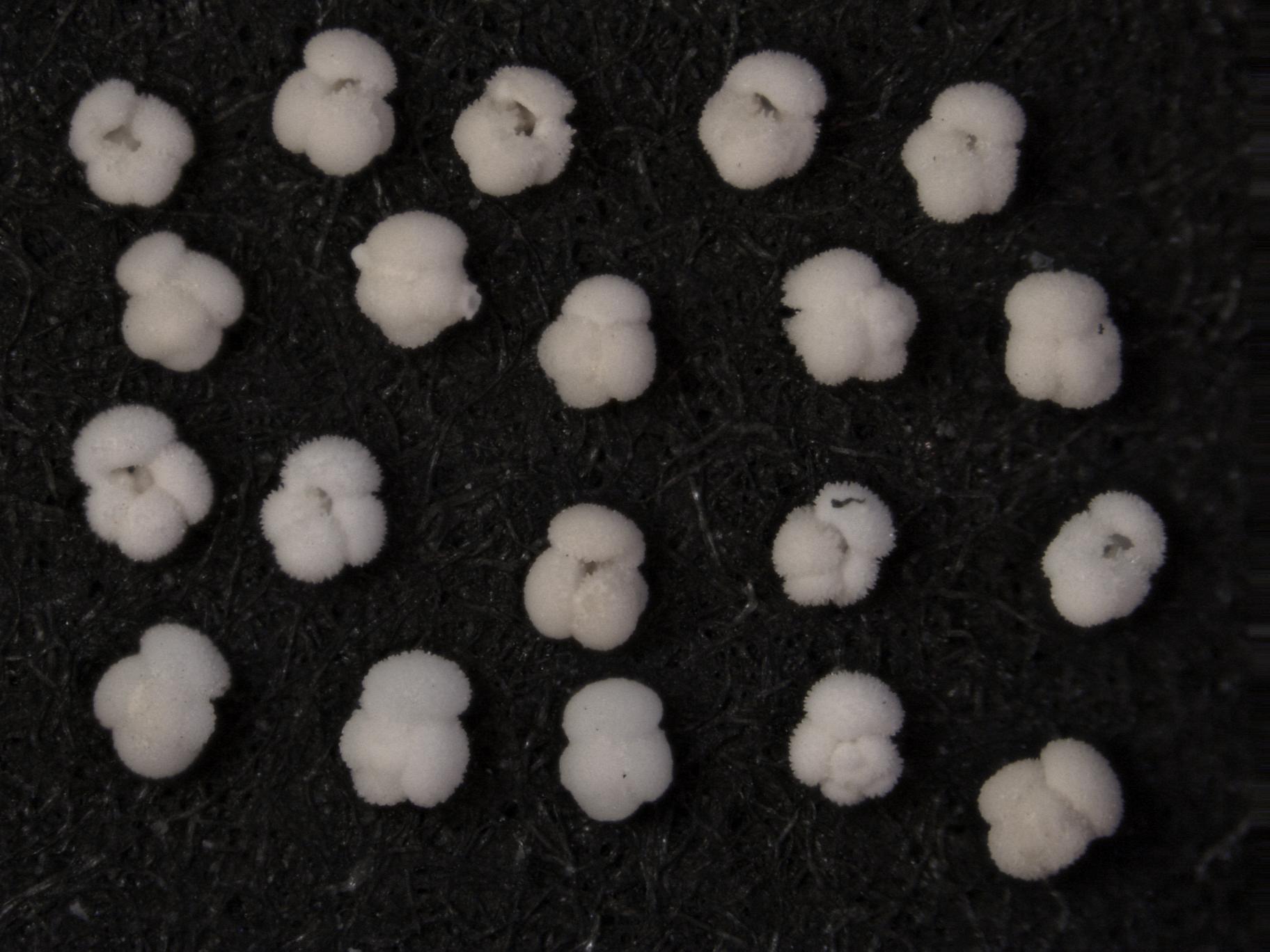

Supplement: S1 Data — (ZIP) [file pone.0267636.s001.zip › SDataImages/1209A-21H-3W_87-89_300_Aca1_3.2x_STACKED.jpg]

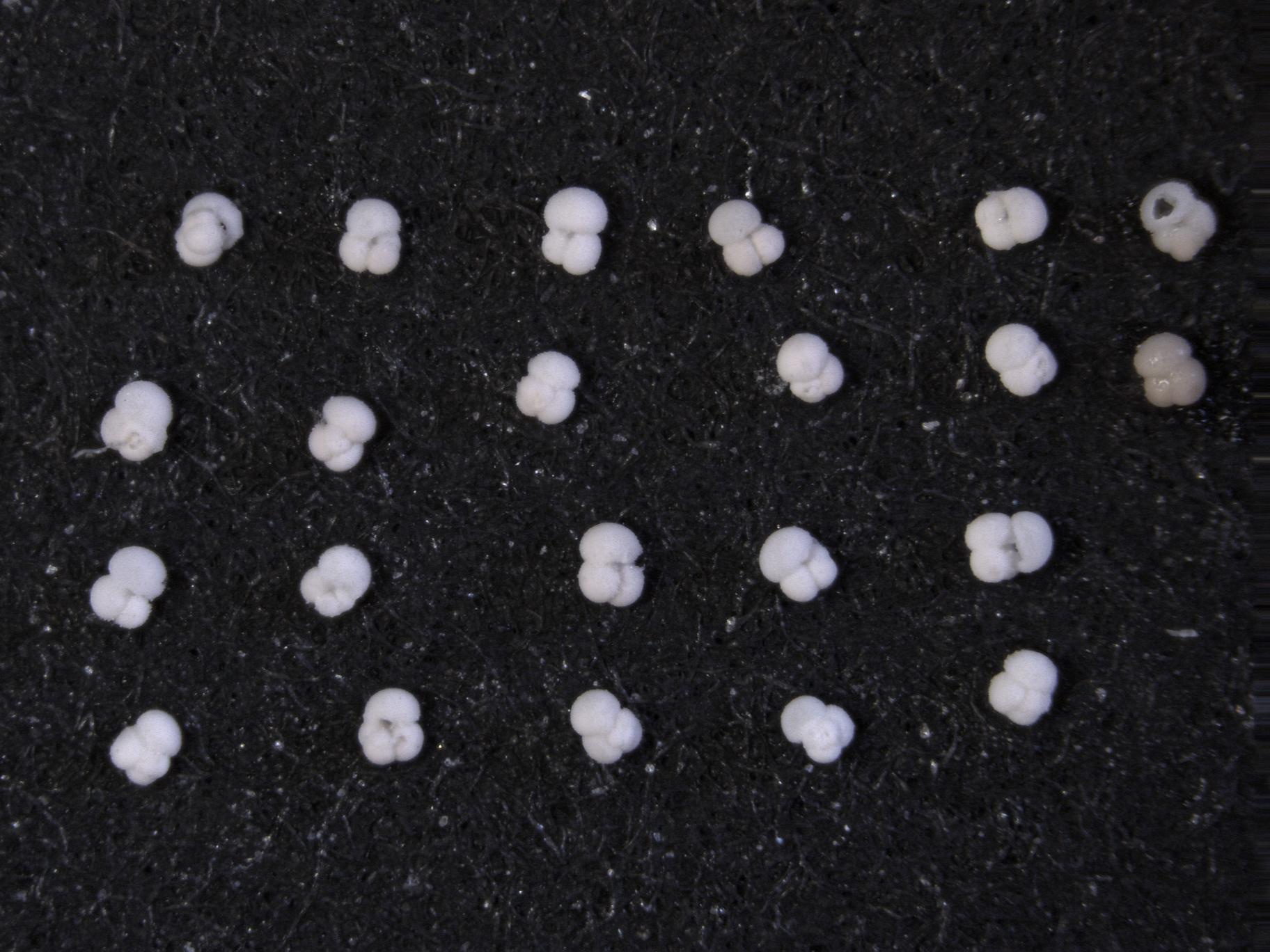

Supplement: S1 Data — (ZIP) [file pone.0267636.s001.zip › SDataImages/1209A-21H-2W_86-88_250_Sub1_2.0x_STACKED.jpg]

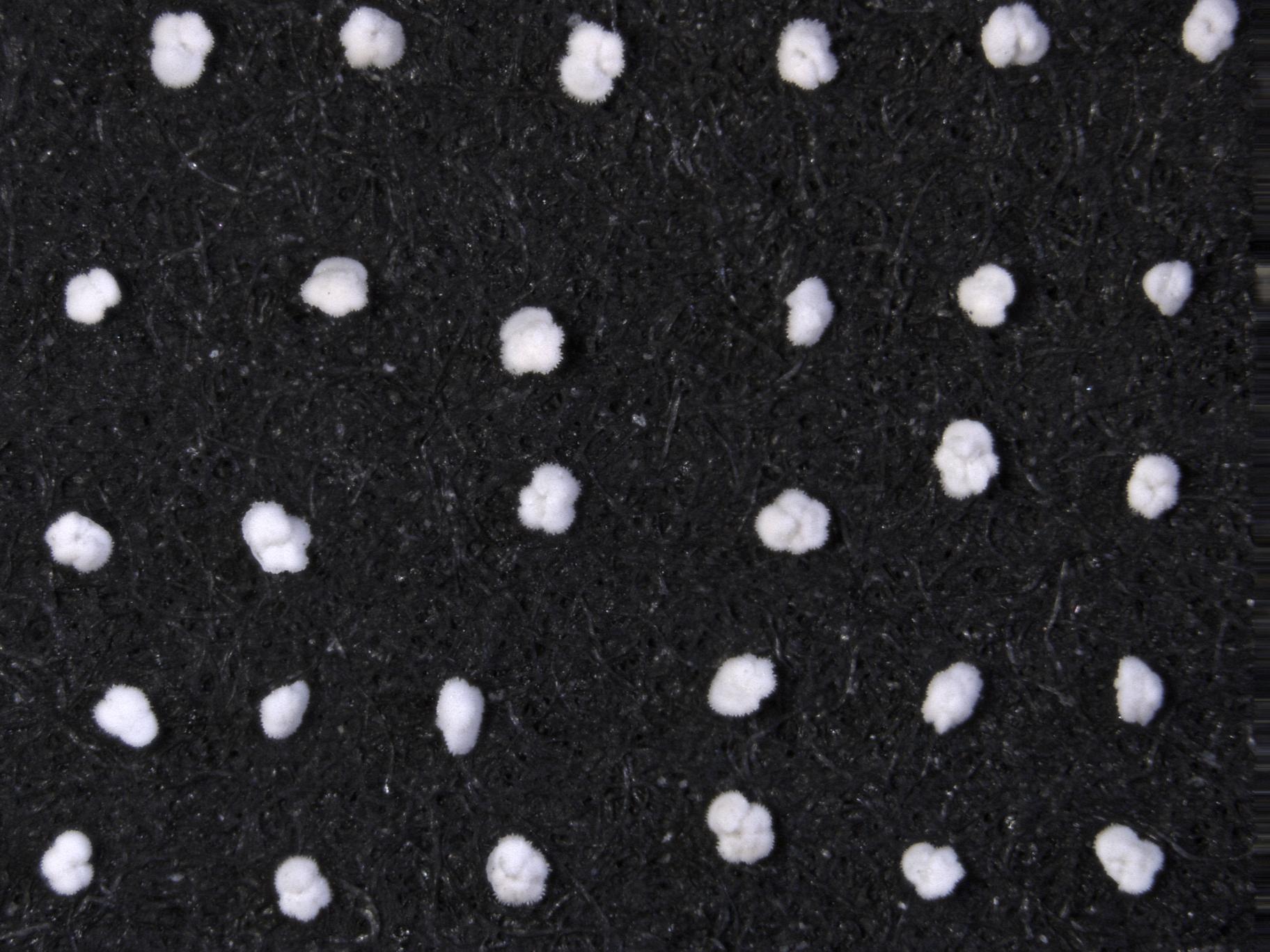

Supplement: S1 Data — (ZIP) [file pone.0267636.s001.zip › SDataImages/1209A-21H-3W_68-70_150_Mor1_2.5x_STACKED.jpg]

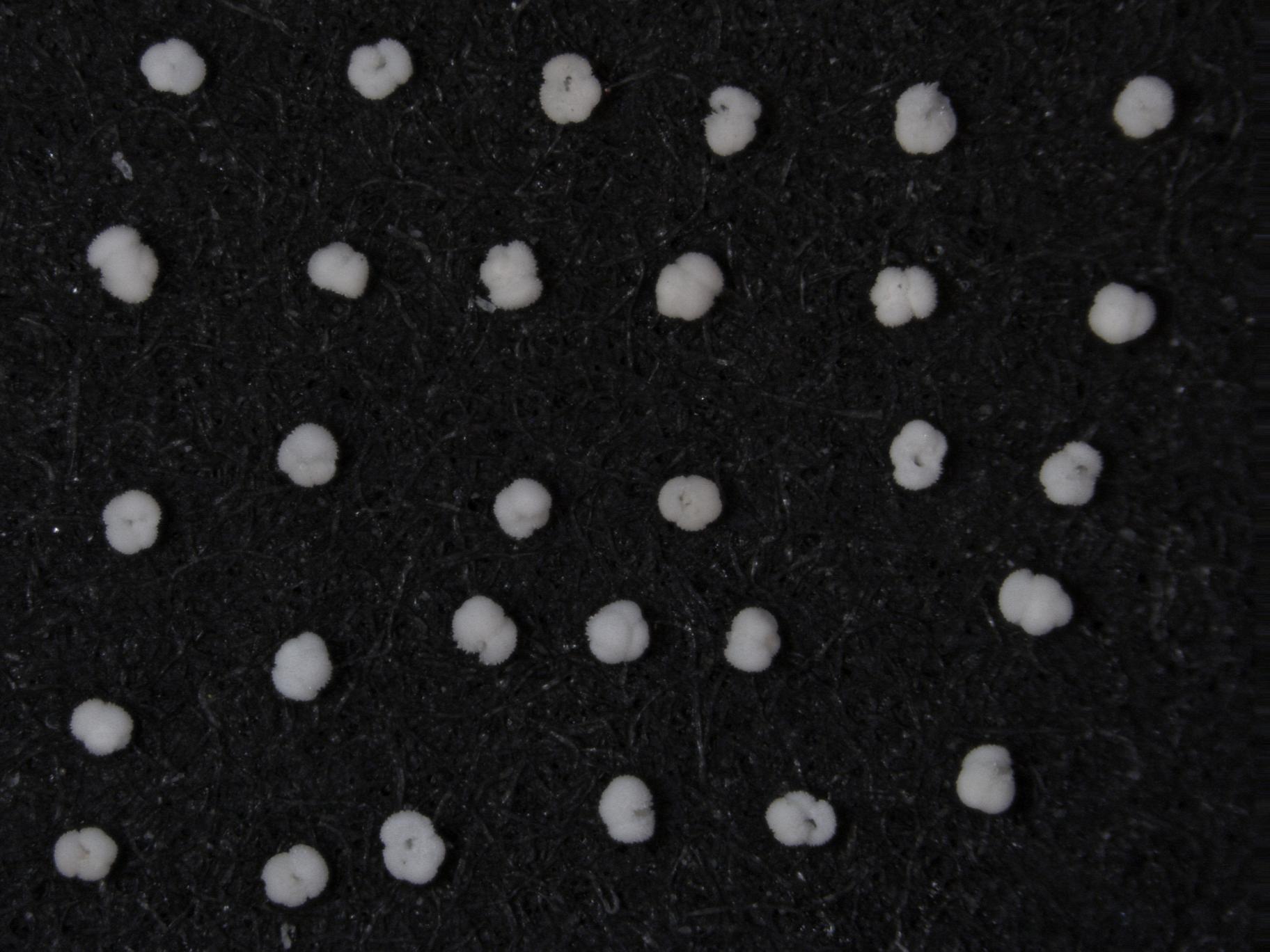

Supplement: S1 Data — (ZIP) [file pone.0267636.s001.zip › SDataImages/1209A-21H-3W_77-79_180_Aca1_2.5x_STACKED.jpg]

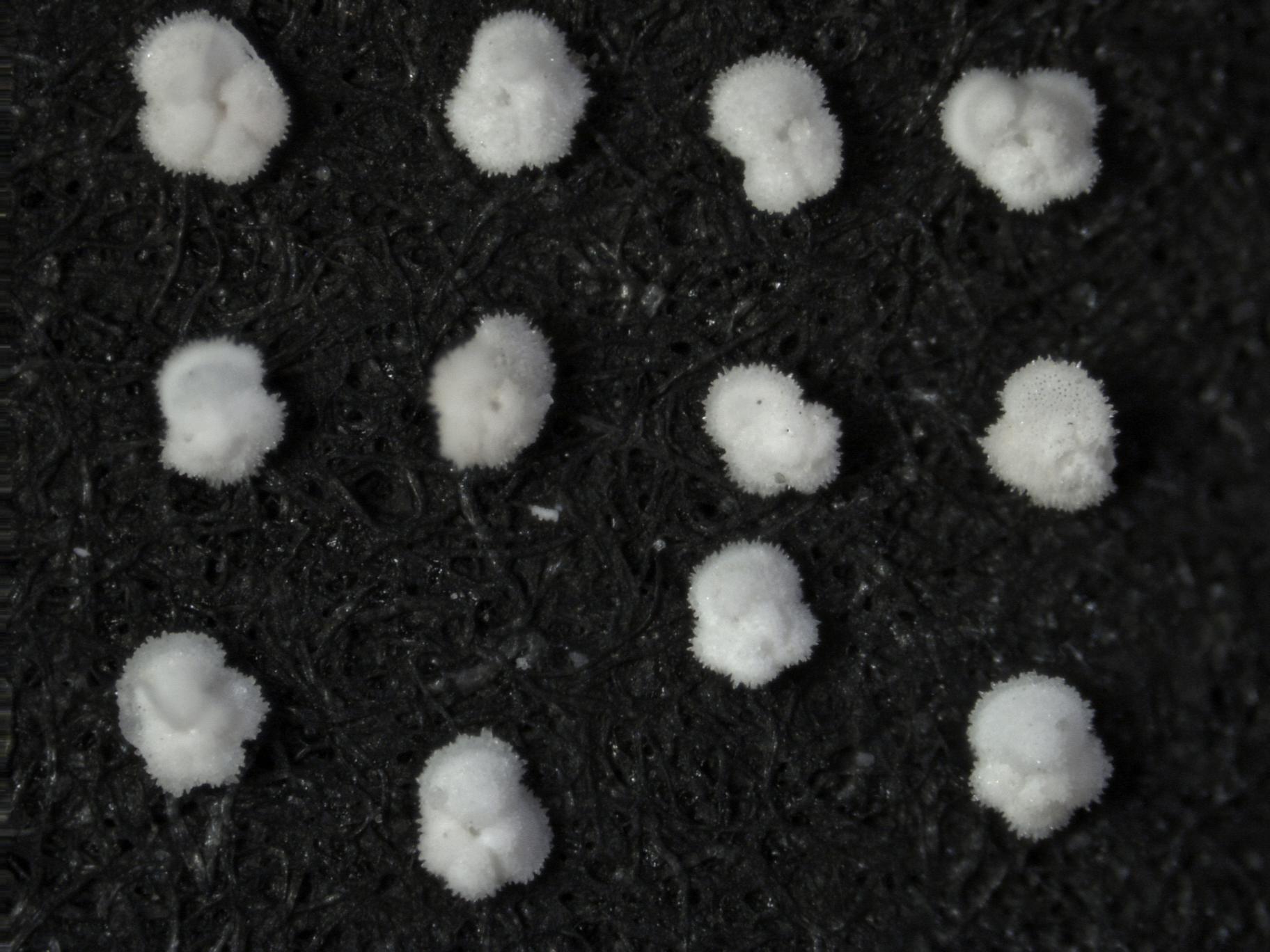

Supplement: S1 Data — (ZIP) [file pone.0267636.s001.zip › SDataImages/1209A-21H-3W_38-40_180_Mor2_5.0x_STACKED.jpg]

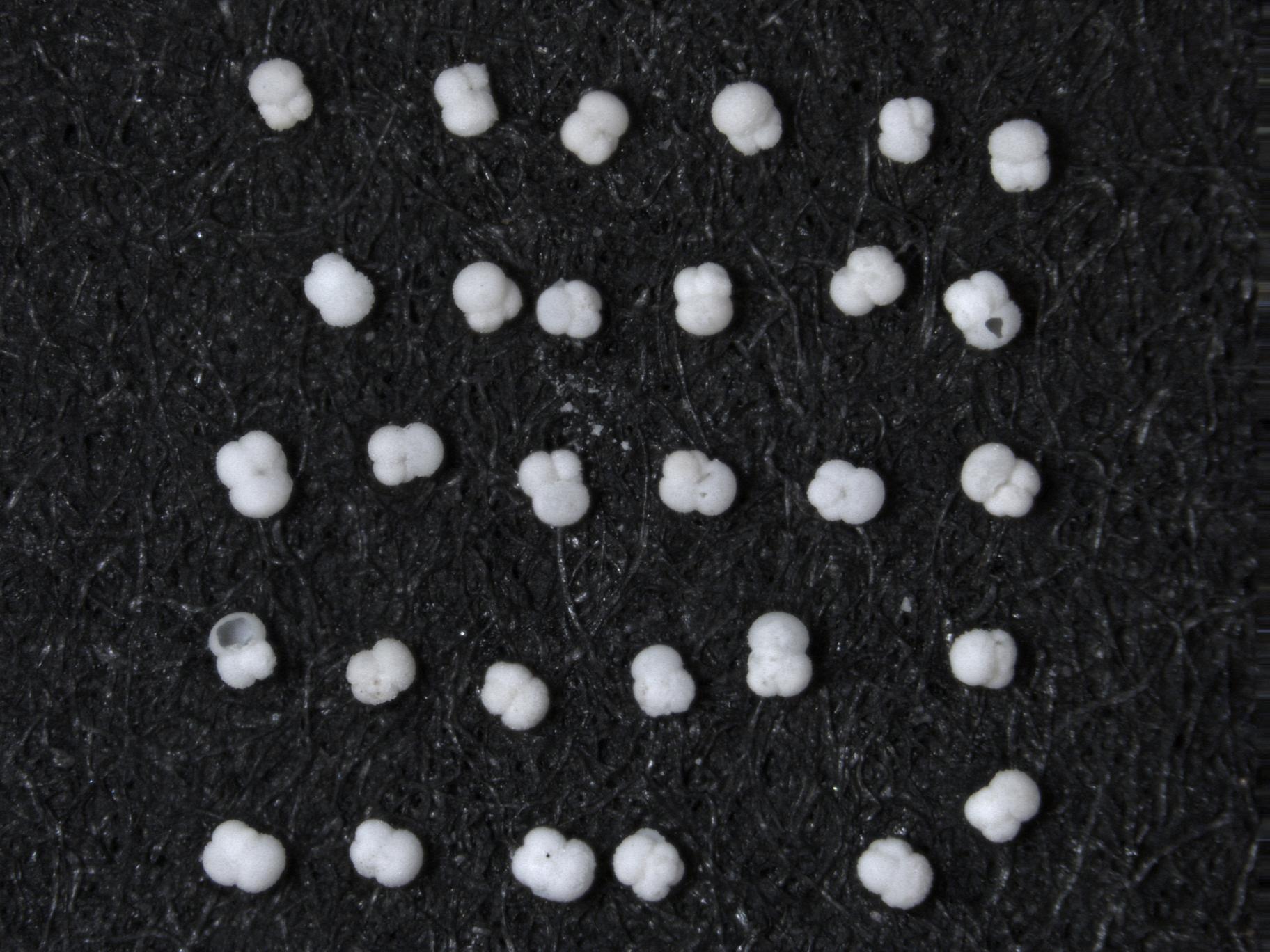

Supplement: S1 Data — (ZIP) [file pone.0267636.s001.zip › SDataImages/1209A-21H-3W_87-89_150_Sub1_3.2x_STACKED.jpg]

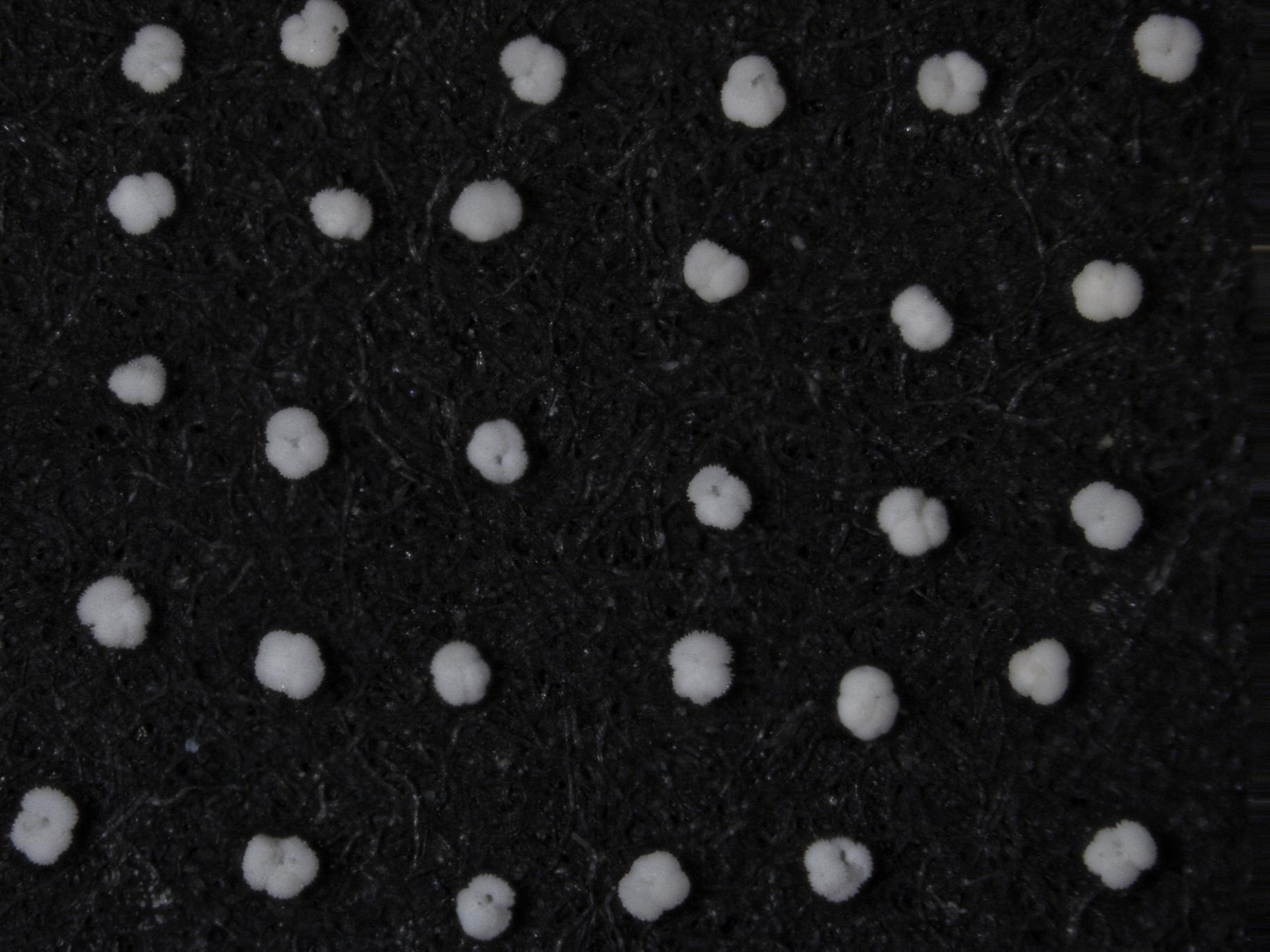

Supplement: S1 Data — (ZIP) [file pone.0267636.s001.zip › SDataImages/1209A-21H-3W_7-9_150_Aca1_3.2x_STACKED.jpg]

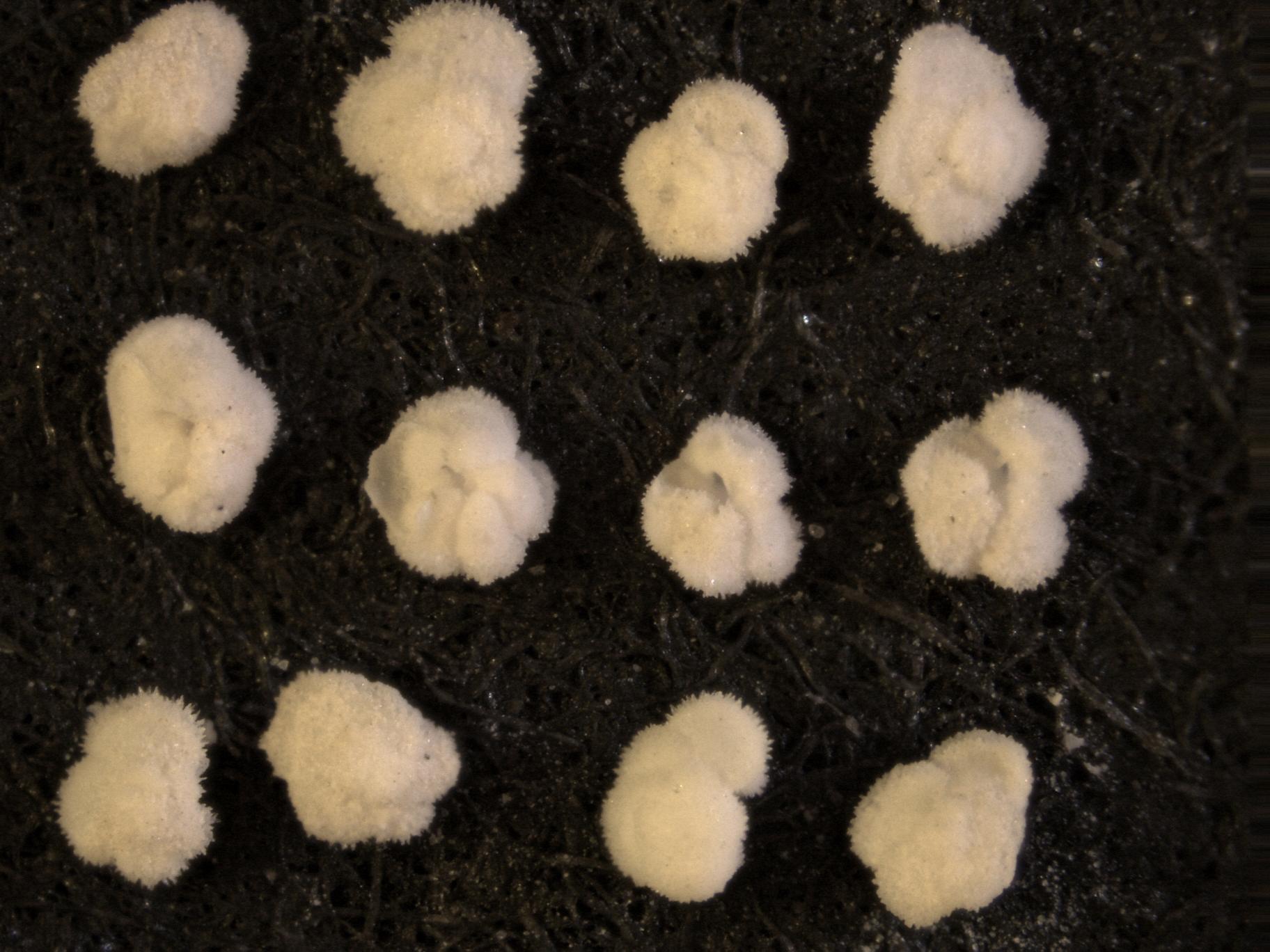

Supplement: S1 Data — (ZIP) [file pone.0267636.s001.zip › SDataImages/1209A-21H-3W_58-60_300_Mor2_4.0x_STACKED.jpg]

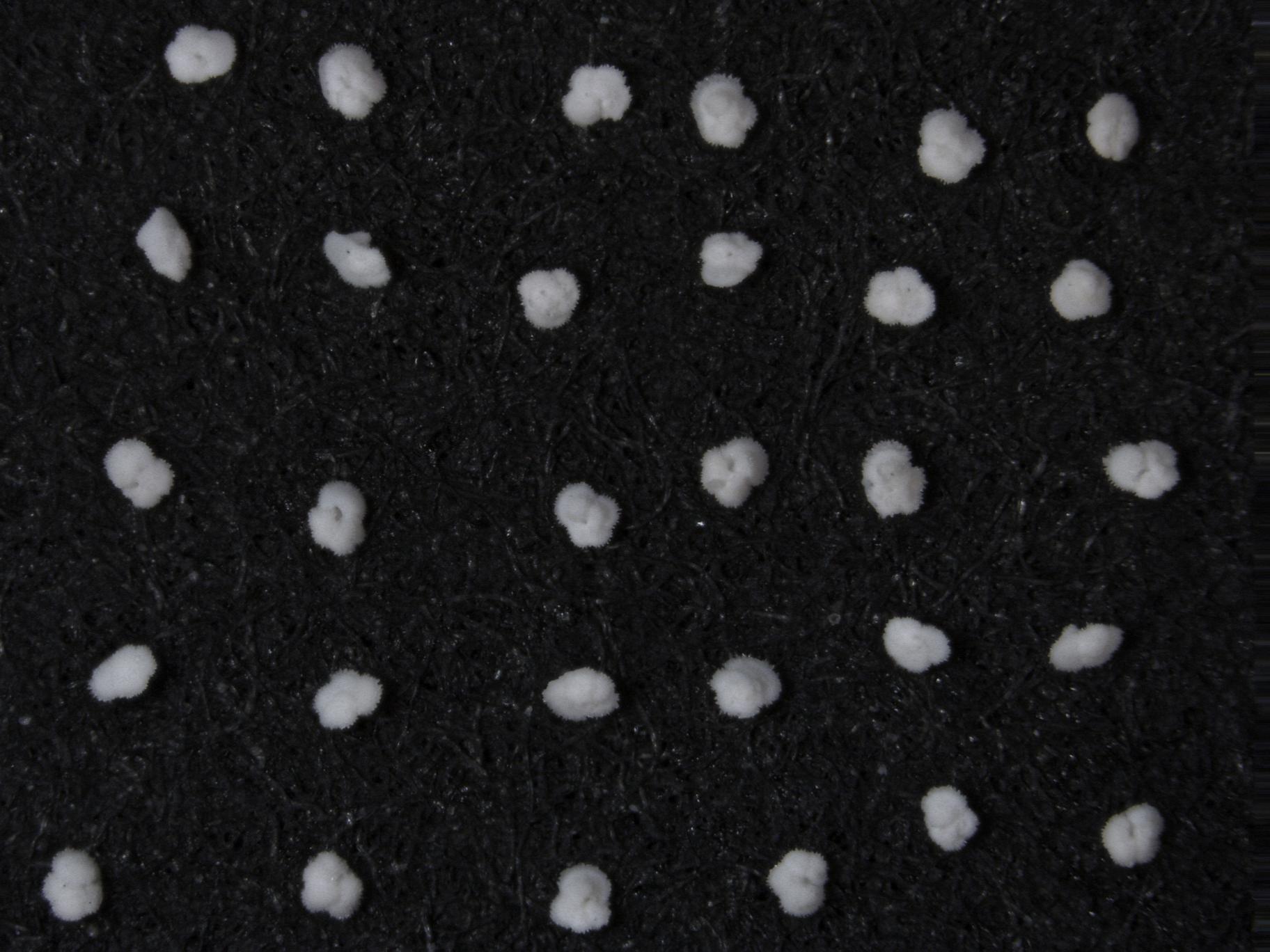

Supplement: S1 Data — (ZIP) [file pone.0267636.s001.zip › SDataImages/1209A-21H-3W_47-49_150_Mor1_2.5x_STACKED.jpg]

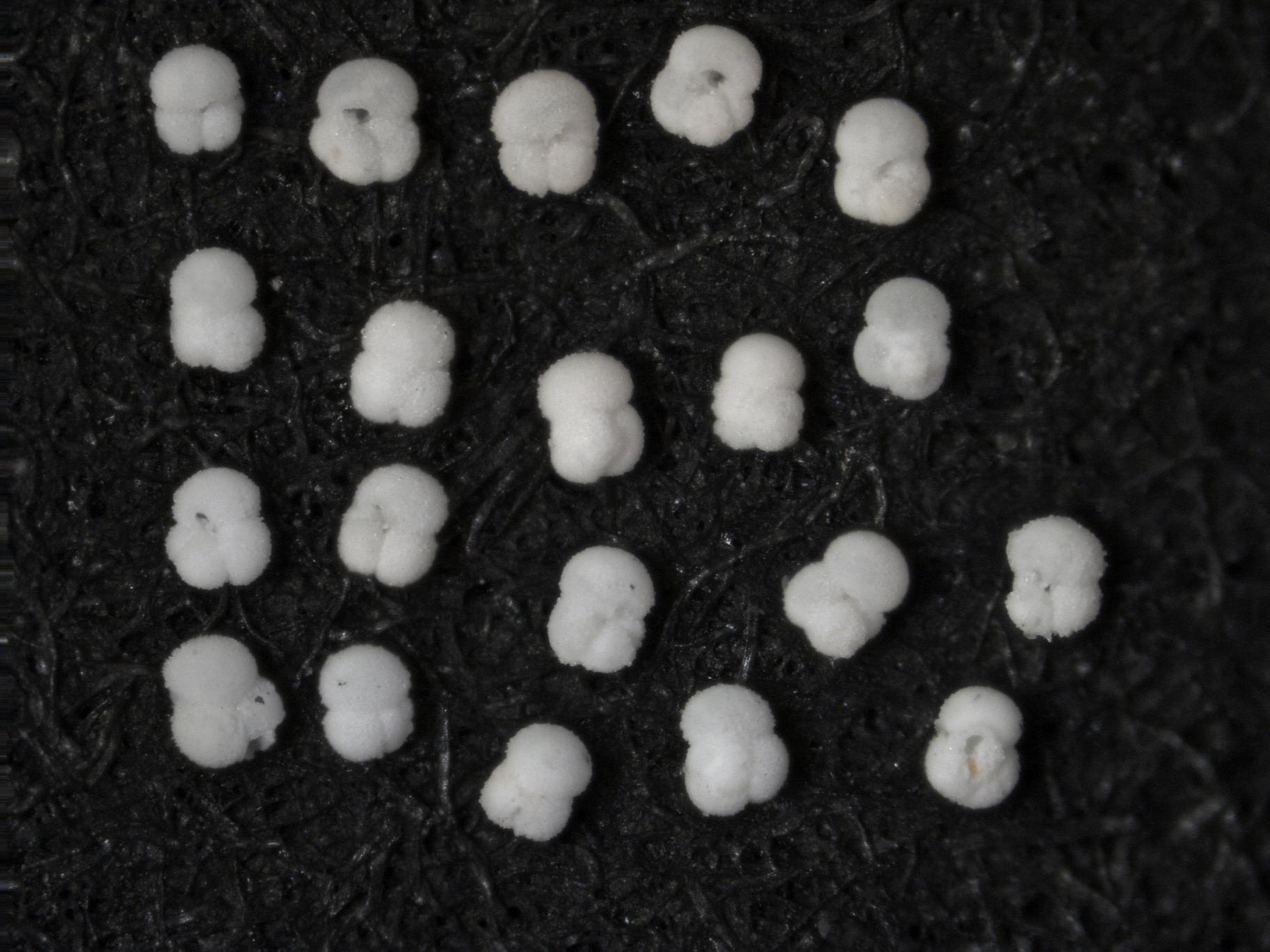

Supplement: S1 Data — (ZIP) [file pone.0267636.s001.zip › SDataImages/1209A-21H-3W_38-40_150_Sub1_5.0x_STACKED.jpg]

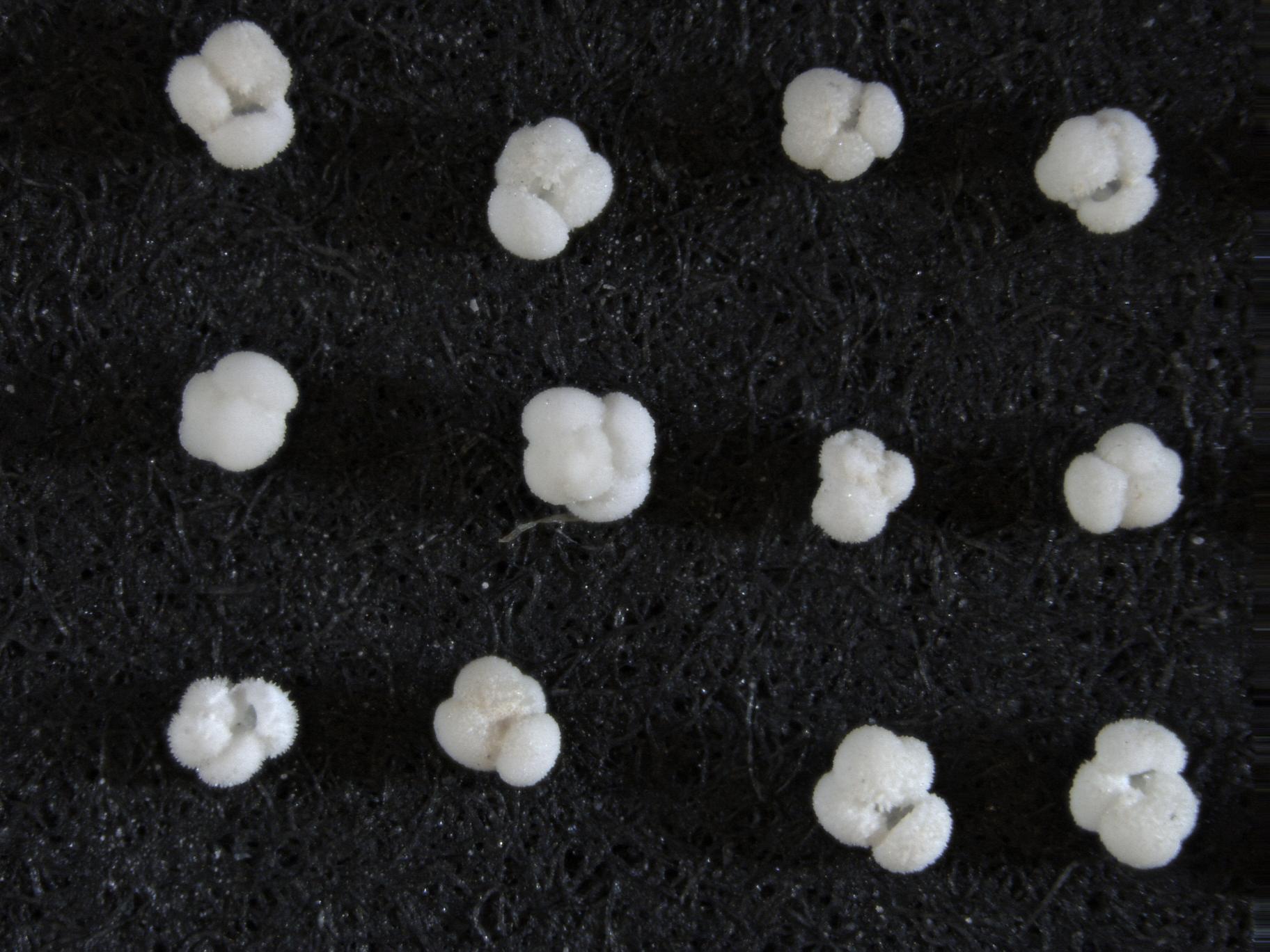

Supplement: S1 Data — (ZIP) [file pone.0267636.s001.zip › SDataImages/1209A-21H-3W_58-60_355_Aca2_2.5x_STACKED.jpg]

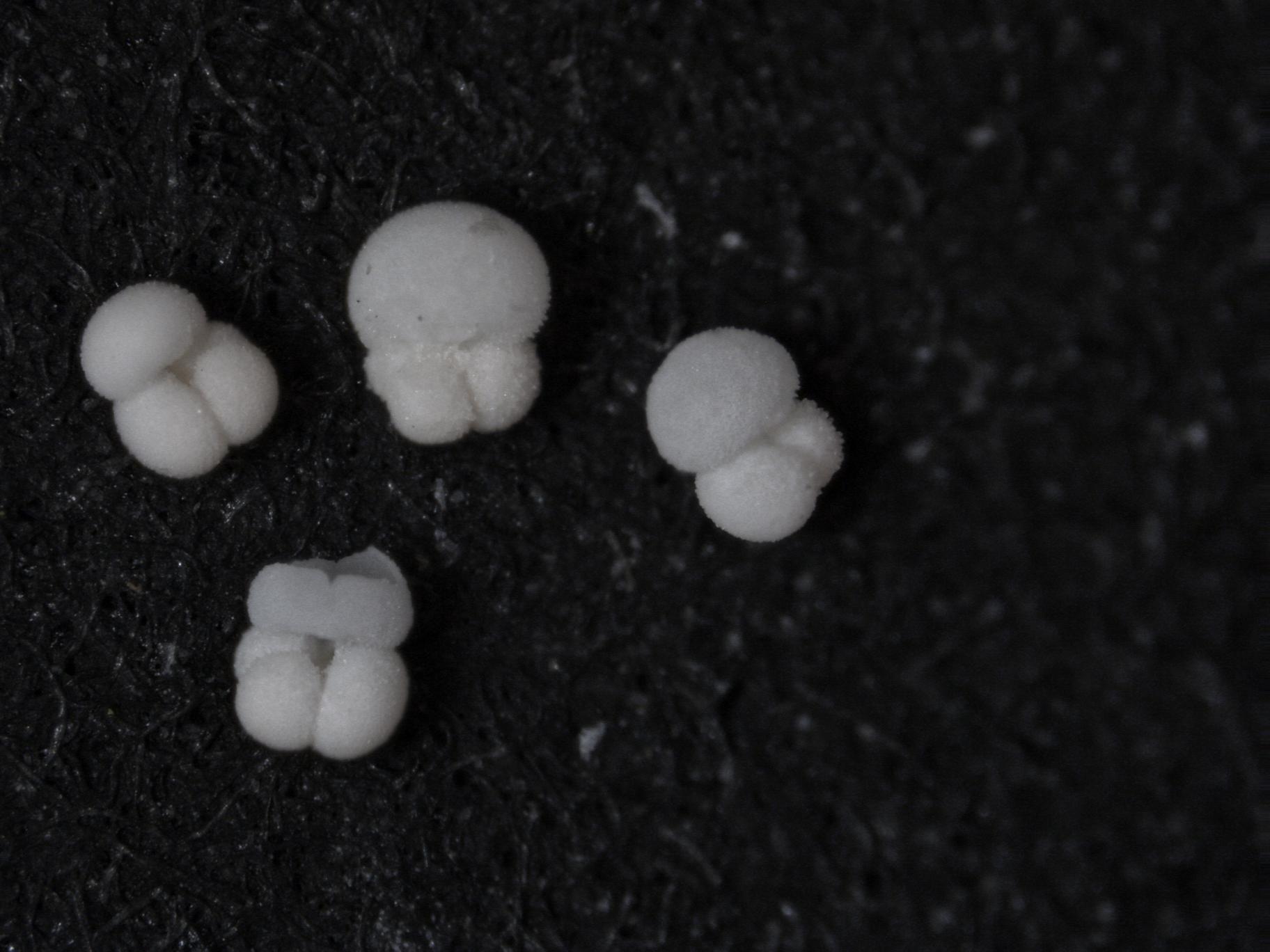

Supplement: S1 Data — (ZIP) [file pone.0267636.s001.zip › SDataImages/1209A-21H-3W_68-70_355_Sub1_4.0x_STACKED.jpg]

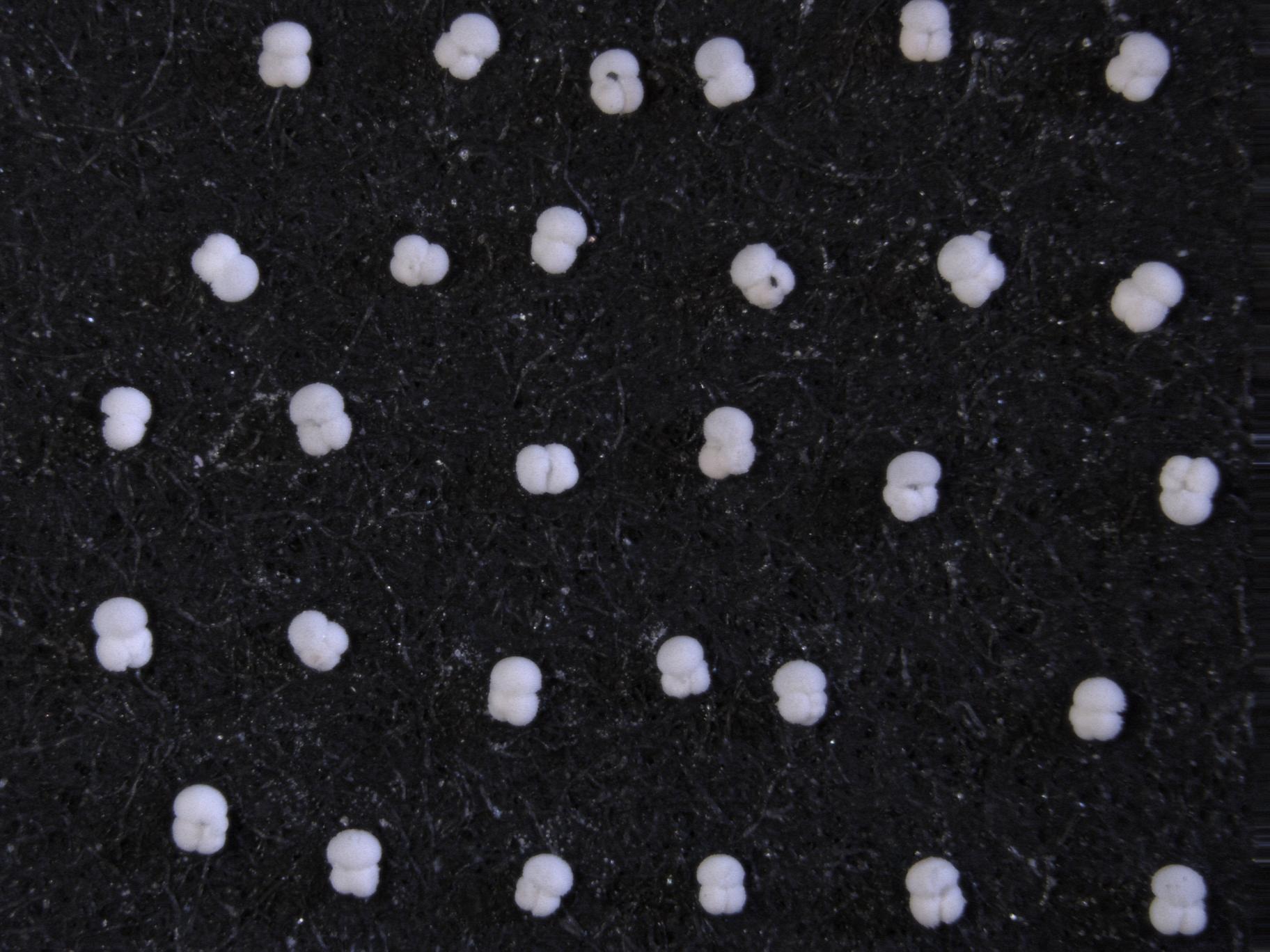

Supplement: S1 Data — (ZIP) [file pone.0267636.s001.zip › SDataImages/1209A-21H-2W_86-88_212_Sub1_2.0x_STACKED.jpg]

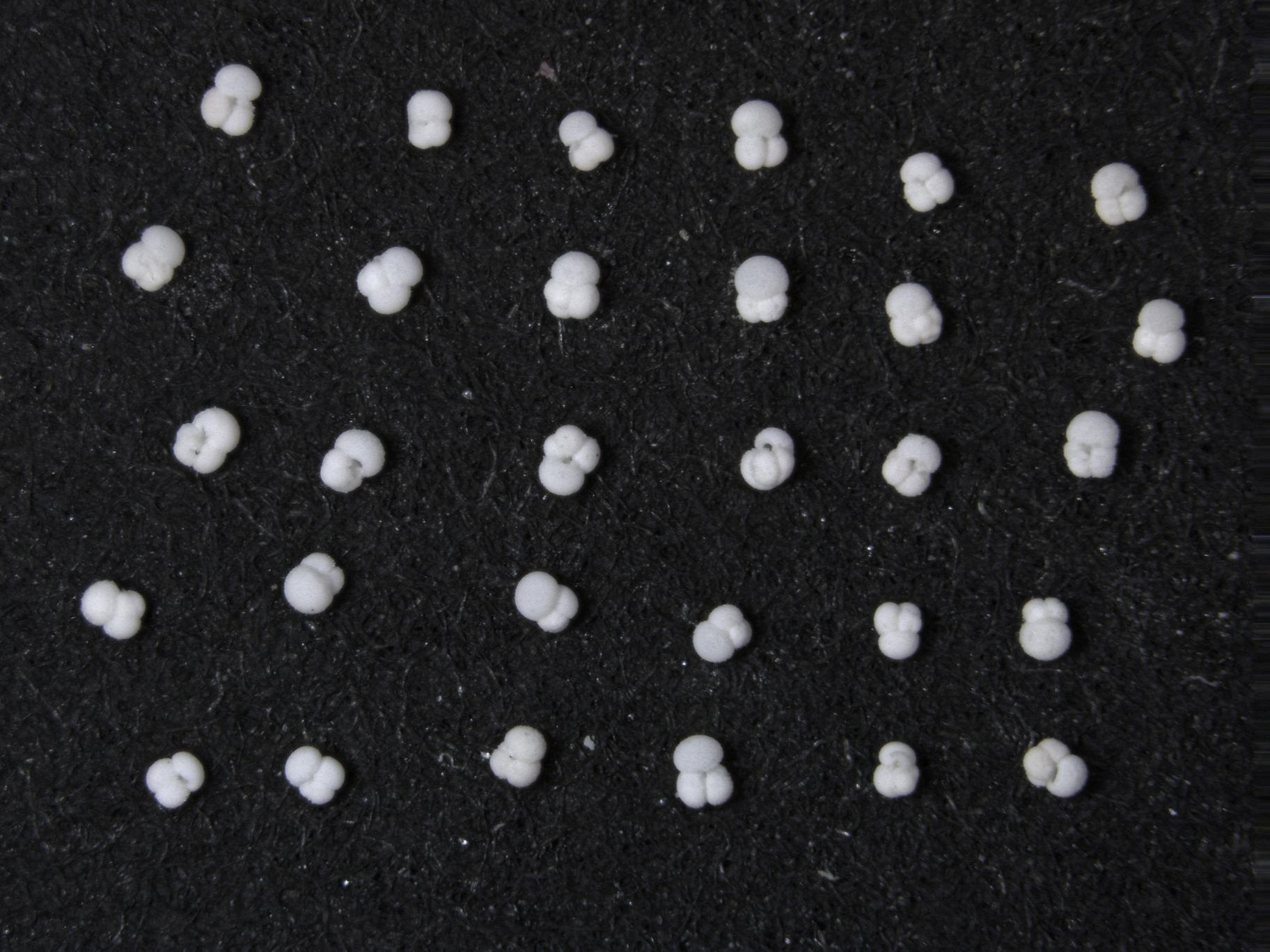

Supplement: S1 Data — (ZIP) [file pone.0267636.s001.zip › SDataImages/1209A-21H-3W_47-49_250_Sub1_1.6x_STACKED.jpg]

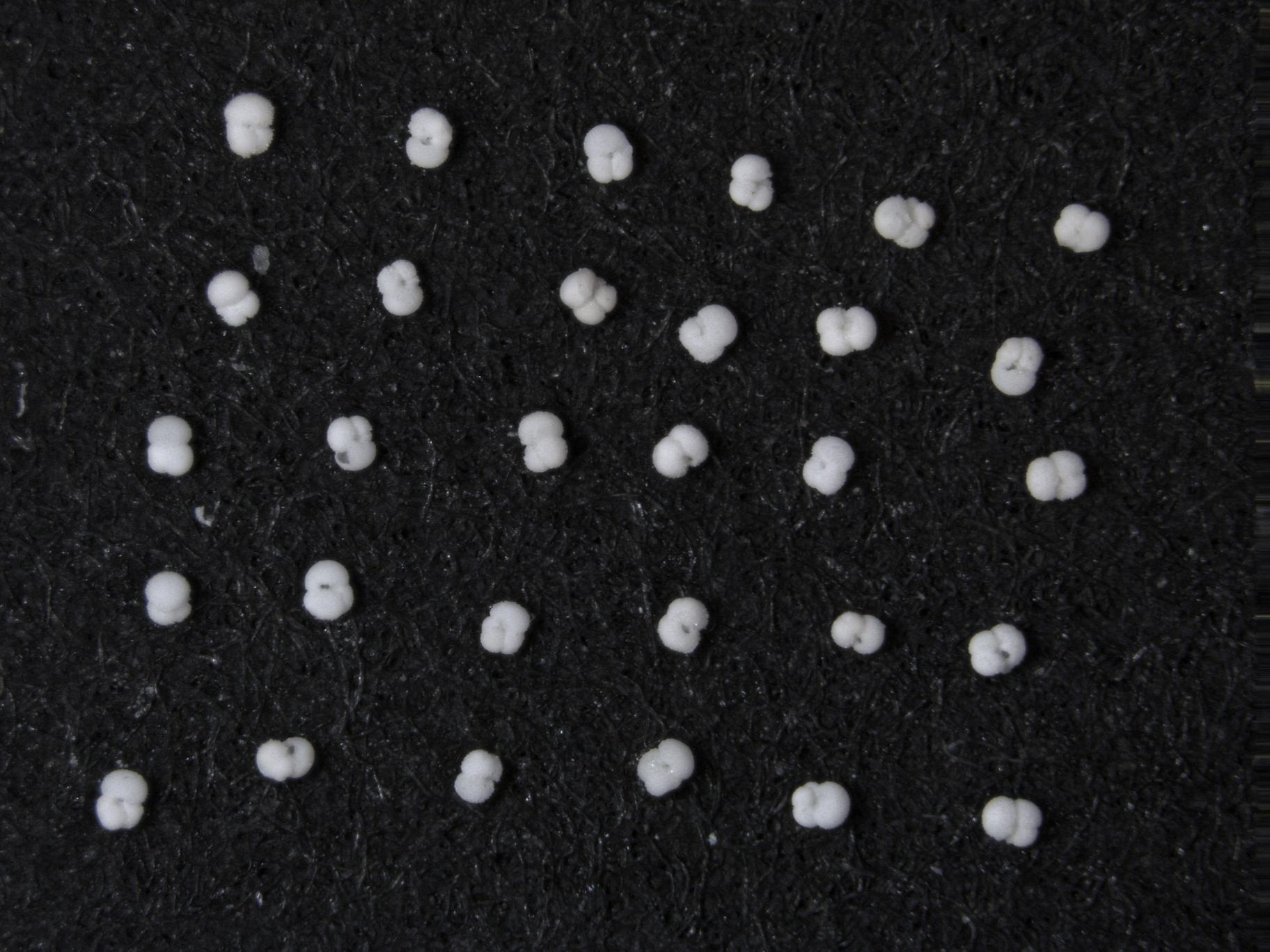

Supplement: S1 Data — (ZIP) [file pone.0267636.s001.zip › SDataImages/1209A-21H-3W_47-49_180_Sub1_2.0x_STACKED.jpg]

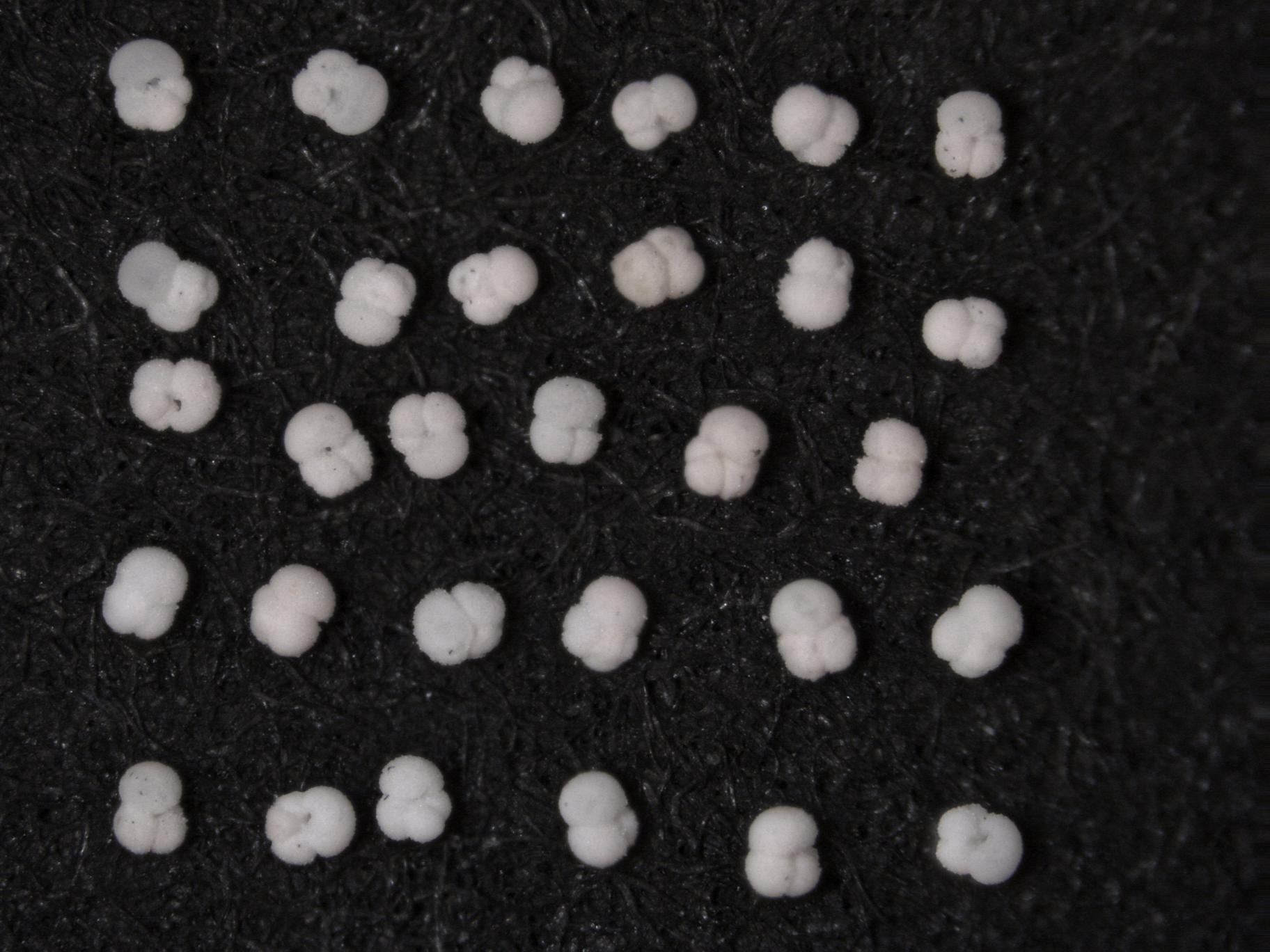

Supplement: S1 Data — (ZIP) [file pone.0267636.s001.zip › SDataImages/1209A-21H-3W_18-20_180_Sub1_3.2x_STACKED.jpg]

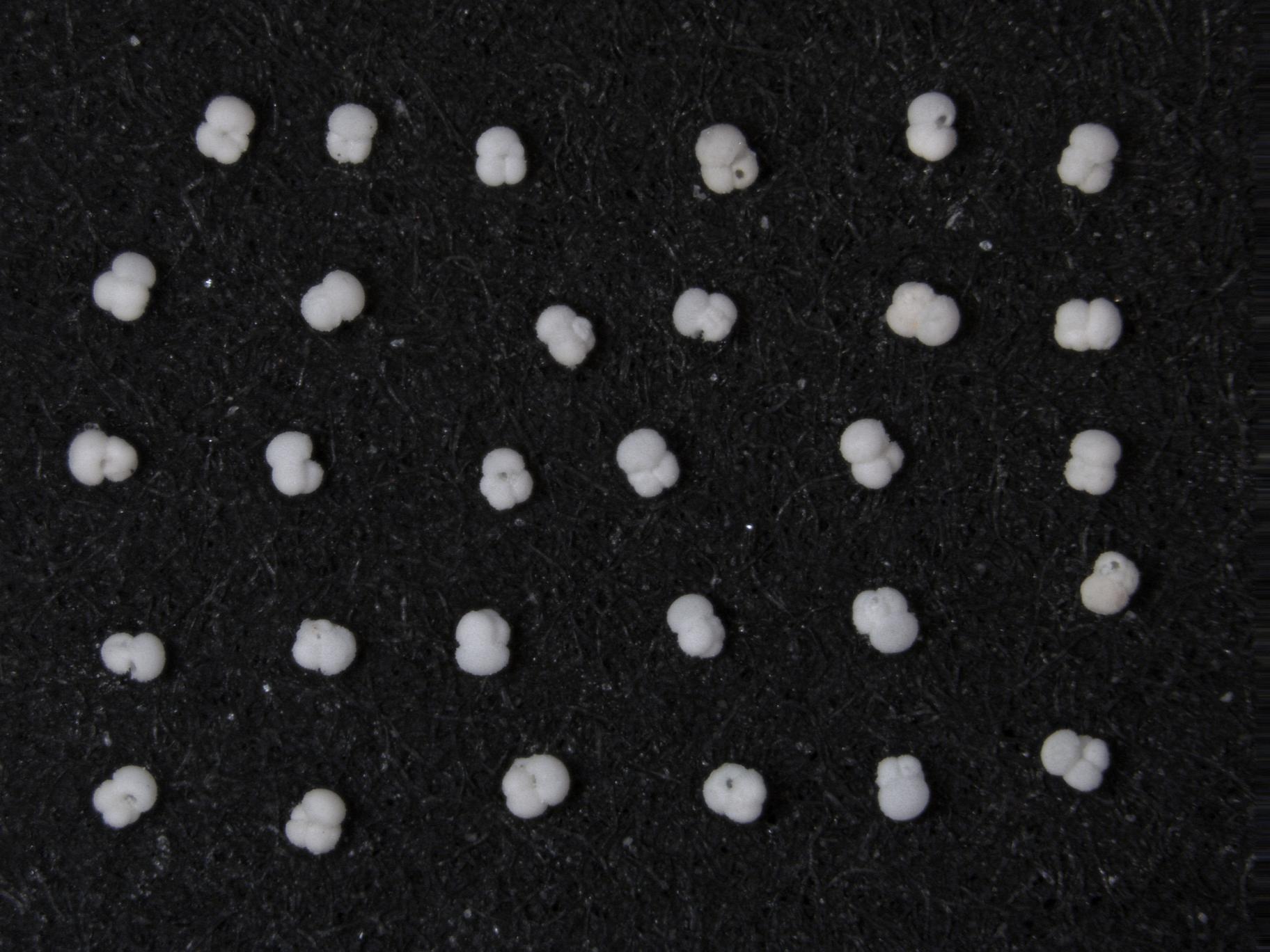

Supplement: S1 Data — (ZIP) [file pone.0267636.s001.zip › SDataImages/1209A-21H-3W_7-9_212_Sub1_2.0x_STACKED.jpg]

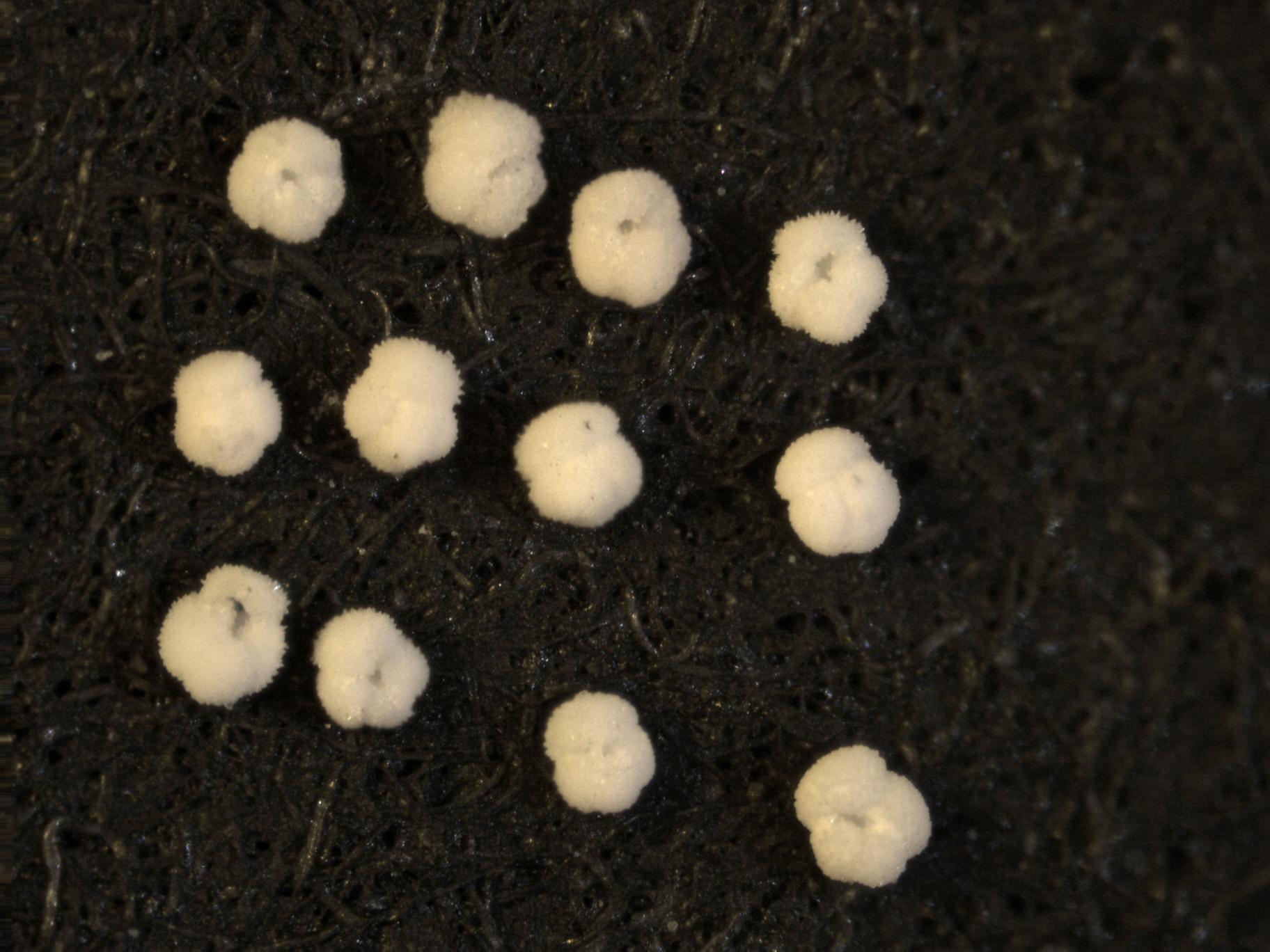

Supplement: S1 Data — (ZIP) [file pone.0267636.s001.zip › SDataImages/1209A-21H-3W_58-60_180_Aca1_5.0x_STACKED.jpg]

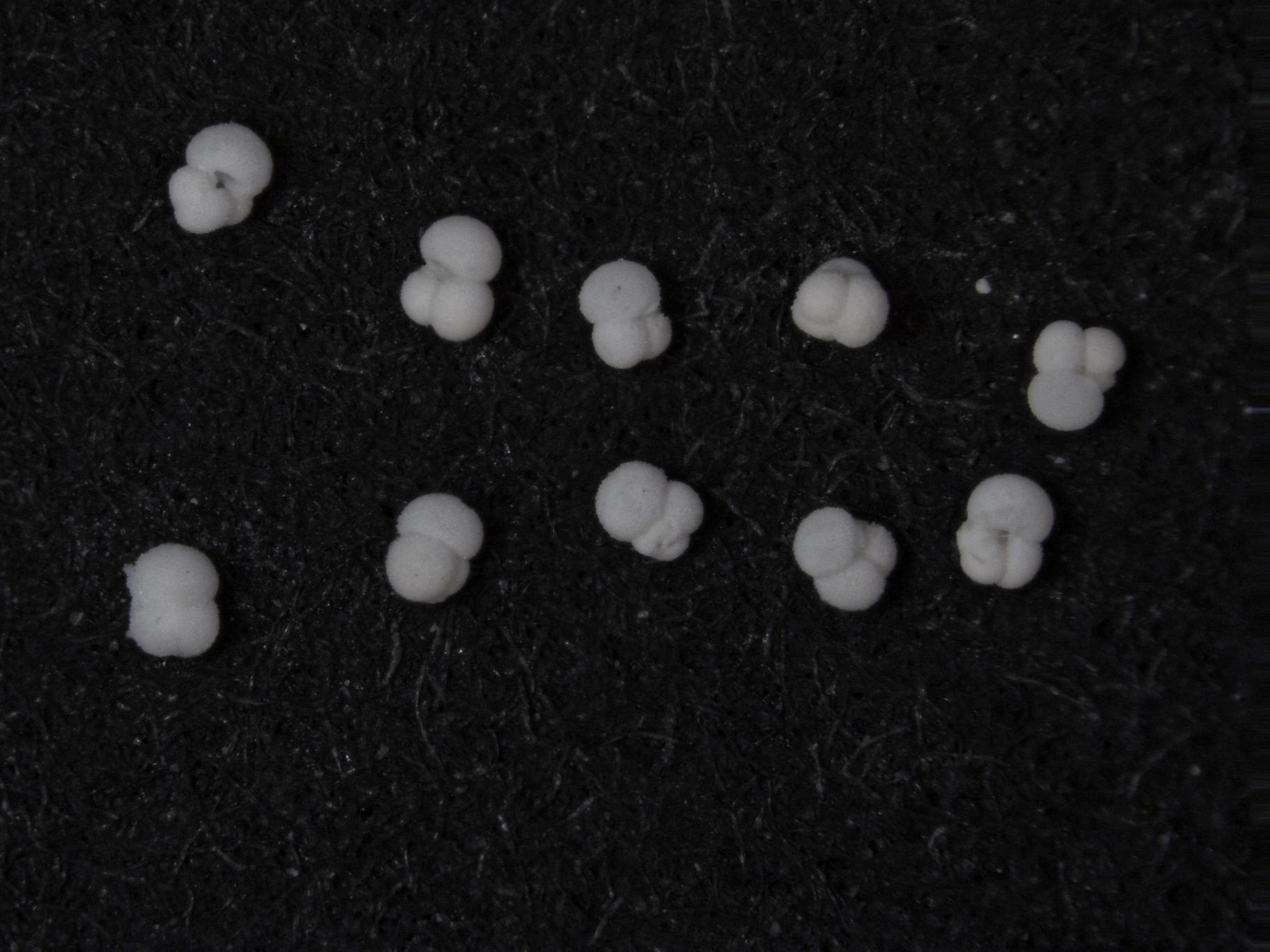

Supplement: S1 Data — (ZIP) [file pone.0267636.s001.zip › SDataImages/1209A-21H-3W_7-9_300_Sub1_2.5x_STACKED.jpg]
