# Supplementary material for: Photosymbiont associations persisted in planktic foraminifera during early Eocene hyperthermals at Shatsky Rise (Pacific Ocean)
Source: PLoS One. 2022 Sep 26;17(9):e0267636. doi: 10.1371/journal.pone.0267636 (PMC9512218; doi:10.1371/journal.pone.0267636)
Supplement: S1 Fig — Shown are the smallest (A-C) and largest (D-F) size fraction analyzed at 53.816 Ma, and the smallest (G-I) and largest (J-L) size fraction analyzed at 56.632 Ma. All sample images have been included as a supplementary dataset. (PDF) [file pone.0267636.s004.pdf]

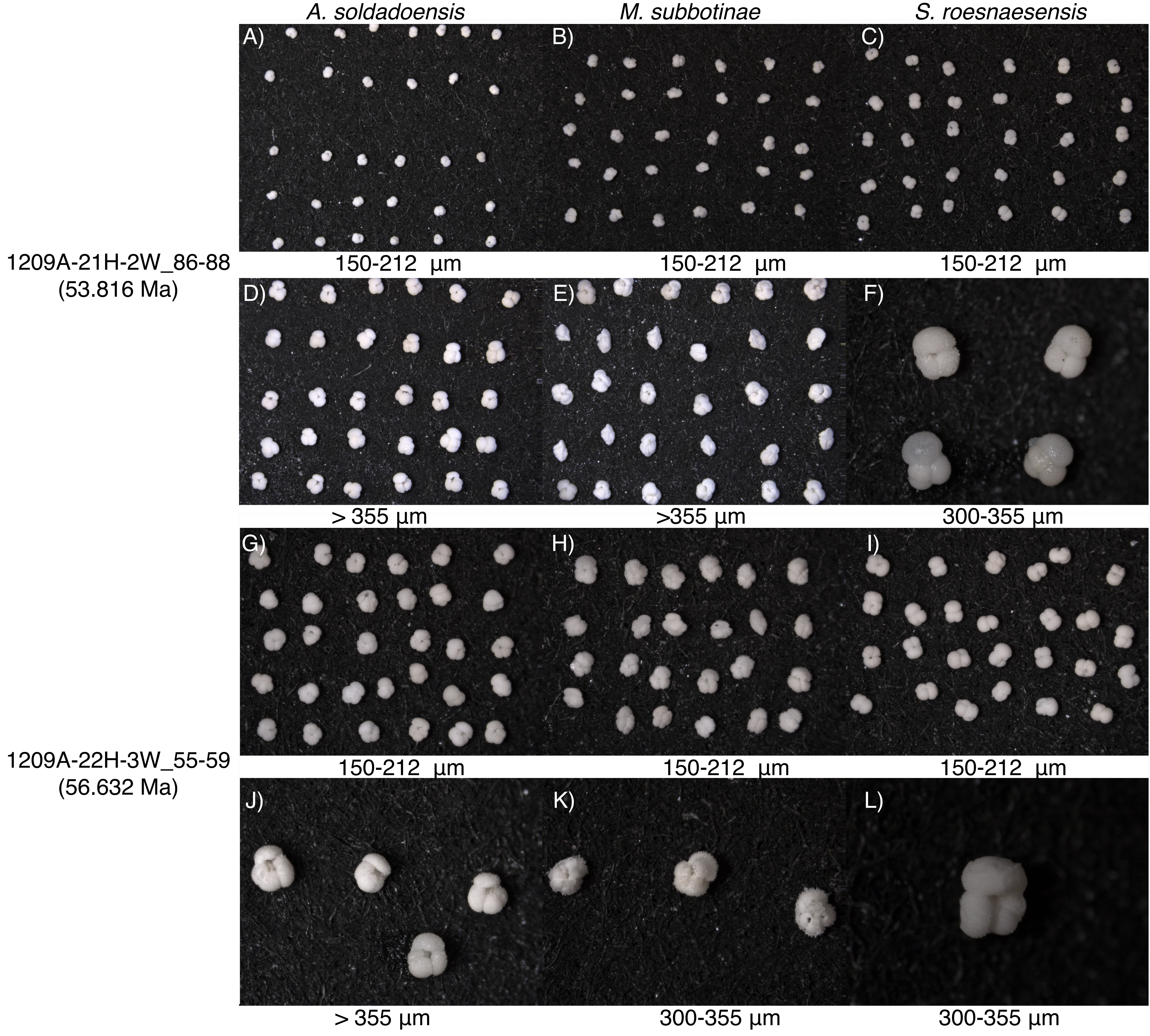
A. soldadoensis
M. subbotinae
S. roesnaesensis
A)
B)
C)
1209A-21H-2W_86-88
(53.816 Ma)
150-212 µm
150-212 µm
150-212 µm
D)
E)
F)
> 355 µm
>355 µm
300-355 µm
G)
H)
I)
1209A-22H-3W_55-59
(56.632 Ma)
150-212 µm
150-212 µm
150-212 µm
J)
K)
L)
> 355 µm
300-355 µm
300-355 µm
