# Supplementary figures and images for: Photosymbiont associations persisted in planktic foraminifera during early Eocene hyperthermals at Shatsky Rise (Pacific Ocean) (part 3 of 3)
Source: PLoS One. 2022 Sep 26;17(9):e0267636. doi: 10.1371/journal.pone.0267636 (PMC9512218; doi:10.1371/journal.pone.0267636)

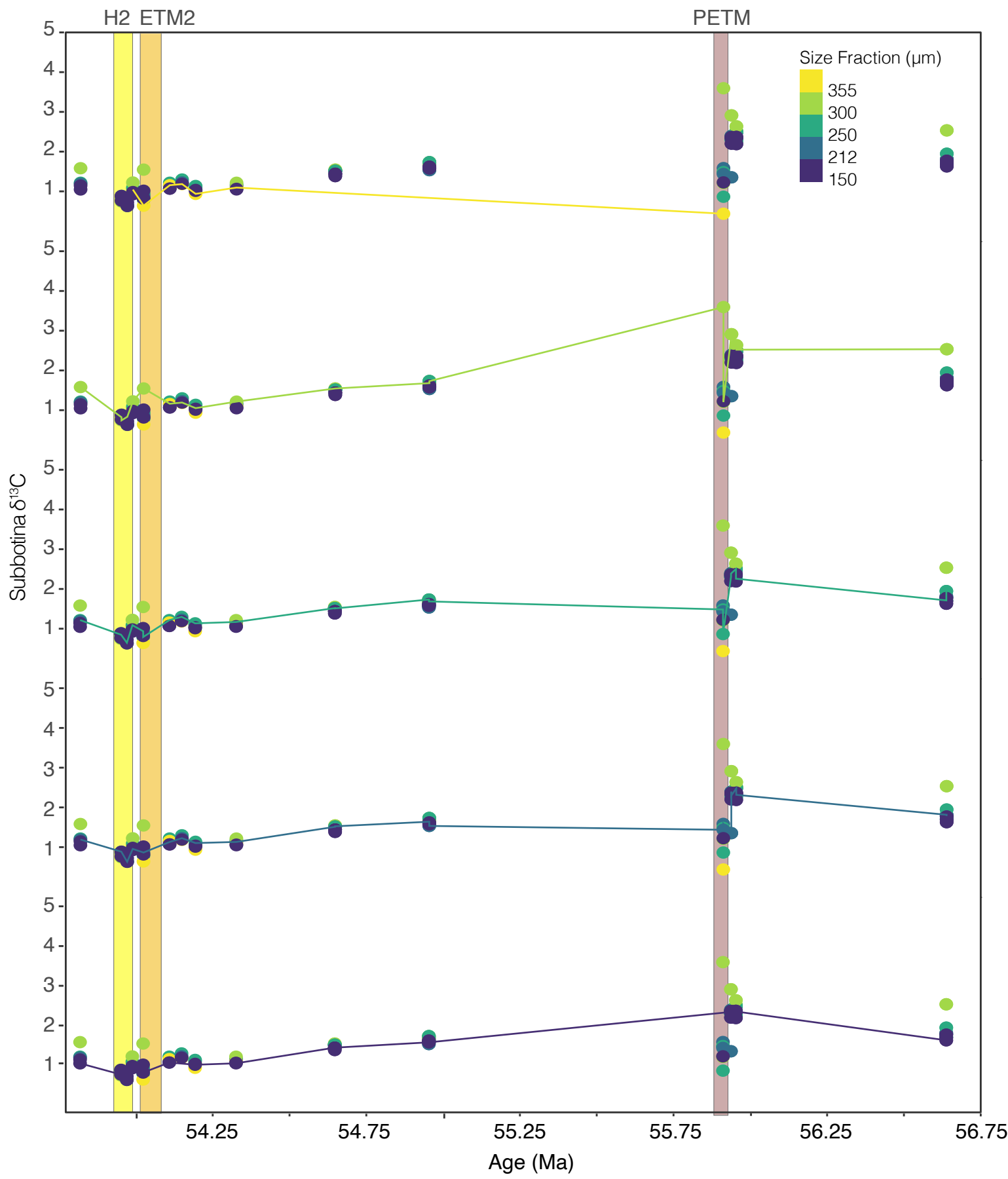

Supplement: S2 Fig — The H2, ETM2, and PETM intervals are designated with yellow, orange, and brown shading respectively. All size points are shown in each panel, with differing trends across size fractions shown in colored lines. (PDF) [file pone.0267636.s005.pdf]

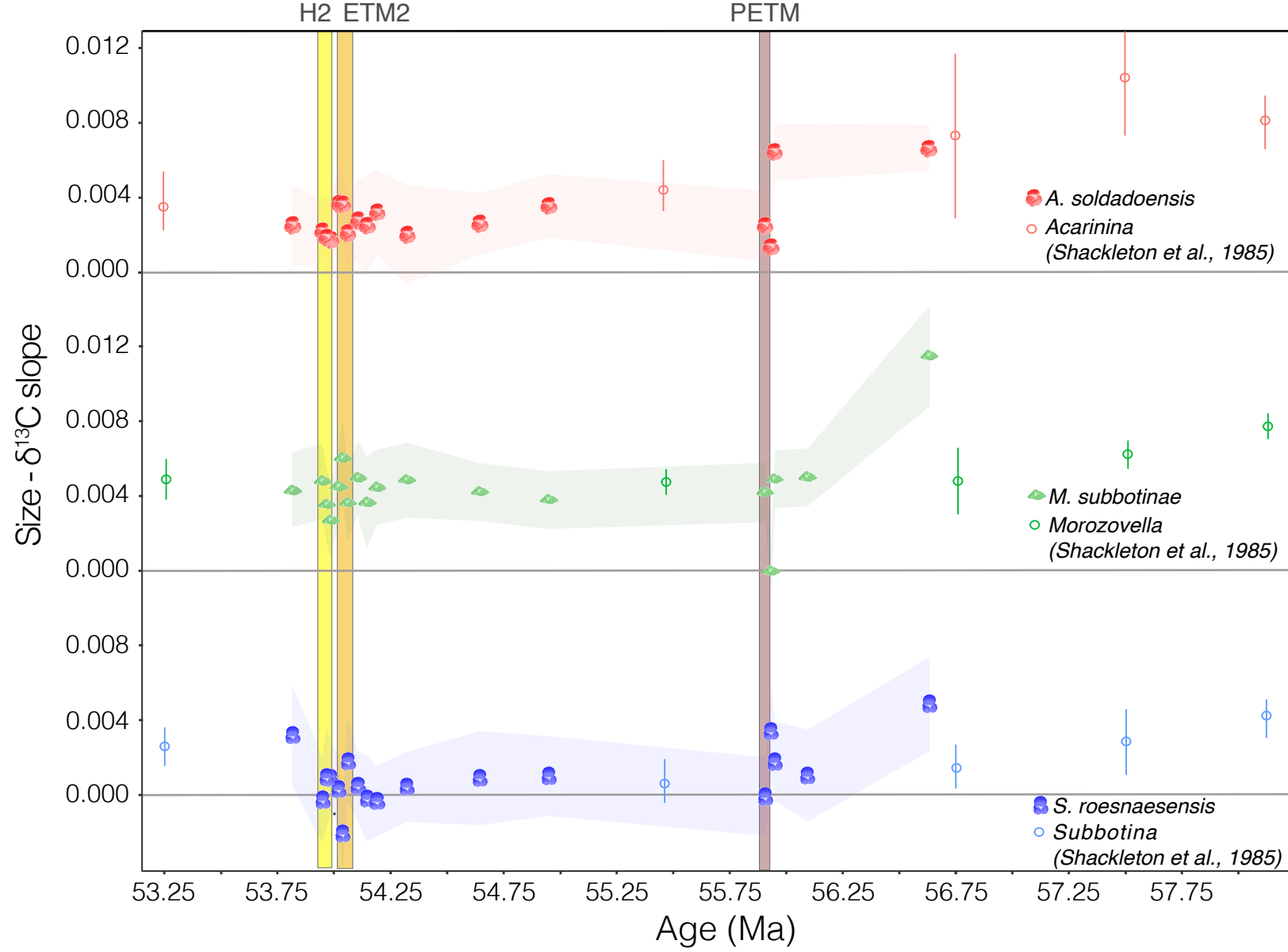

Supplement: S3 Fig — The slope of δ13C relative to size for A. soldadoensis (red), M. subbotinae (green), and S. roesnaesensis (blue) through time at Site 1209 shown as cartoon foraminifera compared with slopes from multiple species at the genus level for Acarinina (red), Morozovella (green) and Subbotina (blue) from ODP Site 577 [19] rescaled to the age model of [41] shown as open circles with error bars. The H2, ETM2, and PETM intervals are designated with yellow, orange, and brown shading respectively. (PDF) [file pone.0267636.s006.pdf]

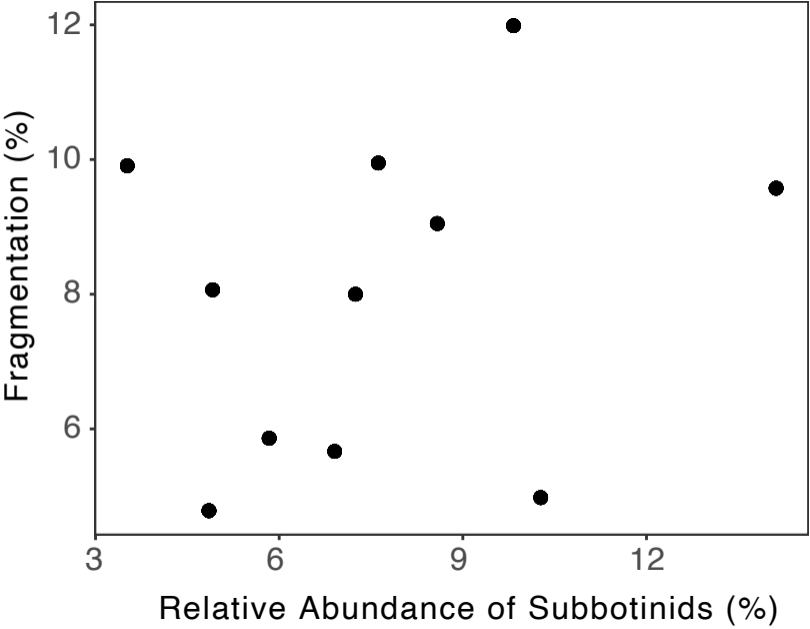

Supplement: S4 Fig — (PDF) [file pone.0267636.s007.pdf]
