# Supplementary material for: Photosymbiont associations persisted in planktic foraminifera during early Eocene hyperthermals at Shatsky Rise (Pacific Ocean)
Source: PLoS One. 2022 Sep 26;17(9):e0267636. doi: 10.1371/journal.pone.0267636 (PMC9512218; doi:10.1371/journal.pone.0267636)
Supplement: Attachment — Submitted filename: ReviewerResponse.docx [file pone.0267636.s008.docx]

We’d like to thank both reviewers for their critical assessment of our manuscript. We have tried to address each of the issues raised below.

Reviewer 1

*“As can be seen from the figures 2 and 5 (or 6? Figure numbers in the main text do not correspond to the figure captions)…”*

We apologize for this mistake, and this has been corrected throughout.

*“samples are possibly not available from the PETM “peak event”, other than suggested by figures 1, 4, and 5.”*

We did not produce data for the PETM. The data for the PETM is from the publication by Shaw et al., (2021), and used as a comparison to our records from the ETM2 and H2 events. We used all data available for the PETM at this site in our comparison.

*“Why does the figure 3 not include data points from the warm time intervals?”*

Figure 3 presents a reanalysis of data from Shackleton et al. (1985), which we found interesting in that they show the same general trend as our data over an overlapping time period. The original dataset did not sample the lesser hyperthermals.

*“Assuming that reliable isotope data would be present on ETM2 and H2, reasoning on the effects on photosymbionts and the d13C of the different planktonic foraminifera shells would be based on only two time intervals.”*

Yes, we have focused on two hyperthermal events and flanking time intervals for our study, for a total of 11 time slices. But we have also included data reanalysis from a third event and flanking intervals (PETM). These are the more prominent hyperthermals.

*“However, in the lines 177 and 178 of manuscript it says that “Planktic foraminifera from ODP Site 1209 are frosty with poor to moderate preservation throughout the sampled interval”, which brings the entire reasoning into doubt. I’m actually surprised by the assessment on the quality of foraminifera shells, because good preservation is reported in the initial ODP reports. May preservation have suffered over time in the core repository?”*

Some confusion exists about what 'good preservation' means exactly, and how different authors at different times expressed it, which is due to development of the concept of preservation state of planktic foraminifera during the last few decades. We probably did not explain this clearly for a non-specialist audience. Most samples obtained by DSDP, ODP and IODP contain planktic foraminifera assessed as 'well preserved' in the past, i.e., before studies by e.g. Pearson et al, 2001; Sexton et al., 2006, who documented that there was considerable recrystallization even of these so-called 'well-preserved' foraminifera. Well preserved foraminifera as now defined ('glassy' preservation) are generally absent in carbonate oozes (such as we studied), the most common sediment recovered by ocean drilling for paleoceanographic studies. Glassy preservation occurs only in clay rich sediments, whereas carbonate oozes contain 'frosty' specimens, as the ones we studied. As widely documented, this recrystallization affects oxygen isotope signatures strongly (even in sediments as young as Miocene), but carbon isotope signatures (which we are investigating here) are very little affected, because there is so much less dissolved carbon in pore waters than oxygen in water. This is explained in detail and well documented for the PETM specimens in Shaw et al., 2021, to which we now more clearly refer; the preservation of specimens is also shown in the supplemental data to that paper available at https://doi.pangaea.de/10.1594/PANGAEA.918702. For this manuscript, photographs of specimens showing preservation are shown in supplemental Figure 1 and the Supplemental data. We have also revised our discussion of preservation on lines 170-180 to reflect some of this nuance.

Our assessment follows Petrizzo et al., (2008) in their evaluation of the PETM, as well as the documentation in Shaw et al. (2021). Thus, our statement of ‘poor to moderate’ is a conservative estimate of preservation, as it refers to likely the interval of worst preservation in any considered interval. We have revised to ‘moderate’ to better reflect the description of sediments well above the PETM. Unfortunately, there are not sufficient constraints to determine whether preservation has changed during core storage based on our sample set alone. In general, changes in preservation of planktic foraminifera have not been observed in organic-poor carbonate oozes such as studied here, even those stored for decades, i.e., obtained during the earlier DSDP expeditions in the early 1970s, under the cooled conditions in the core repositories.

*“The Paleocene and Eocene hyperthermals have been shown to be possibly caused by high atmospheric (and by diffusion marine) methane concentration (e.g., Zachos et al. 2005) and high CO2, both of which molecules would have a strong effect on the d13C signal of the foraminifera species discussed here, plus the effect of decreasing pH on foraminifera shell calcification and preservation. Consequently, the d13C signal may not reflect changing photosymbiont activity, but varying C reservoirs in ambient seawater. These effects may be resolved by assessing mass balance of the different reservoirs. However, taken the differential ecological demands and preservation of the shallow and deep dwelling species discussed here, and the poor temporal resolution of the record, this exercise may be difficult.”*

We certainly do not discount nor disregard a change in carbon reservoirs over the time period studied, since this change in reservoir values of δ^13^C is indeed a well studied topic. We used the available information to choose our time-slices to explicitly target the δ^13^C excursions associated with changing global CO_2_ and carbon cycling to better understand the ecological response of multiple species of foraminifera to those perturbations. We have tried to make this more explicit in the manuscript, to address a broader audience. Changing carbonate chemistry and source would indeed be expected to the drive δ^13^C of a bulk foraminiferal sample (or a sample within any given size bin) but does not impact the **relative difference** in δ^13^C with size within an individual species in an individual time slice. After all, each species within a time slice lives within the same macro-carbon environment. Environmental δ^13^C values and/or pH thus change between time slices, but different size classes of the same species living at the same time are living consistently in in a δ^13^C environment typical for its time. The parameter which we use, i.e., the size-δ^13^C relation, has been widely interpreted as an indication of photosymbiont activity, after ensuring that the studied specimens indeed were living at the same time at the same place, thus seeing the same background δ^13^C value in dissolved inorganic carbon and the same pH (Spero & Lea, 1993; Berger et al., 1981; Shackleton et al., 1985; D'Hondt et al., 1994; Norris, 1996; Birch et al., 2012; Edgar et al., 2013; Shaw et al., 2021; Si and Aubry, 2018; Wade et al., 2008). The goal of this paper is to better understand ecological response at the species-level to large scale perturbations, rather than characterize the Eocene carbon environment at high resolution. We have attempted to further clarify this on lines 75-77 and 176-180.

*“Statistical analyses should prove significance of any changes in the d13C”*

Statistical analyses (e.g., ANCOVA) were carried out, and are described in the document and supplemental materials. In most cases these were not found to be significant, which is one of the major findings of this manuscript. If the reviewer could clarify if and where further tests are needed, we’d be happy to revise accordingly.

*“There are many minor shortcomings in the reasoning and understanding of planktonic foraminifera biology and ecology, such as the (mis-) interpretation of “gigantism” in foraminifera; the northern hemisphere glaciation may be considered time interval of global cooling.”*

If the reviewer would specify where misunderstandings are found, we would be glad to address them in a future revision. With reference to “gigantism,” this was discussed in summarizing the findings of another paper that used this exact terminology. However, we have removed this reference entirely as the discussion likely strays too far from our main story.

*“The red symbols in the benthic stack in the figure 2 do not represent an age model worth to talk about, but rather match -at the best- the age model of Westerhold in some places.”*

We do not present a new age model for this site but use the age model of Westerhold et al. (2018) throughout. Figure 2 includes stable isotope data for multiple species of benthic foraminifera during the relevant time slices at the site, superimposed on the more complete Westerhold et al. record. This is neither a stack, nor a revision of any existing age model, but a comparison of our data with a published record. We have attempted to clarify this in Figure caption 2, since we think that this is a misunderstanding of our figure.

*“Organization of the panels in the figures 1 and S3 make comparison of data over different time intervals almost impossible the figure 1 is rather data documentation and may be moved to the supplementary materials.”*

We have revised the presentation of Figure 1 and S3, but respectfully disagree with the reviewer regarding removing Figure 1 from the main text. We believe it is important to present our size-δ^13^C data in a comparable manner to how it is has historically been shown in similar analyses, for example by Wade et al, (2008) and Edgar et al., (2013). We also think that presenting these data is important for following our argument, by a broader audience which may not be as familiar with these types of data.

*“The x-axis in the figure 3 may be significantly shortened to move the data points closer together and to see any significant (?) changes.”*

We have changed the scale on Figure 3 as suggested.

*“It’s a cute idea to present the data points in the figure S4 as little foraminifera icons, but which does not really help to identify varying shell diameter (size legend).”*

Thank you for this suggestion. This figure has been removed altogether in an effort to streamline our Supplement in response to Reviewer #2.

Reviewer 2

*“In my opinion the discussion needs to be implemented with more detailed explanation and examples and comparison with published data that are only mentioned in the text but not discussed.”*

We have reorganized some the discussion and added additional explanation

where we saw that this may be useful. If there are additional sections which require elaboration, please let us know.

*“Line 133-135- In Fig. 2 there is eocaena not roesnaesensis. Check for consistency.”*

Thank you for catching this. We apologize for this mistake, and it has been corrected throughout

*“Line 183-184. Not clear I suppose it is Fig. 2 as fig. 1 only shows the d13C record. I do not see that a partial signal is retained associated to benthic hyperthermal because soldadoensis and eocaena yield negative values also in the intervals in between the hyperthermal*.”

Our apologies for the confusion around figure numbers; we made a mistake. This has been corrected throughout and the statement has been removed entirely.

*“Line 197-202. The text of the caption refers to Fig. 4 not Fig. 3.”*

This has been corrected.

*“Line 198-199. Again, I am confused, which species of Subbotina has been analysed?”*

*S. roesnaesensis* was analysed. An earlier draft had included the incorrect species name, but this has been corrected throughout.

*“Line 213-214. I do not see any blue line for Subbotina in Fig. 5”*

This line at “0” has been added.

*“Line 224-225. There is no Fig. 6 in the copy I have.”* & *“Line 264. Fig. 6 is missing.”*

Our apologies if this was a submission error. It should be included in this version.

*“Line 274- The order of the Supplementary material needs to be revised, Supplemental Fig. 3 cannot be cited in the text before 1 and 2. There is some confusion in the order of the Supplementary material and it is not clear where the supplementary material need to be cited in the text. Please check and revise text and figures accordingly.”*

The supplementary material has been reordered and cited accordingly.

*“Line 283-284. Not clear, Fig. 5 does not show the continued preservation of whole test. I suppose there is something missing here.”*

This statement should refer to Figure 6. We have checked and corrected figure references throughout.

*“Line 257-327. I find these two chapters a little confused and repetitive. I also miss the explanation and the citations in the text of the figures included in the Supplementary material. I would advise to use those figures to support the explanation and hypothesis provided in the discussion.”*

Both sections have been edited and reorganized to improve clarity, remove repetition, and include reference to supplementary figures.

*“Another suggestion is to think about a synthesis figure or scheme that summarize the conclusions presented in the text.”*

We appreciate this suggestion but have been unable to summarize these ideas more clearly through a simple visualization. It is our hope that the revisions made to discussion and conclusion will improve clarity sufficiently to address this point.
